# A strengthening yet less extreme Atlantic–European jet during winter

**DOI:** 10.1038/s41561-026-02069-z

**Published:** 2026-09-10

**Authors:** Stefan Brönnimann, Yuri Brugnara, Patrick Kallabis

**Affiliations:** 1 Institute of Geography, University of Bern, Bern, Switzerland; 2 Oeschger Centre for Climate Change Research, University of Bern, Bern, Switzerland

**Keywords:** Climate change, Attribution, Atmospheric dynamics, Projection and prediction

## Abstract

The Atlantic–European jet stream is a key driver of weather extremes in Europe, yet its recent variability and trends remain poorly understood. Here we investigate daily changes in jet strength, tilt and latitudinal extent from 1725 to 2100 using pressure data from four European stations alongside climate model simulations. These indices explain about 40% of day-to-day temperature variability in Western Europe year-round. Weather extremes—including heat waves, cold spells, heavy precipitation and windstorms—are closely linked to the jet stream, and the indices also capture signals of sudden stratospheric warmings and major solar storms. Since the pre-industrial period, the jet has strengthened by 0.13 standard deviations and shifted poleward during the cold season. Future projections indicate continued strengthening in winter, while extremes in the indices become less frequent, suggesting a potential reduction in some types of weather extremes.

## Abstract (web summary)

Since 1725, weather extremes in Western Europe have been closely linked to the Atlantic–European jet stream, which is projected to strengthen by 2100 while becoming less extreme, according to daily sea-level pressure records and climate model simulations.

## Main

The jet stream over the Atlantic–European region strongly affects the weather in Western and Central Europe^1^. Temperature extremes such as cold-air outbreaks or heat waves are often related to specific configurations of the jet, while other configurations can bring heavy precipitation events^2,3^. Trends in the jet are not well understood. The jet in the North Atlantic has strengthened over the period 1951–2020, but this trend is not reproduced by climate models^4,5^. In fact, the observed strengthening is greater than in any one of the Coupled Model Intercomparison Project 6 (CMIP6) simulations^6,7^ and seems to be in large part independent of natural variability^8^. The suggested physical mechanisms behind the trend include, among others, diabatic heating^9^. A global northward shift of the jet is becoming apparent in observations over the satellite era, but this trend is mostly driven by the Asian and North Pacific sectors^10^.

CMIP6 models project a strengthening and narrowing of the North Atlantic jet in winter^11^ as a result of the tug of war between the Arctic amplification at the surface and the warming in the tropical upper troposphere^12^. A weakened Atlantic Meridional Overturning Circulation can also cause a strengthening of the northern hemispheric jet in winter^13^.

Given the large underestimation of the strengthening by CMIP6 models in the historical period, there is little confidence in the projected future trends^14,15^. In light of these results, it is crucial to analyse past changes in the jet stream variability. However, almost all studies on jet stream trends use three-dimensional data from atmospheric reanalyses such as ERA5^16^ and therefore are limited to the past few decades, whereas studies based on climate proxies only cover the seasonal timescale and the results are sometimes contradicting^14^.

**Extended Data Fig. 1:**
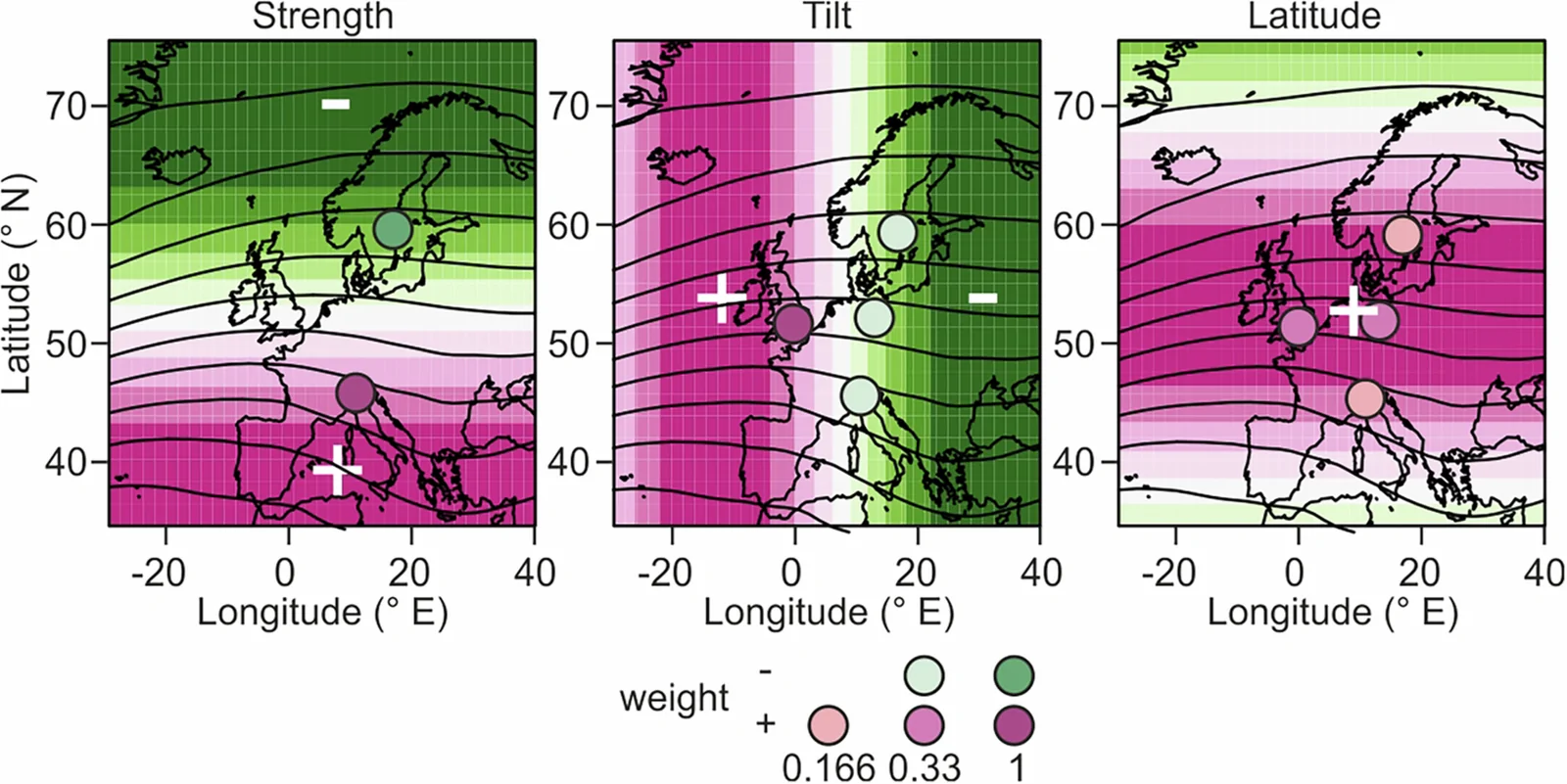
Definition of jet stream indices. Base patterns used to define strength, tilt and latitude of the jet based on monthly mean 500 hPa GPH fields. Solid lines indicate climatological mean 500 hPa GPH in ModE-RA (50 gpm contour interval). Filled circles indicate the stations used to define daily indices. The colours indicate their weight in the indices formed.

Using a monthly, three-dimensional climate reconstruction^17^, we recently analysed monthly-to-seasonal jet stream variability back to 1421 by projecting 500-hPa geopotential height (GPH) anomaly fields onto three base patterns^18^ (north–south gradient, east–west gradient and midlatitude ridge decreasing north- and southward; Extended Data Fig. 1). However, the eddy-driven jet is a transient phenomenon and studying its links to extreme weather such as storms, heavy precipitation and cold or warm extremes requires daily data, which have so far only been available for the period since around 1940. Here, we present and analyse multicentury daily jet reconstructions and compare them with data on weather extremes and with jet indices in long coupled and uncoupled climate model simulations. This allows addressing the driving forces of trends in the mean and in extremes of the jet indices.

## Constructing daily indices

**Fig. 1:**
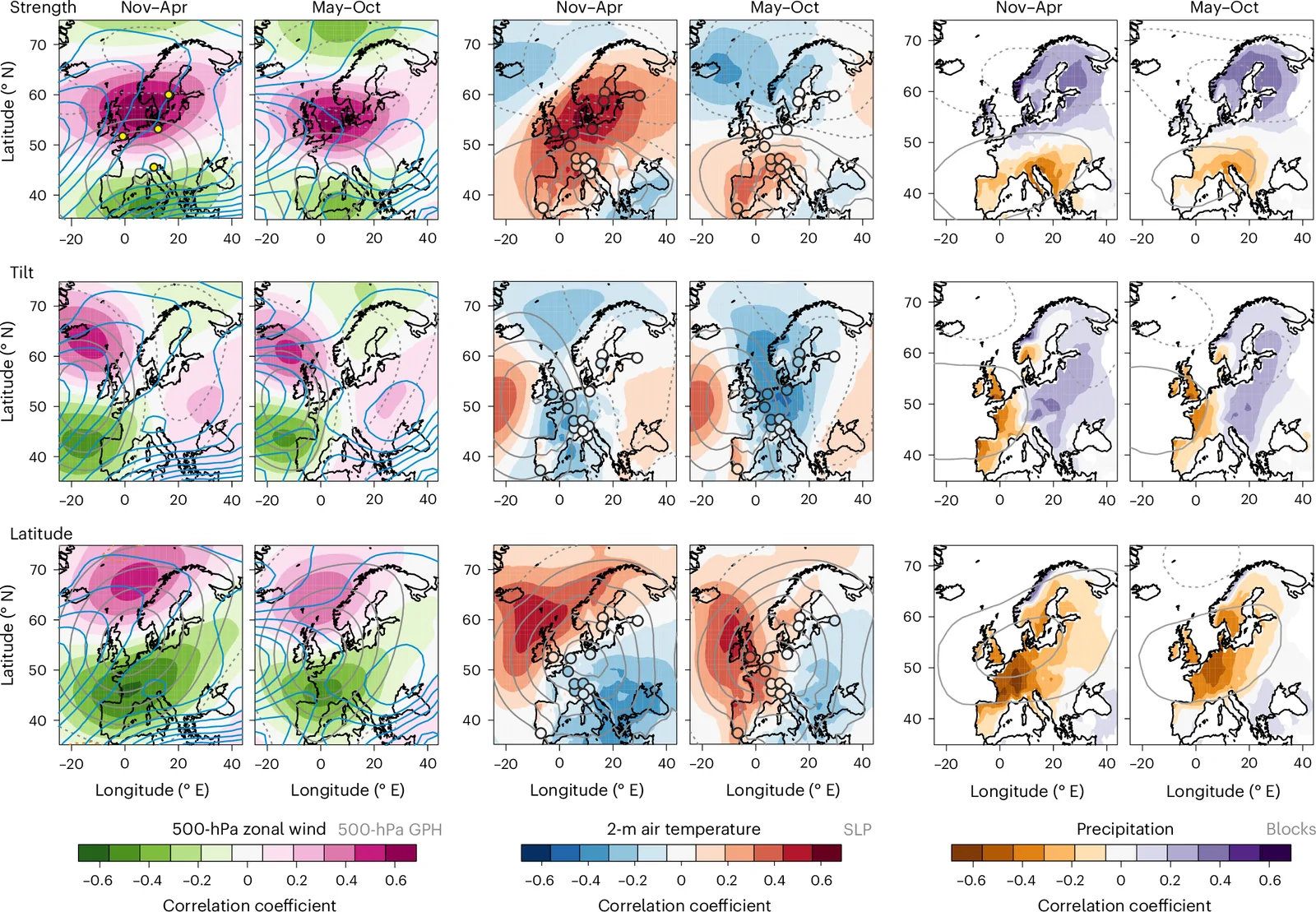
Weather patterns associated with jet stream indices. Correlation coefficients between daily indices and 500-hPa zonal wind (shading, blue contours indicate the climatology) and GPH (grey contours) (first and second column), 2-m air temperature (shading) and sea-level pressure (contour) (third and fourth column) and precipitation (shading) and blocking frequency (contours) (fifth and sixth column) in the cold and warm season, respectively. The contour interval is 0.2 centred around zero, and negatives are dashed. All analyses are based on ERA5 deseasonalized data from 1940–2020 and use the Pearson correlation except precipitation (ECA&D, 1950–2020, nondeseasonalized, Spearman correlation). Yellow dots (top left) indicate the stations used for the indices; circles indicate correlations with daily temperature observations (1765–1864).

Daily air pressure series reaching approximately 300 years back are available from London^19^, Paris^20^, De Bilt^19^, Padova^21^ and Uppsala^22^. The latter two measure the north–south pressure gradient, while London captures pressure variations in Western Europe (Fig. 1, top left, yellow dots). To capture the west–east gradient and the ridge pattern, an additional series further east is needed. In Berlin, pressure measurements started in 1721, but only data since 1876 have been available so far. We digitized and processed the earlier part of the record (Supplementary Table 1), generating a long, complete, homogeneous daily pressure record (Methods).

**Extended Data Fig. 2:**
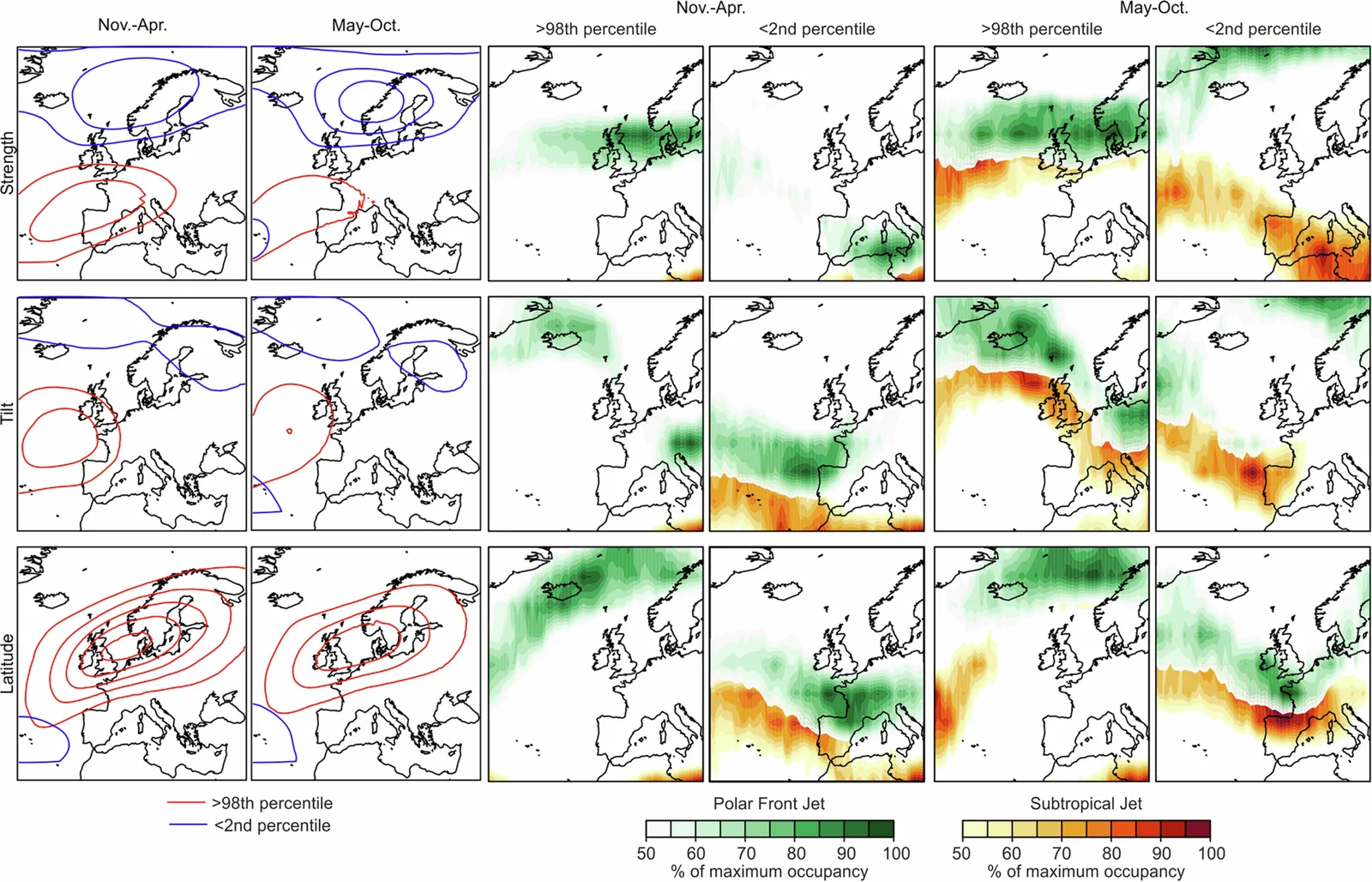
Blocking and jet position for index extremes. Blocking frequency 1806–2015 (contour interval 10%, red for positive extremes, blue for negative extremes) and the position of the polar front jet PFJ and the subtropical jet (STJ) (each field scaled to maximum occupancy in the field, PFI overprints STJ) 1941–2024 for extremes in the jet indices per season.

On the basis of the four series, we formed three indices (strength, tilt and latitude; Extended Data Fig. 1) that are largely mutually independent (Methods). Calculating them within ERA5 and correlating with 500-hPa zonal wind (Fig. 1, left) shows a monopole on the jet axis for strength, a quadrupole around the jet axis for tilt and a dipole north and south of the jet axis for jet latitude. We also analysed a dataset of position and speed of the polar front jet (PFJ) at 315 K and of the subtropical jet at 350 K covering 1941–2024^23^. We composited jet positions for indices below the 2nd or above the 98th percentile per season (Extended Data Fig. 2). For strong index cases, the PFJ is zonal, whereas for weak cases, it is diverted to the Mediterranean in the cold season (November–April) but to the North in the warm season (May–September). A positive tilt index is associated with a northwest–southeast tilt of the PFJ. The latitude index is associated with a strong latitudinal shift in the PFJ. Hence, although our indices directly measure the continental westerly flow near the surface rather than the upper-level jet, they are closely associated with strength, tilt and latitude of the Atlantic–European jet extension.

The imprint of the indices in ERA5 sea-level pressure (SLP) and 500-hPa GPH (Fig. 1) follows the three base patterns (Extended Data Fig. 1). Increased blocking frequency^24,25^ over north-central Europe accompanies a poleward-shifted jet in winter. Analysing blocking in the ‘Twentieth Century reanalysis’ (20CRv3, 1806–2015, calculated for each member, then averaged)^26^ during extremes in the indices (below 2nd or above 98th percentile; Extended Data Fig. 2) shows even more pronounced patterns, suggesting an important role for weather variability and extreme weather.

Correlations with temperature variability (based on anomalies from the mean seasonal cycle) are strong but change with season. In late winter, about half of the daily temperature variability in Denmark can be explained with the strength index (Padova minus Uppsala). This is the season when the continent is cold and the northeast–southwest temperature gradient is particularly strong (Extended Data Fig. 4). The tilt index shows the largest imprint in late spring (May), when the northern North Atlantic is cold, while the land has already warmed. Jet latitude has a large effect in summer (July) over Western Europe, when anticyclonic conditions establish (or not) over the continent. In fact, a combination of these three situations led to the coldest year in central Europe since 1421: 1740^27^. In all, with only three indices based on four pressure series, a large fraction of daily temperature variability can be explained year-round—in some locations, up to 40% (Extended Data Fig. 4).

The imprint of the indices in mean precipitation^28^ shows a relatively strong correlation over Western Europe. Unlike for temperature, there is hardly any seasonal difference.

## Drivers of extreme weather

Close relations to the jet indices are also found for extremes. We averaged indices (in this case standardized for comparability) on days with temperature, 24-h precipitation or wind speed^28^ above the 98th (below the 2nd) percentile per grid cell and plotted these averages as maps (Extended Data Fig. 5). Indices are typically outside ±2 standard deviations (roughly corresponding to the 2nd to 98th percentiles) for extreme wind speeds, ±1.5 for hot or cold days and ±1 for extreme precipitation in areas of a strong influence.

**Fig. 2:**
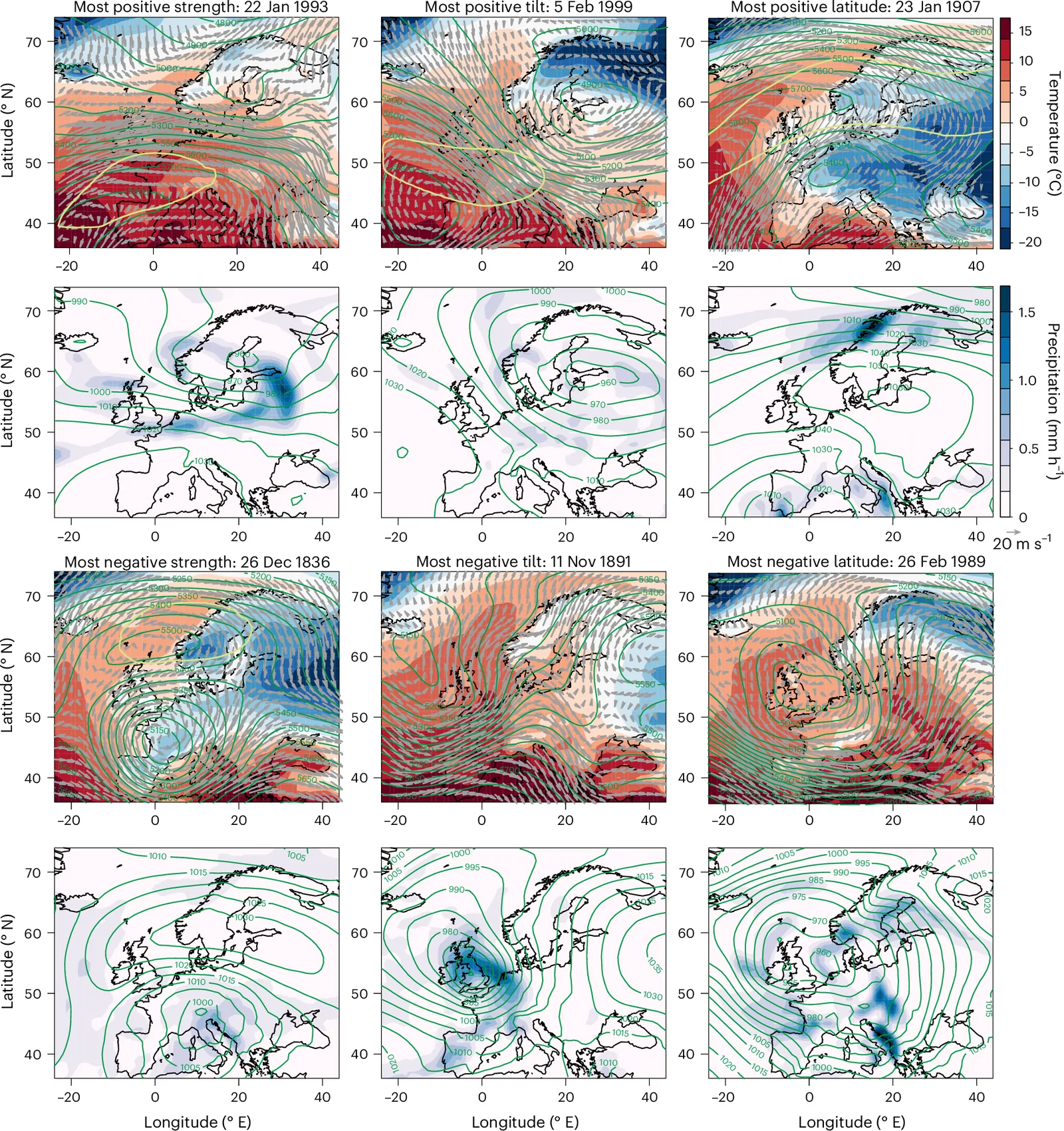
Extreme jet index days. Fields of surface air temperature, 500-hPa GPH (contours, gpm) and 300-hPa wind (arrows) (first and third row) and precipitation rate and SLP (hPa) (second and fourth row) at 12 UTC on days with the most extreme indices (Supplementary Table 3). The light green contour shows 50% blocking frequency (averaged across the ensemble).

We analysed extremes in the indices averaged over 1, 5 and 15 days. One-day events that are covered by 20CRv3 (Supplementary Table 3) are analysed for 2-m temperature, SLP, 500-hPa GPH, precipitation rate and 300-hPa wind (Fig. 2). Even though 20CRv3 has limitations in the early decades, consistent patterns of extreme weather are found. The day with the most positive strength occurred on 22 January 1993. The fields show blocking over south-western Europe and a strong pressure gradient over the North Sea. Severe storm surges hit northern Germany. The second strongest event in the 20CRv3 period was 16 February 1962, when a storm surge hit Hamburg causing the biggest natural disaster in Germany in the twentieth century. The day with the most negative strength, 26 December 1836, had strong easterly winds (>30 m s^−1^) at 300 hPa over England, embedded between two westerly jets at 70° N and 35° N, respectively, in a typical diffluent block situation. Heavy snowfall began in Southern England on 24 December and continued; snow drift formed. On 27 December 1836, an avalanche killed eight people in Lewes, East Sussex, the deadliest avalanche in history in the UK. Below freezing temperatures covered much of Spain.

The day with the most positive tilt index occurred on 5 February 1999. Strong north-westerly flow towards the Alps led to extensive snowfall, contributing to one of the worst avalanche winters in recent history in the Alps^29^. The most negative tilt, on 11 November 1891, shows strong south-westerly flow over Western Europe. The *Portland Guardian* (Friday, 13 November 1891, page 2) wrote: ‘A hurricane of fearful intensity is raging in the English Channel (…) [It] is the worst known for years’.

The situation with the highest latitude index (23 January 1907) shows the jet at 70° N, while Central Europe was under the influence of easterly flow. In Sarajevo, daily mean temperature dropped to −18.5 °C, making it the fourth coldest day on record (1901–2025), while Northern Sweden experienced exceptionally high daily mean temperatures (+0.5 °C at Jokkmokk on the next day). Finally, on 26 February 1989, the jet at 300 hPa was over the Mediterranean. With an average temperature of 11 °C, this was the warmest February day on record at Lviv, Ukraine. In the Gulf of Biscay, hurricane-force winds sank the freighter *Anna Leonhardt*; 14 people died. The analysis shows that extremes in the jet indices lead to extreme weather over Europe. It also shows that the strongest cases shown in Fig. 2 are representative for extremes in the indices (Extended Data Fig. 2) and conversely extreme indices typically accompany the discussed surface weather extremes (Extended Data Fig. 5). The effect on daily weather extremes arguably proceeds through pronounced blocking patterns.

## Stratospheric and solar influences

**Fig. 3:**
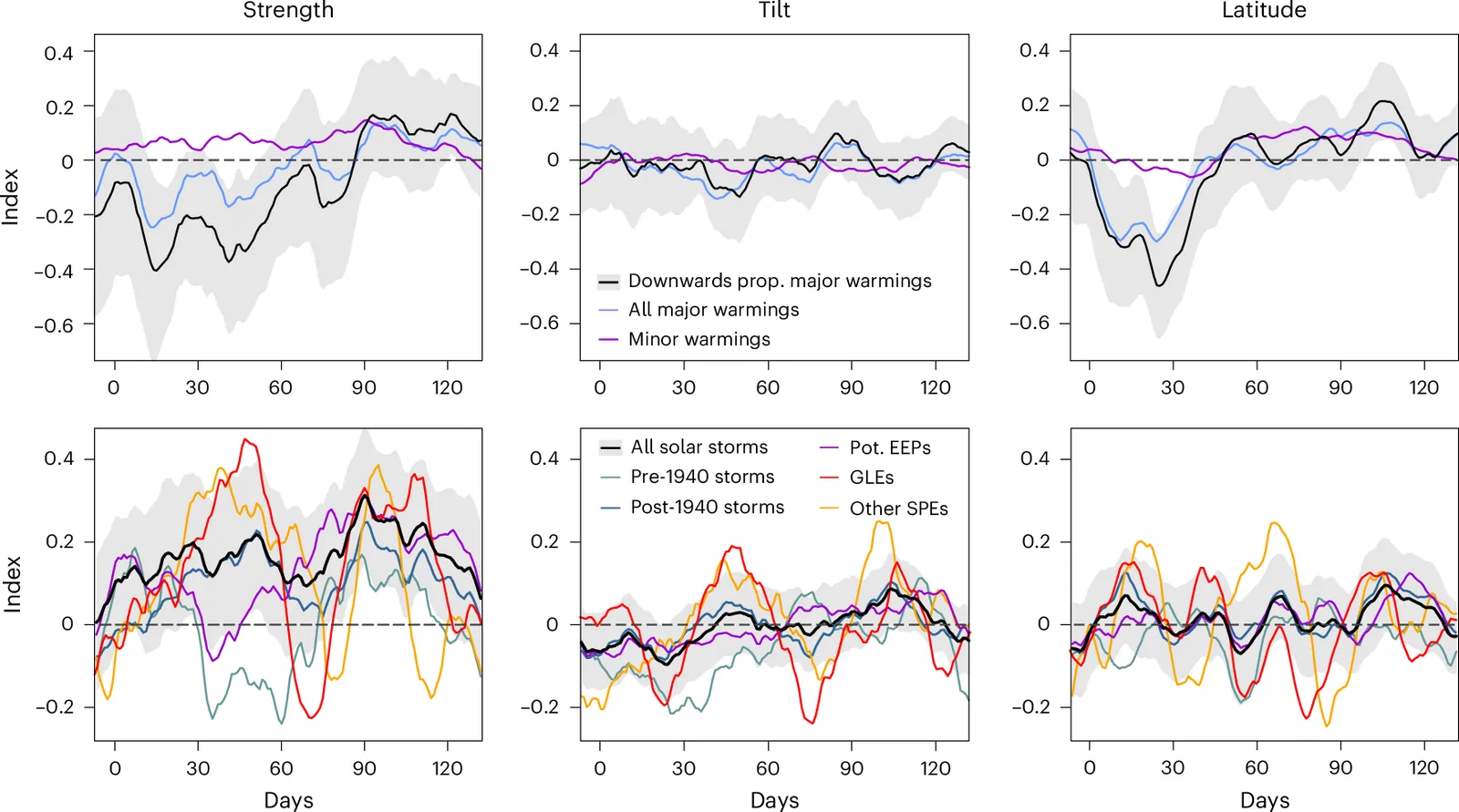
Composites of 15-day averaged indices for SSWs or solar storms. Top: composites for 55 major SSWs since 1940, of which 33 are characterized as downward propagating (prop.)^31^ (Supplementary Table 4), and 176 minor warming events. Bottom: composites for 102 solar or geomagnetic storms since 1730 that occurred in the September–March period (Supplementary Table 5) as well as for five subgroups (potential (pot.) EEPs comprise low-latitude aurorae, geomagnetic storms and fast transit events). Lines show the composite mean, and shading indicates the 95% confidence interval of the mean.

On a seasonal scale, the jet indices show imprints of volcanic eruptions and El Niño/Southern Oscillation, although internal variability dominates^14^. The daily resolution allows studying the imprint of short-term forcing effects such as sudden stratospheric warmings (SSWs) and solar storms. Major SSWs, defined as a collapse of the polar vortex in the upper stratosphere, can propagate downward by wave–mean flow interaction, eventually affecting the westerly circulation in the troposphere and extreme weather^30^. We analysed 55 major SSWs since 1940^31^ (Supplementary Table 4), of which 33 are characterized as downward propagating, and composited the 15-day filtered jet indices around the dates of the major SSWs. Results agree with previous studies^32^ and show a weakening of the jet after 10–30 days, which corresponds to the expected downward propagation time (Fig. 3). We also find a significant equatorward shift with the same delay. Both effects are the strongest for downward propagating events (minor warmings show no effect; Methods).

We then composited strong solar storms back to 1730 on the basis of historical observations (Methods; Supplementary Table 5). Solar storms are often accompanied by increased solar energetic particle fluxes (including solar proton events (SPEs) and their extreme form, ground level enhancements (GLEs)) or by geomagnetic storms that trigger energetic electron precipitation (EEP) events. Both can generate HO_*x*_ or NO_*x*_ radicals in the polar mesosphere, typically at 60–80 km (energetic SPEs also further down), leading to ozone destruction both in the mesosphere and, through NO_*x*_ transport, the stratosphere^33–35^. Associated differential atmospheric heating strengthens the stratospheric polar vortex^36^, potentially leading to a strengthened and poleward-shifted tropospheric jet^37^. A more rapid, subtropical link could also shift the jet poleward^33^. Owing to the higher altitude of the interaction, we expect a longer delay than for SSWs, presumably longer for EEP than SPEs. Sukhodolov et al.^38^ modelled the strongest known solar particle storm in 774/5 (assuming 1 September as the date of the storm) and found strengthened westerlies at the surface after 70 days. A review is given by Nesse et al.^39^.

For compositing, we focused on the active season of stratosphere–troposphere coupling, that is, winter and early spring, but accounted for possibly longer downward propagation times and considered all 102 events between September, when the mesosphere–stratosphere Arctic polar vortex typically forms, and March.

Results show a significant strengthening of the jet after 80–120 days (Fig. 3), which is robust when compositing subgroups (pre- and post-1940 events, potential EEPs, GLEs or other SPEs, each centred to the subgroup period mean for October–April); GLEs and SPEs also show earlier responses. Analyses of the strongest event per category (Extended Data Fig. 9) are consistent with these results. The jet is shifted poleward on average but not significantly. Our results show an impact of solar storms on the jet stream and thus a link between space weather and Earth’s weather.

## Changes over the last 300 years

**Fig. 4:**
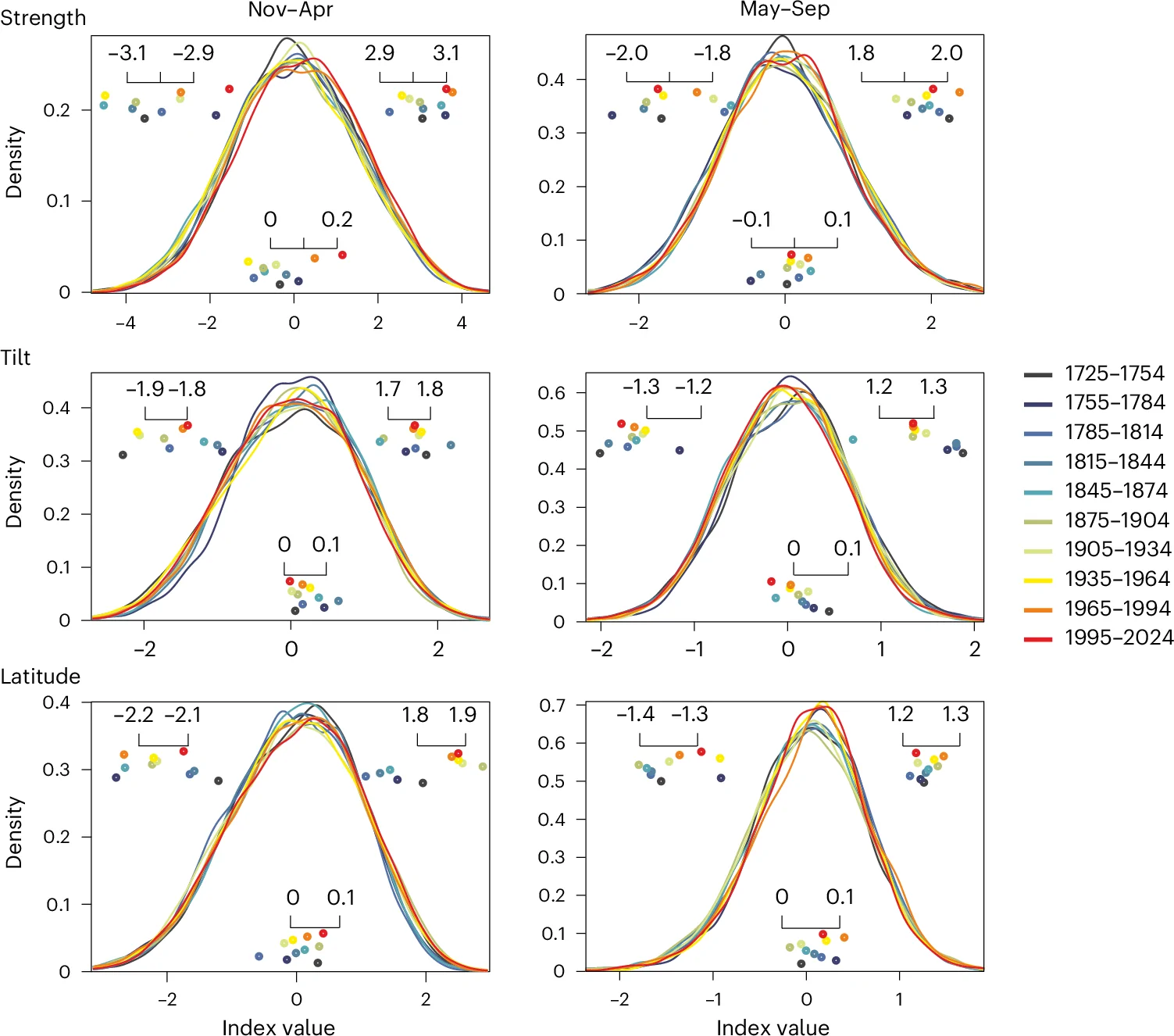
Changes in the distribution of daily jet indices. Densities of indices for the cold and warm season for ten 30-year periods between 1725 and 2024. Insets show 2nd, 50th and 98th percentiles (offset for visualization).

To address long-term changes, we analysed the distribution of the daily jet indices by dividing the 300-year period into ten 30-year periods. Changes in the jet in the last 30-year period compared with earlier periods are analysed on the basis of the 2nd, 50th and 98th percentile. Clear changes are seen in the jet strength in the cold season (Fig. 4). The 2nd and 50th percentiles move upwards, pointing to a stronger jet with fewer negative extremes. The increase in the median since the pre-industrial period corresponds to 0.13 standard deviations of daily indices (1851–1950), and the increase of the 2nd percentile is even stronger (0.16 standard deviations). There were not more positive extremes in the last period compared with the previous 30-year period but more in the last 60 years compared with all earlier periods. The median of the tilt index decreased in both seasons (most clearly in the warm season), and the latitude index increased in the cold season. Positive extremes in the tilt index in the warm season decreased. Corresponding analyses for 5- and 15-day averages (Extended Data Fig. 6) show similar results. These changes are consistent with an observed strengthening of the jet reported in literature, although it should be noted that most literature analyses the upper-level jet, whereas our diagnostics describe the surface imprint.

**Fig. 5:**
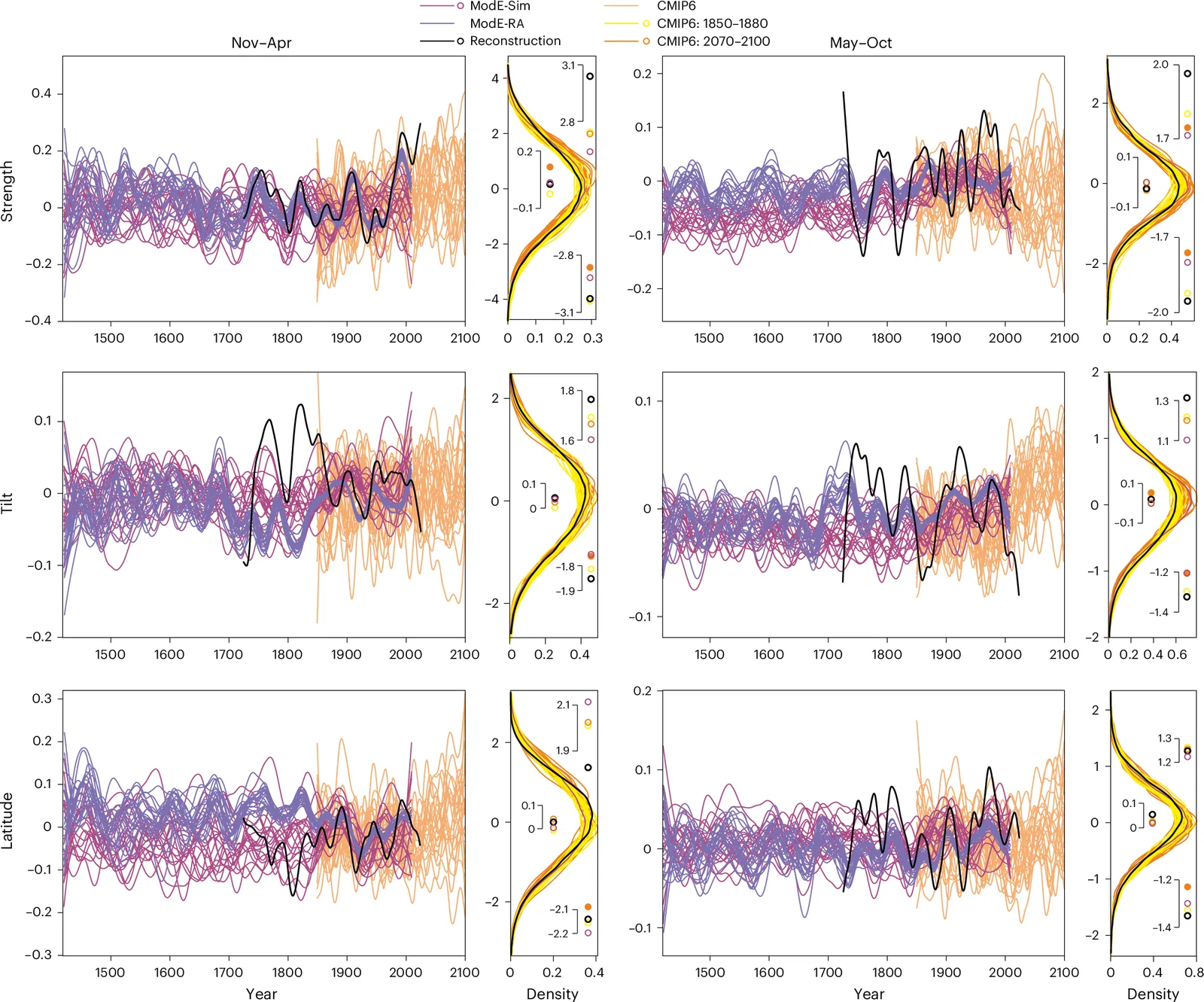
Development of the jet stream. Time series of seasonal mean jet strength, tilt and latitude in different datasets (reconstruction, ModE-RA, ModE-Sim, CMIP6 historical and SSP2-4.5) smoothed with a spline fit. ModE-RA indices were scaled to match the ModE-Sim standard deviation over the 1851–1950 period. The marginal distributions (note the different scale) show the densities of the daily indices (as in Fig. 4) for the observations (entire period), ModE-Sim (1851–1880) and CMIP6 (1851–1880 and 2071–2100). Insets with coloured dots show the means of the 2nd, 50th and 98th percentiles of the distribution, and filled orange symbols denote a significant difference between 1851–1880 and 2070–2100 across the different model simulations.

Time series of seasonal means (Fig. 5; smoothed with a spline) confirm the increased cold-season strength, the strongly positive tilt in the eighteenth and early nineteenth century and an increasing cold-season latitude. The agreement of the indices with similarly filtered monthly indices^18^ at 500 hPa from ModE-RA (ensemble of 20)^17^ is good for cold-season jet strength but less good for other seasons and indices. Daily indices were also calculated from the ensemble of 20 atmospheric simulations underlying ModE-RA, that is, ModE-Sim^40^. Variations in common with ModE-RA indicate variability constrained by the model boundary conditions (for example, sea-surface temperatures, volcanic aerosols and greenhouse gases).

Unlike for the 500-hPa indices, where all recent changes are within the variability of the past 600 years, the recent strength in the daily index in winter is outside the variability of the past 300 years and outside the ModE-Sim ensemble of the last 600 years. The same is true for the low values of tilt in the warm season. However, this comparison should be taken with care. Although all daily pressure series in observations and model simulations were standardized on the basis of the same period, the variability of the smoothed indices differs between datasets. This points to differences in the amplitude of variability at scales larger than daily. Strong excursions in the eighteenth century could point to remaining errors in the pressure measurements.

Next, we compared the observation-based indices to indices derived from the historical simulations from the Climate Model Intercomparison Project (CMIP6)^41^ (Supplementary Table 6). Their smoothed time series exhibit a higher variability than ModE-Sim, mostly encompassing the observations. The observed recent jet strengthening is at the upper edge of the distribution of CMIP6 historical but outside of the distribution of ModE-Sim. The distributions (right insets) show that ModE-Sim and CMIP6 historical both underestimate the 98th percentile for strength and tilt in both seasons, but both overestimate the 98th percentile for jet latitude in the cold season.

## Future changes in jet stream indices

To assess future changes in the jet, we analysed the SSP2-4.5 scenario (results for the SSP3-7.0 and SSP5-8.5 scenarios are shown in Extended Data Figs. 7 and 8, respectively) and compared the two 30-year periods 1851–1880 and 2071–2100. The jet will get stronger in the cold season and more tilted in the warm season, whereas the other indices and seasons show no significant trends. However, we find increasing trends in the 2nd percentile and decreasing trends in the 98th percentile in all seasons and indices; many of them (including all six trends of the 2nd percentile) are significant. This indicates that the jet gets less extreme. This result is the same for all scenarios; in the stronger scenarios, the magnitudes of the trends are stronger and we additionally find a poleward shift of the median in the cold season. However, CMIP6 models have been shown to exhibit inconsistencies, which therefore need to be interpreted with care.

The projected strengthening of the cold-season jet is in line with the observed changes in the recent decades^4–9^. However, it is not seen in the warm season and it is not seen in extremes. The upper-level jet depends on the temperature gradient near the tropopause, which increases as the tropical upper troposphere warms and the Arctic lower stratosphere cools and might explain the increased strength^12^. This is however not the case for the surface, where temperature gradients decrease. The vertical structure of the trend as well as the consistent decrease in extremes across all indices and seasons in the CMIP6 scenario simulations (and partly in the observations) therefore requires further investigation.

## Methods

### Uppsala, Padova and London pressure series

The Uppsala daily pressure series was taken from the IMPROVE project^22^ from 1722 to 2000 and from Uppsala University (SMHI) from January 2001 to September 2023. For gap filling and for comparison, we consulted a series from Stockholm, 1756–2012, from the IMPROVE project^42^. We found sometimes large differences of up to 30 hPa or more despite the spatial proximity. We analysed all differences larger than 10 hPa in comparison with the days before and after and with neighbouring stations. This led to flagging and removing 565 values in the Uppsala series and 32 in the Stockholm series.

The Padova daily pressure series was taken from the IMPROVE project^21^ and covers 1725–1997. For homogenization, we applied the published correction amounts and techniques^21^. We used station data from Rovereto (1782–1849) and Milan (1763–1862)^43^ to fill gaps in the next step. The data from December 1997 to December 2009 were taken from Treviso (ISPD) and from January 2010 to September 2023 from Teolo (ARPAV).

The London daily pressure series was based on homogenized subdaily data^19^, covering 1691–2023. The data were averaged to daily means and then merged by file number.

### Berlin pressure series

The daily Berlin pressure series consists of data from several segments presented in Supplementary Table 1. The longest historical segments are those from the Kirch family, 1721–1774^44^, and Brand, 1760–1794, whereas the data from the German weather service (DWD) start only in 1876. Earlier gaps and gaps between the Brand series and the DWD series were filled with series from Jablonski, 1725–1727; Lambert, 1769–1777; and Gronau, 1775–1801, from Berlin as well as several other series from nearby cities, including Loebau, 1720–1730; Wittenberg, 1728–1729; Žitenice, 1807–1818; and Jena, 1824–1990. The 6-year gap between Žitenice and Jena was filled with data from 20CRv3. Because of an obvious drop in the variance in this period owing to the fact that the ensemble mean is too smooth, we inflated the variance in 20CRv3 by removing the seasonal cycle, multiplying the residual with the ratio of the standard deviations of the observations and 20CRv3 and adding back the seasonal cycle.

### Auxiliary pressure series

The following auxiliary pressure series were used to fill gaps in the data series. This was most relevant in the eighteenth century, when gaps were the most frequent. We used a series from De Bilt, 1743 to present^19^, which we back-extended to 1738 (Zwanenburg), and a daily pressure series from Paris^20^. Further, two series from Nuremberg were used^27^, covering 1722–1743. From Switzerland, we used the series from Zurich (1718–1733) and Neuchâtel (1753–1782)^45^. A series from Montpellier (1706–1748) was also used. However, this series is clearly of lower quality and consists to a considerable extent of extrema, that is, we have typically approximately six values per month, which always include the lowest and highest pressure. For testing and homogenizing this dataset, we first used the analogue-resampling and off-line Ensemble Kalman Filter technique described below to generate a daily reference series. We found a correlation of this reference series with the (independent) observations of 0.55 on the basis of deseasonalized data. Then, we used this reference for breakpoint detection and homogenization (see below).

### Pre-processing and testing

In the first step, all series were averaged to daily means (if subdaily). We then performed a sequence of outlier tests, marking measurements (1) outside the interval 950–1060 hPa, (2) outside the interval −5/+4 standard deviations (as negative extremes are more common), (3) steps of 30 hPa or more in a day and (4) four or more consecutive identical values. All 2,761 marked measurements were analysed individually by analysing two or more time steps before and after and by analysing all available stations. Most of the marked measurements were kept, as they were well in line with the other stations. Only about 5% were eventually flagged.

After this procedure, Craddock and Pettitt tests were performed on all series. The former is a graphical test^46^ based on the cumulative sum of differences to a reference series, whose shape indicates the type (step or trend) of an inhomogeneity as well as the breakpoints; the latter is a non-parametric test for breakpoints^47^. The series of Uppsala, Padova and London had already been homogenized by other authors and tests confirmed their homogeneity. The Berlin series was homogenized (that is, adjusted for breaks) by using several reference series from DWD after 1876 and from the HCLIM^48^ dataset before that. Each segment in Supplementary Table 1 was first homogenized independently and then merged with the others by proceeding backward in time and adjusting the older segment according to the overlap with the more recent one (in one case, the Prague series from HCLIM was used as reference instead because the overlap was too short). The list of corrected breakpoints (detected using the Craddock test with the support of some metadata such as station relocations or instrument changes) is presented in Supplementary Table 7. The corrections were calculated for each calendar month as the medians across all reference series and then smoothed to the calendar days by fitting the first two harmonics of a Fourier series to avoid jumps between months. In two cases, large breakpoints were separated by only a few months (in 1760/61 in the Kirch series and 1794 in the Gronau series), leaving insufficient data for a reliable correction; the data between those breakpoints were considered invalid and removed.

Furthermore, also some of the auxiliary series were found to be inhomogeneous and were homogenized. Neuchâtel was corrected by −3.06 hPa before 1/1765. Afterwards, the entire series was detrended (adding −0.58 hPa yr^−1^), as the Craddock test revealed a trend inhomogeneity. The Zurich series was corrected by −4.87 hPa before 1/1729. For both Neuchâtel and Zurich, we used the London series to determine the correction, but the inhomogeneities were also found when using Padova, Paris, Uppsala or De Bilt as reference. After homogenization, the two series were standardized separately and then merged. The Montpellier series showed a likely inhomogeneity on 1 May 1741, which we corrected on the basis of the generated reference series.

For the further procedure, all series were deseasonalized and standardized (DS) by subtracting the first two harmonics of the seasonal cycle and dividing the residuals by the standard deviation on the basis of the entire available respective time period. This means that the series of Zwanenburg and De Bilt were standardized individually, as were the two series from Nuremberg. Therefore, no homogenization is required in these cases.

### Gap filling

The further processing was based on DS data because the index series will later be defined on the basis of DS data and because there might be biases in the mean, in the seasonal cycle (through a temperature dependence) and in the variability (owing to the number of measurements per day). First, we filled gaps by means of neighbouring stations (Stockholm for Uppsala, Rovereto or Milan for Padova) on the basis of DS data and then back-converted them to absolute values using the coefficients and standard deviations of Padova and Uppsala, respectively (Supplementary Table 8). Then we proceeded similarly and filled remaining gaps with DS reanalysis data from the closest grid cell at 12 UTC. From here onwards, we used 1851–1950 as a base period for standardization (1940–1989 for ERA5). Gaps were filled (also in the auxiliary series) with data from ERA5 if the gap was between 1940 and 2024, or from 20CRv3 if it was earlier. After this procedure, a small fraction of gaps remained (Supplementary Table 8). For filling these gaps, we used an analogue method augmented with an off-line Ensemble Kalman Filter approach^49^. On the basis of all series (including DS auxiliary series), we searched for the best analogue in the 1940–2024 period using the Euclidean distance. The best analogue was further augmented using an off-line Ensemble Kalman Filter, with covariances specified from the best 200 analogues. The observation error covariance *R* was set to 0.16, which corresponds to an observation error of approximately 4 hPa (the same approach was used for generating a reference dataset for the Montpellier pressure series). To estimate the skill of the in-filling, we evaluated the procedure in the year 1868, alternatingly reconstructing each series from its neighbours. We found relatively high correlations (Supplementary Table 8), except for Uppsala (*r* = 0.56), which (after merging with Stockholm, 20CRv3 and ERA5) had only two missing days in the past 300 years. Note that the gap filling only concerns a few values (most of them in the Berlin series), mostly in the 1720s to 1750s as well as some in the 1760s and 1770s. The two homogenized series from Paris and De Bilt contribute the most to filling gaps, followed by the series from Neuchâtel (each contributes to fill more than 1,000 gaps). The two series from Nuremberg and Zurich contribute very little (each less than 90 filled gaps), as they do not overlap with periods in which many gaps were observed.

We provide the pressure series in different formats, including the quality-controlled, homogenized data and their quality flags (for all series, including the auxiliary series), the gap-filled standardized data and the gap-filled data converted back to pressure using the standard deviations and coefficients of the four series.

### Climate models

We selected all CMIP6 models that provide SLP and that have no missing years. We selected the historical runs and the scenarios SSP2-4.5, SSP3-7.0 and SSP5-8.5. For each scenario, we only used models that have both the historical run and the corresponding scenario run, and only one run was selected per model. The models and simulations are presented in Supplementary Table 6. Likewise, we used all 20 ensemble members from ModE-Sim (sets 1420-3 and 1850-1).

In each simulation, we extracted the grid points closest to the locations of Padova, Uppsala, London and Berlin. For forming the indices, we subtracted the first two harmonics of the seasonal cycle and divided the residuals by the standard deviation (both derived from the 1851–1950 period). Significance between the periods across the model simulations was calculated with a Wilcoxon rank sum test (which is equivalent to the Mann–Whitney test).

### Indices

The indices are defined in a similar way as other pressure indices (for example, the North Atlantic Oscillation (NAO) index) as differences between (or sums of) standardized, deseasonalized pressure series:

Strength = *P*_Padova_ – *P*_Uppsala_

Tilt = *P*_London_ – 0.33 × (*P*_Berlin_ + *P*_Padova_ + *P*_Uppsala_)

Latitude = 0.33 × (*P*_London_ + *P*_Berlin_) + 0.1667 × (*P*_Padova_ + *P*_Uppsala_).

All indices were standardized on the basis of the 1851–1950 period (except in ERA5, where we used 1940–1989). The three indices are largely uncorrelated (correlation coefficients over all seasons and years are 0.13 for strength and tilt as well as for tilt and latitude and 0.07 for strength and latitude). Correlations with a daily NAO index from 1836 to 2015^50^ in the cold season are 0.393, 0.175 and 0.142 for strength, tilt and latitude indices. This indicates that our indices are related to but different from the classical NAO, arguably owing to the different longitude (Uppsala is 17.5° E in the jet extension region, Reykjavik is 22° W in the core region of the jet).

To address longer timescales, we used a moving average of 5 and 15 days, respectively. Averaging the daily indices to monthly means and then comparing them with the indices derived from monthly ERA5 500-hPa GPH fields yields high correlations (approximately 0.75) for strength and tilt in both seasons but lower (approximately 0.62) for jet latitude. There is no one-to-one relation to monthly indices of the NAO, the East Atlantic Pattern (EA) and the Scandinavian pattern (SCAND) of the National Oceanic and Atmospheric Administration (NOAA) Climate Prediction Centre^51^. The jet strength is weakly (0.31) correlated with the NAO and more strongly (−0.51) with SCAND, tilt is negatively correlated with both NAO (−0.44) and EA (−0.45), whereas latitude is weakly (0.28) correlated with EA.

We also compared the indices with independent monthly frequencies of wind direction over the English Channel^52^ back to 1685 (Supplementary Table 2) and found positive (negative) correlations of the jet strength index with the fraction of days per month with westerly (northerly and easterly) wind. Jet tilt is positively (negatively) correlated with the fraction of northerly (southerly) winds, while jet latitude is positively (negatively) correlated with the fraction of easterly (westerly) wind. Correlations are not very strong (±0.3–0.4) but consistent over time and season.

**Extended Data Fig. 3:**
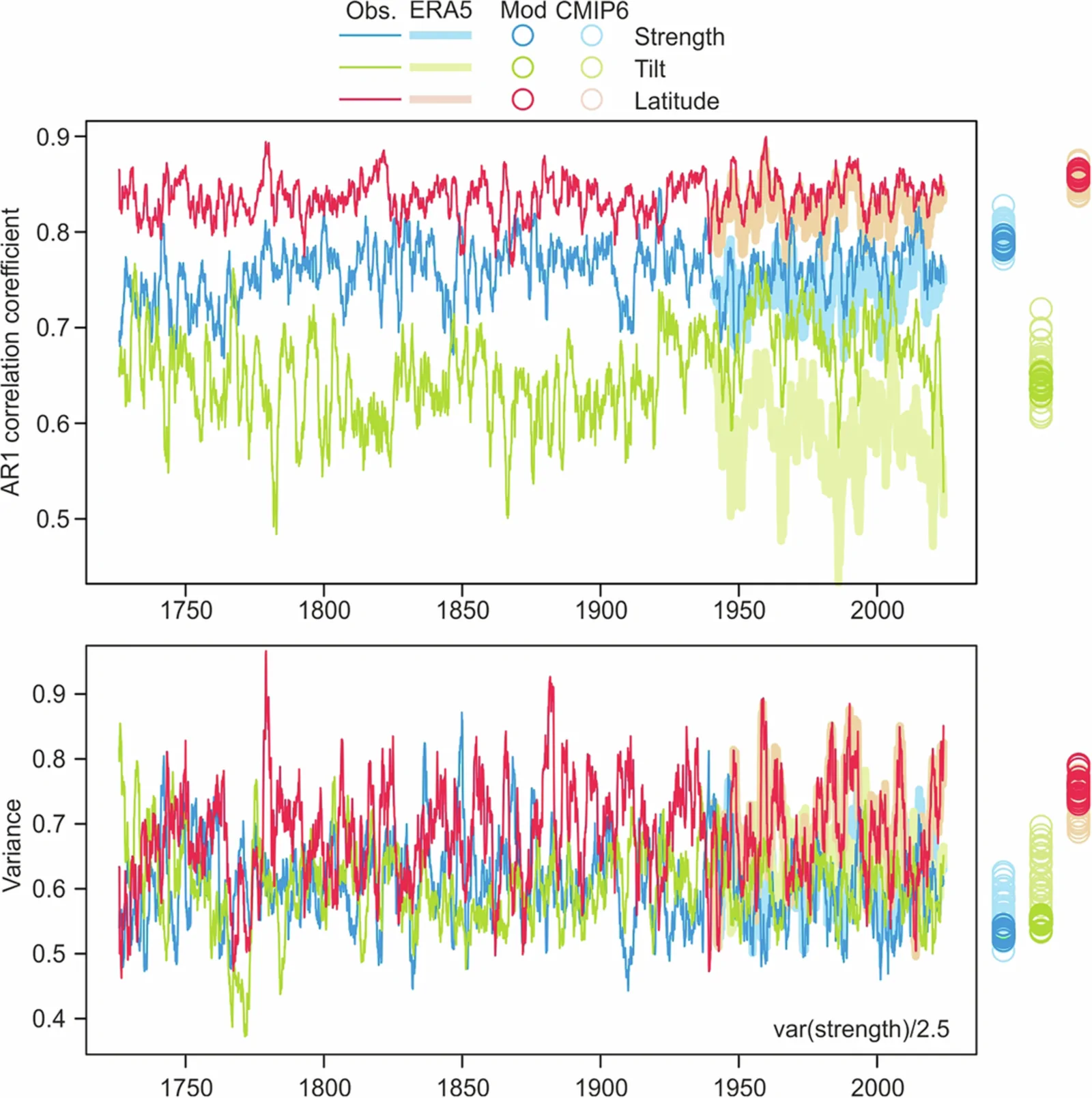
Series of lag-1 autocorrelation and variance of the jet stream indices. 2-yr moving time series of (top) lag-1 autocorrelation and (bottom) variance of the three jet stream indices in the daily reconstruction and ERA5. Dots indicate corresponding lag-1 autocorrelations and variances in each member of ModE-Sim (Mod.) and CMIP6 historical simulations.

**Extended Data Fig. 4:**
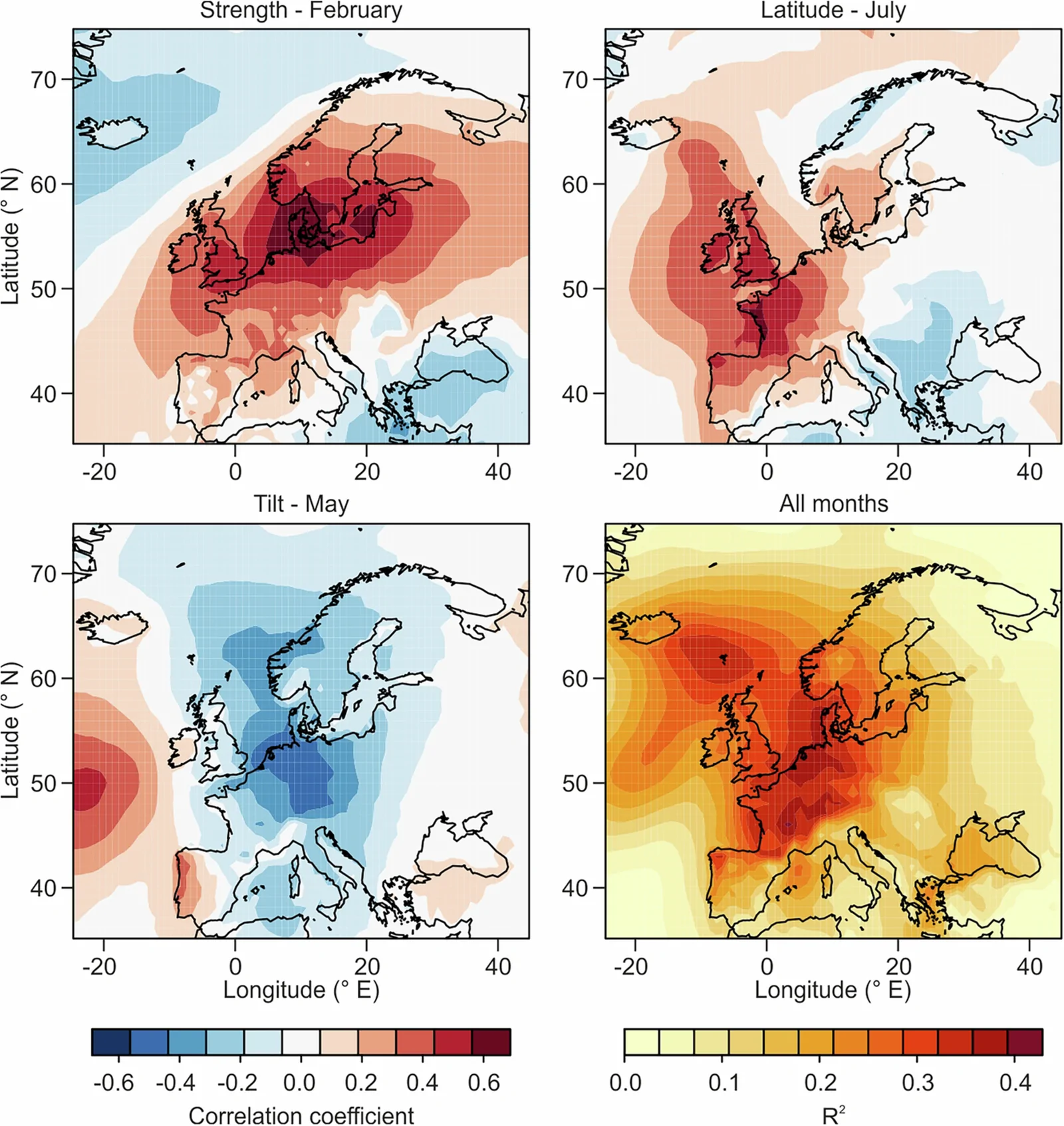
Correlation of 2-m-temperature with jet indices. The figure shows, per index, the correlation field in the months with the strongest correlation. The bottom right figure shows the explained variance of a regression of daily temperature fields onto the three indices (with a different model for every calendar month).

**Extended Data Fig. 5:**
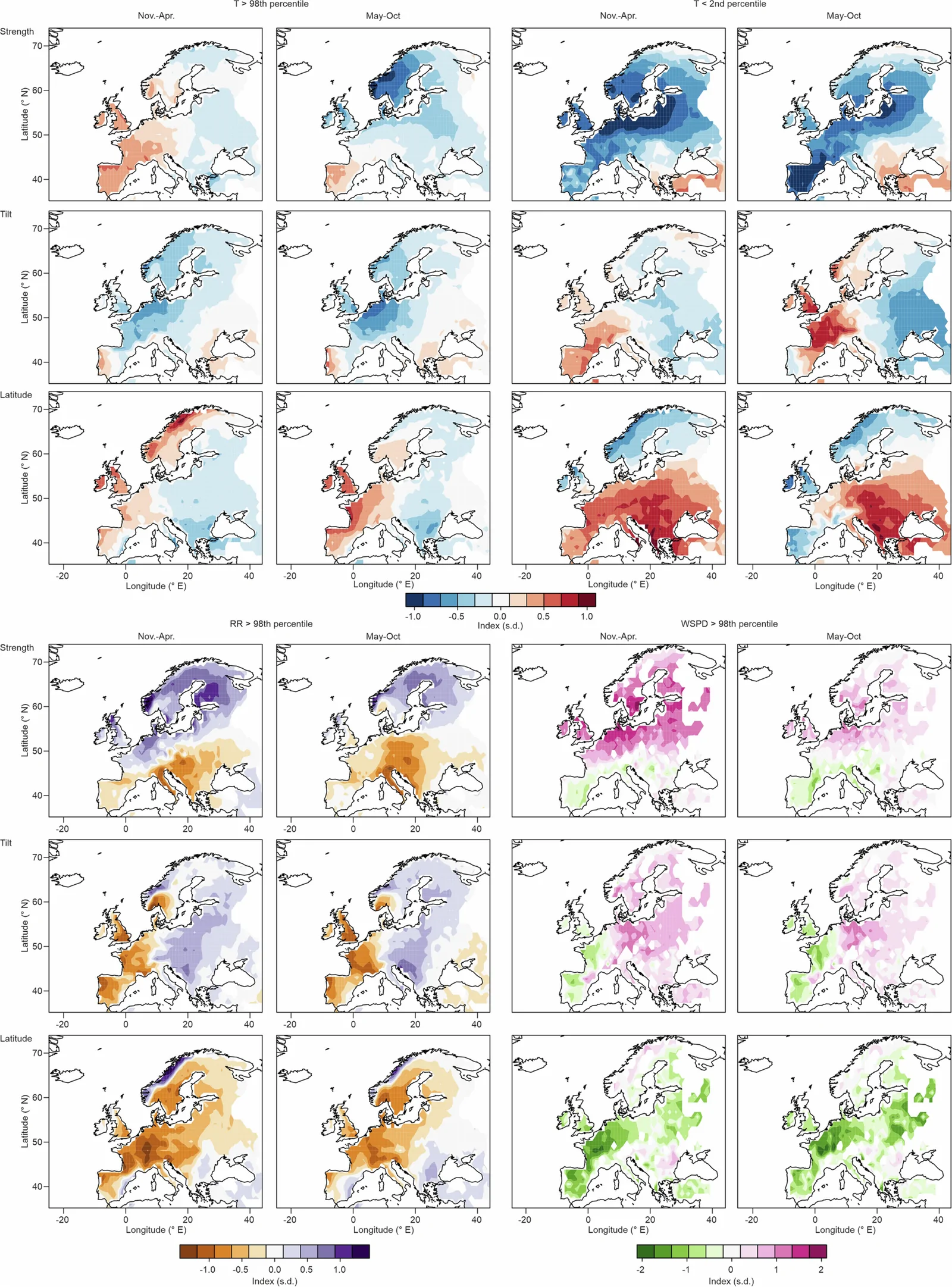
Mean indices for extreme weather events. Average of the jet indices for all days with (top) daily mean temperature (deseasonalised) above the 98^th^ or below the 2^nd^ percentile and (bottom) 24-h precipitation and wind speed above the 98^th^ percentile per season and grid cell. All data are from E-Obs and cover 1950–2024 (wind speed 1980–2024).

**Extended Data Fig. 6:**
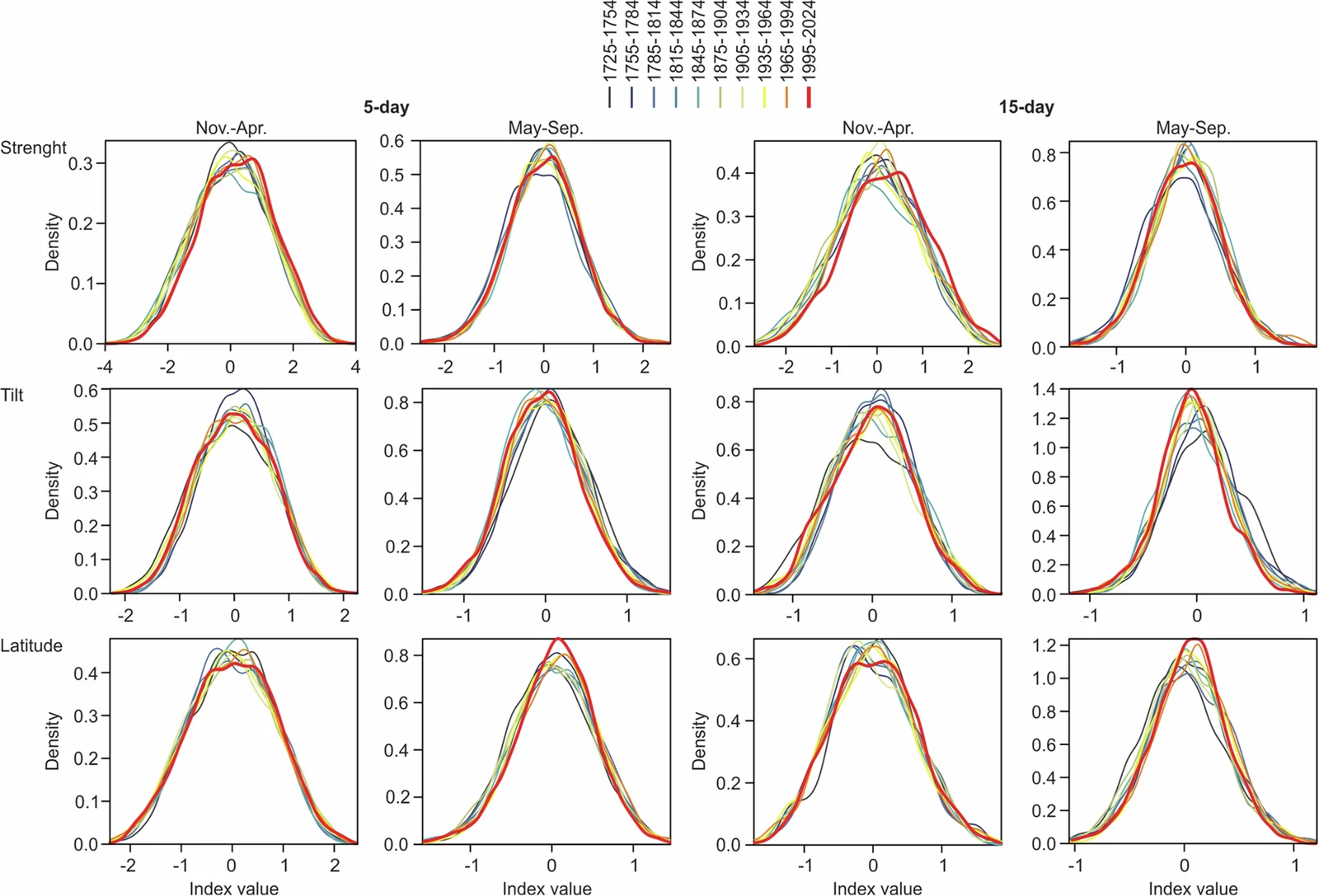
Changes in the distribution of 5-day and 15-day averaged jet indices. Densities of jet indices for the cold and warm season for ten 30-yr periods between 1725 and 2024.

**Extended Data Fig. 7:**
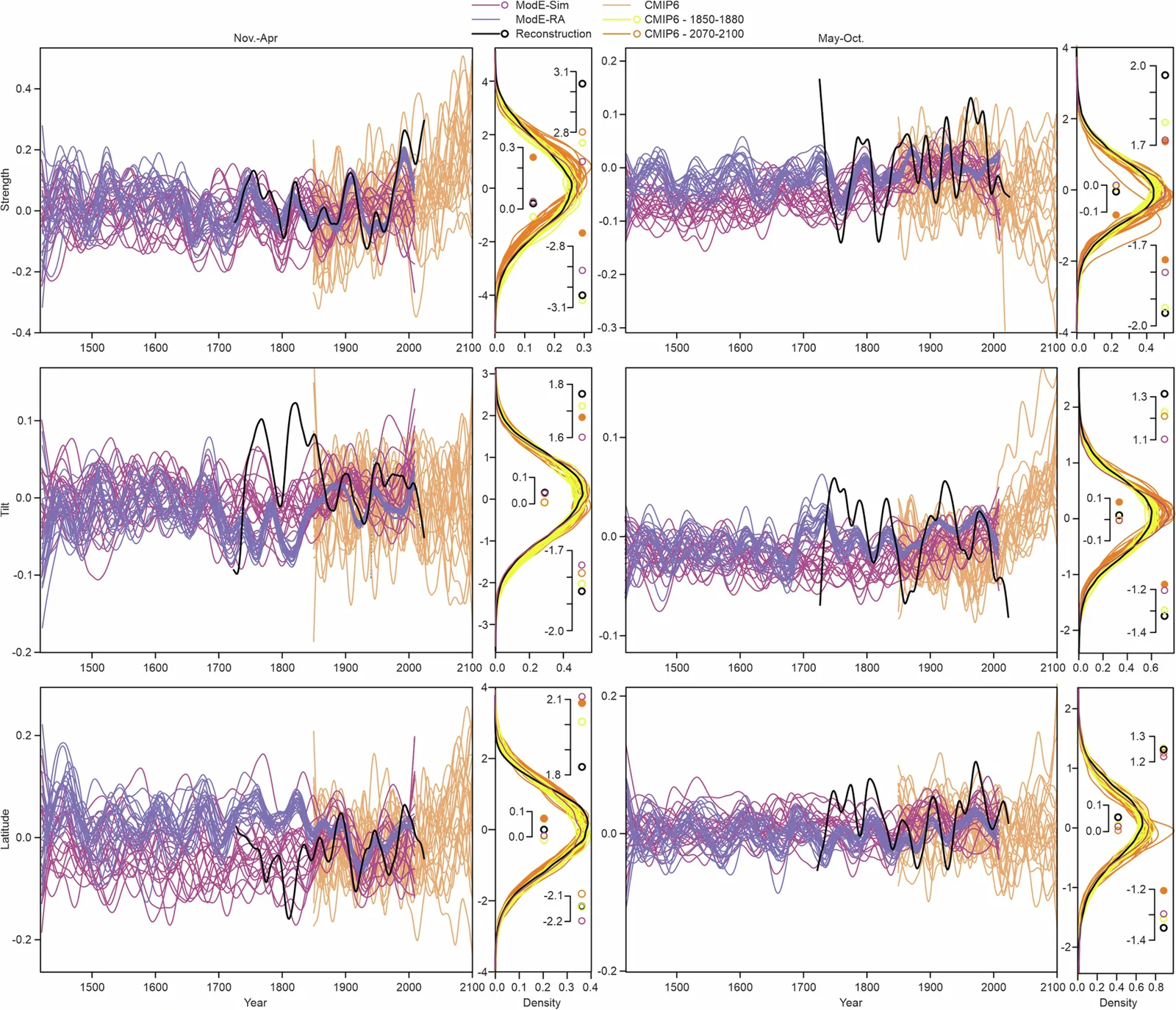
Development of the jet stream using SSP3-7.0. Time series of seasonal mean jet strength, tilt, and latitude in different data sets (reconstruction, ModE-RA, ModE-Sim, CMIP6 historical and SSP3-7.0) smoothed with a spline fit. ModE-RA indices were scaled to match the ModE-Sim standard deviation. The marginal distributions (note the different scale) show the densities of the daily indices (as in Fig. 4) for the observations (entire period), ModE-Sim (1851–1900) and CMIP6 (1851–1880 and 2071–2100). Insets with coloured dots show the means of the corresponding 2^nd^, 50^th^ and 98^th^ percentiles of the distribution, filled orange symbols denote significant difference between 1851–1880 and 2070–2100 across the different model simulations.

**Extended Data Fig. 8:**
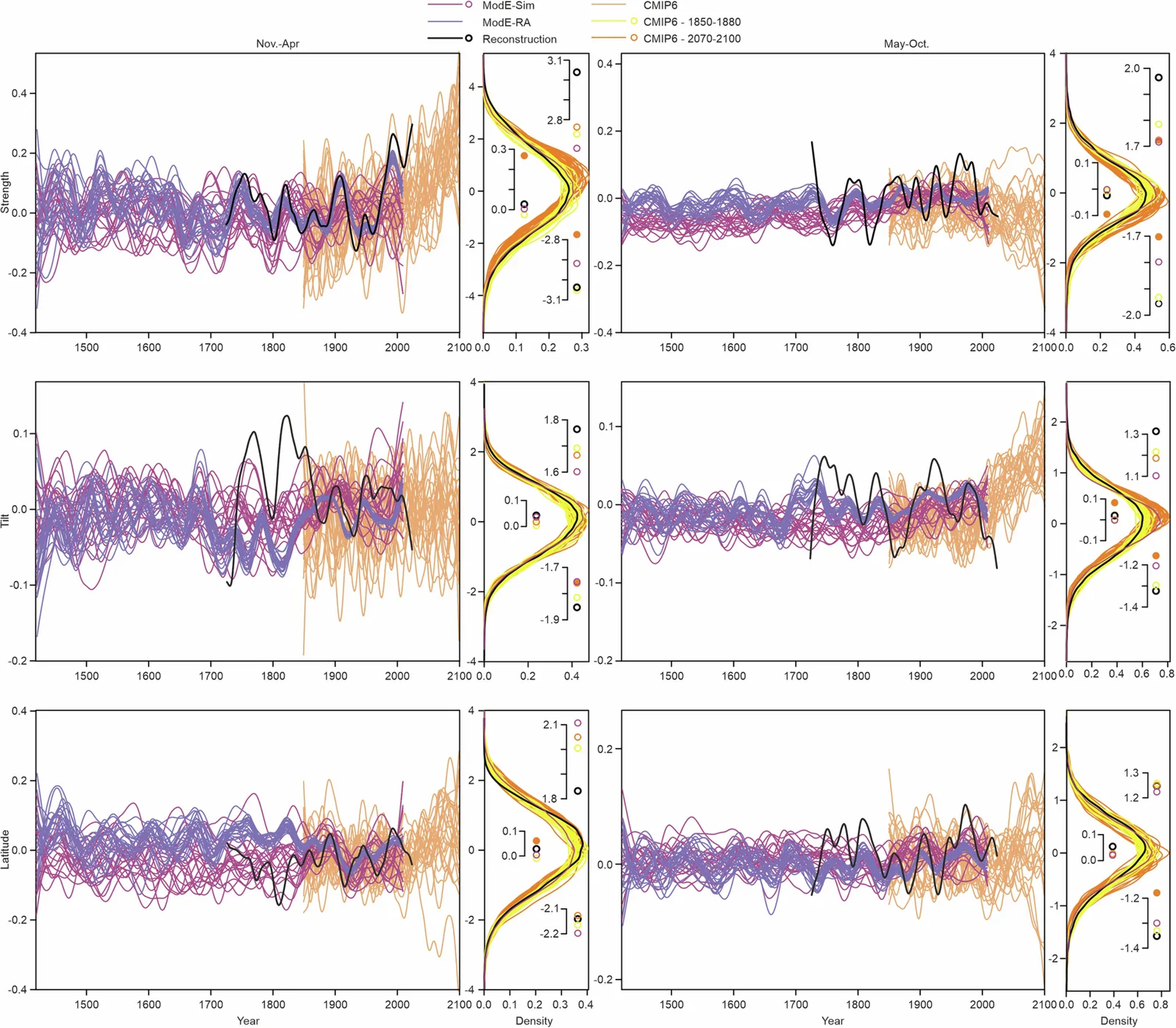
Development of the jet stream using SSP5-8.5. Time series of seasonal mean jet strength, tilt, and latitude in different data sets (reconstruction, ModE-RA, ModE-Sim, CMIP6 historical and SSP5-8.5) smoothed with a spline fit. ModE-RA indices were scaled to match the ModE-Sim standard deviation. The marginal distributions (note the different scale) show the densities of the daily indices (as in Fig. 4) for the observations (entire period), ModE-Sim (1851–1900) and CMIP6 (1851–1880 and 2071–2100). Insets with coloured dots show the means of the corresponding 2^nd^, 50^th^ and 98^th^ percentiles of the distribution, filled orange symbols denote significant difference between 1851–1880 and 2070–2100 across the different model simulations.

**Extended Data Fig. 9:**
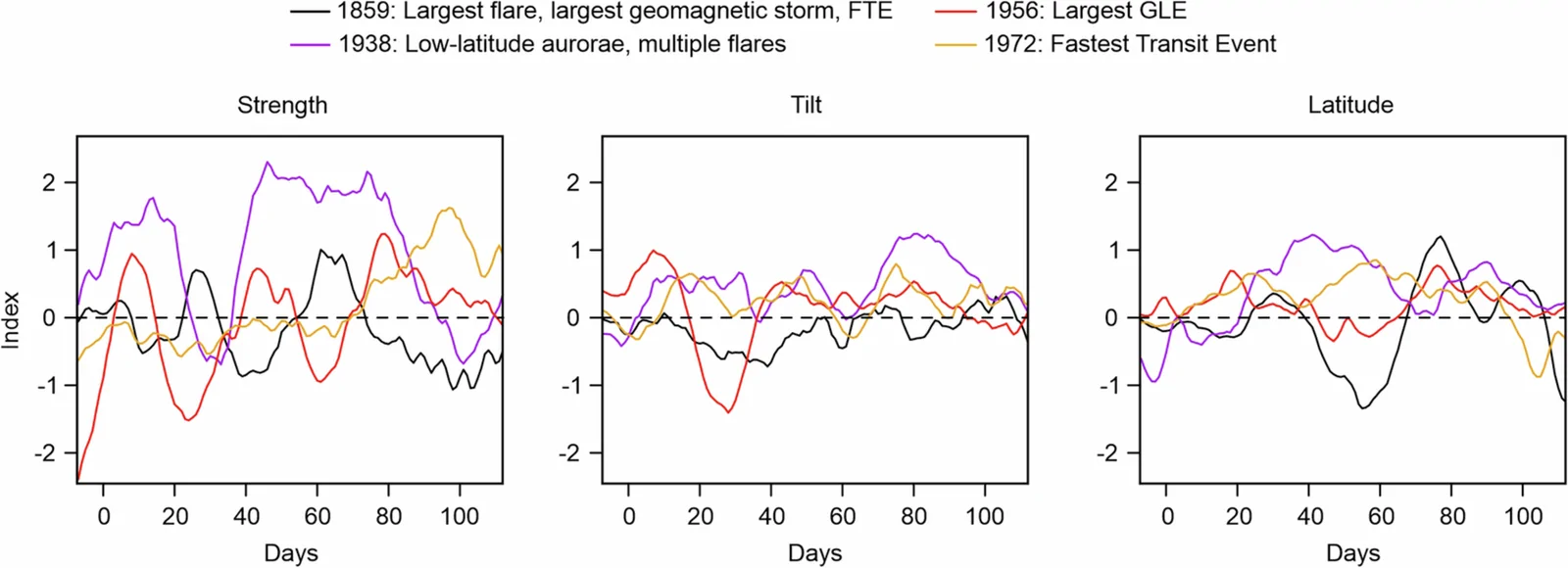
15-day averaged indices for the strongest solar storms. Shown are the Carrington event on 1 Sep 1859 (largest geomagnetic storm)^65–68^, the low-latitude aurora 16 Jan 1938^69^, GLE #5 on 23 Feb 1956^70^, and the fastest-transit event on 4 Aug 1972^71^.

The three series are stationary over time. Two-year moving time series of lag-1 autocorrelation and of the variance do not reveal obvious changes (Extended Data Fig. 3). The autocorrelation for the tilt index is higher in the station-based indices than in ERA5, but this also the case for all model simulations. A lower quality of pressure measurements is expected in the early decades when instruments were poorer, procedures were not standardized, fewer redundant information was available for quality control and there were no good reference series for homogenization.

To confirm that the indices explain day-to-day temperature variability also in the past, we used long daily temperature series from 14 sites for 1765–1864, a period with good data coverage and calculated anomalies from the mean seasonal cycle based on this period: Bologna^53^, Padova^54^, Milan^43^, Cadiz^55^, Berlin^56^, the Central England temperature^57^, the Swiss Plateau temperature series^58^, Geneva and Basel^59^, Paris^60^, Uppsala^22^, Stockholm^42^, St. Petersburg^61^ and Haarlem (KNMI). Correlations (Fig. 1, circles) confirm the results from ERA5.

To analyse low-frequency variability, we formed seasonal averages and then smoothed these with a spline with a smoothing parameter of 0.57 or a locally estimated scatterplot smoothing regression with a window size of 42 years, both of which give almost undistinguishable results and correspond approximately to a 30-year moving average. We show the spline results in this paper.

### Stratospheric warmings and solar storms

To composite SSWs, we used a list of major warmings (defined as a reversal of the zonal mean zonal wind at 10 hPa at 60° N in ERA5) from Lu et al.^31^ based on ERA5, which we complemented backward to January 1940 and forward to December 2024 by adding three events (Supplementary Table 4). We also composited 176 minor warmings (defined as the first day of a minimum 5-day reversal of the temperature gradient at 10 hPa between 60° N and the North Pole in ERA5 that is not accompanied by a wind reversal). The results (Fig. 3) show no signal, as expected.

Lists of historical solar storms that could be used for compositing are not readily available. Several authors provide lists from different types of solar storms, which we combined (Supplementary Table 5). Evidence comes from reported low-latitude aurorae (back to the eighteenth century), geomagnetic storms (nineteenth century) or fast interplanetary shock events (fast-transit events, nineteenth century), all of which indicate potential EEP events. GLEs and SPEs were observed after the 1940s (neutron monitors) and after 1976 (satellites), respectively. In total, we used 102 storms in September–March (Supplementary Table 5). Note that near solar maximum years, storms often occur in short intervals. Although we require a 15-day distance between events, storm effects may overlap in the displayed 130-day time interval and smooth the signal. We also composited pre-1940 storms (*n* = 28), post-1940 storms (*n* = 74), potential EEP events (geomagnetic storms, low-latitude aurorae and fast-transit events, *n* = 67), GLEs (*n* = 30) and strong SPEs that are not GLEs (>1,000 proton flux units, *n* = 13).

## Online content

Any methods, additional references, Nature Portfolio reporting summaries, source data, extended data, supplementary information, acknowledgements, peer review information; details of author contributions and competing interests; and statements of data and code availability are available at https://doi.org/10.1038/s41561-026-02069-z.

## Supplementary information

Supplementary Information Supplementary Tables 1–8 and references.

Peer Review File

## Data Availability

The ModE-RA^18^ and ModE-Sim^40^ data are available via the DKRZ at https://www.wdc-climate.de/ui/entry?acronym=ModE-RA. ERA5 reanalysis data are available from the Copernicus Climate Change Service Data Store. Wind direction data are available from Mellado-Cano et al.^62^. Latitude and speed of the polar front jet and the subtropical jet are available from Rivoire et al.^23^. The quality-controlled and homogenized upressure series, the QC flags, the gap-filled series as well as the indices presented in this paper are available via Figshare at https://doi.org/10.6084/m9.figshare.29445245 (ref. ^63^). The daily pressure series from the ensemble of ModE-Sim are available form the same link. The monthly jet indices from ModE-RA are available via Figshare at https://doi.org/10.6084/m9.figshare.28001564 (ref. ^64^).

## Code availability

The R code used to homogenize the Berlin series is available via GitHub at https://github.com/ybrugnara/Berlin. Code to calculate the daily indices as well as code to calculate the composites (Fig. 3), including the lists of SSWs and solar storms, is available via Figshare at https://doi.org/10.6084/m9.figshare.29445245 (ref. ^63^). Code to calculate the monthly pressure indices from ModE-RA is available via Figshare at https://doi.org/10.6084/m9.figshare.28001564 (ref. ^64^).
