# Supplementary material for: A strengthening yet less extreme Atlantic–European jet during winter
Source: Nat Geosci. 2026 Sep 10;19(10):1182–9. doi: 10.1038/s41561-026-02069-z (PMC13645667; doi:10.1038/s41561-026-02069-z)
Supplement: Supplementary file 1 [file 41561_2026_2069_MOESM1_ESM.pdf]

nature geoscience

Supplementary information

https://doi.org/10.1038/s41561-026-02069-z

# A strengthening yet less extreme Atlantic–European jet during winter

In the format provided by the authors and unedited

## A strengthening yet less extreme Atlantic–European jet during winter

Stefan Brönnimann,[1,2] Yuri Brugnara,[1,2] and Patrick Kallabis[1,2]

[1] Institute of Geography, University of Bern

[2] Oeschger Centre for Climate Change Research, University of Bern

**Supplementary Table 1.** Segments of the Berlin pressure series.

| Observer | Period | Location | Reference |
|---|---|---|---|
| DWD | 1876-2023 | Berlin | |
| DWD | 1824-1940 | Jena | |
| František J. J. Kreybich | 1800-1818 | Žitenice | Brázdil et al. (2007)[72] |
| Beguelin, Achard (Palatine Society) | 1781-1788 | Berlin | Pappert et al. (2021)[73] |
| Karl Ludwig Gronau | 1775-1801 | Berlin | |
| Johann Heinrich Lambert | 1769-1777 | Berlin | |
| Carl August Brand | 1760-1794 | Berlin and Zeesen | |
| Johann Friedrich Weidler | 1728-1729 | Wittenberg | |
| Daniel Ernst Jablonski | 1725-1727 | Berlin | |
| Kirch family | 1721-1774 | Berlin | Brönnimann et al. (2023)[44] |
| Christian Trautmann | 1720-1730 | Löbau | Brázdil et al. (2008)[64] |

**Supplementary Table 2.** Coefficient of correlation between monthly averages of the jet indices and monthly wind indices (frequency of westerly, southerly, northerly or easterly winds) over the English Channel.

| **1725-2013** | **WI dd [%]** | **SI dd [%]** | **NI dd [%]** | **EI dd [%]** |
|---|---|---|---|---|
| Strength | 0.33 | 0.06 | -0.20 | -0.27 |
| Tilt | -0.01 | -0.41 | 0.35 | 0.07 |
| Latitude | -0.28 | 0.02 | -0.07 | 0.32 |
| **1725-1874** | **WI dd [%]** | **SI dd [%]** | **NI dd [%]** | **EI dd [%]** |
| Strength | 0.32 | 0.08 | -0.24 | -0.27 |
| Tilt | 0.04 | -0.37 | 0.34 | -0.02 |
| Latitude | -0.21 | -0.03 | -0.03 | 0.28 |
| **1725-2013 Nov.-Apr.** | **WI dd [%]** | **SI dd [%]** | **NI dd [%]** | **EI dd [%]** |
| Strength | 0.43 | 0.09 | -0.23 | -0.34 |
| Tilt | 0.05 | -0.46 | 0.39 | 0.01 |
| Latitude | -0.29 | 0.07 | -0.10 | 0.31 |

**Supplementary Table 3.** Most extreme index values for 1-, 5- and 15-day events since 1806 and central date of occurrence.

| **1 day** | | | **5 days** | | | **15 days** | | |
|---|---|---|---|---|---|---|---|---|
| **Strength** | **Tilt** | **Latitude** | **Strength** | **Tilt** | **Latitude** | **Strength** | **Tilt** | **Latitude** |
| 5.51 | 3.14 | 3.47 | 3.80 | 2.36 | 2.87 | 3.60 | 1.64 | 2.35 |
| -6.37 | -3.81 | -4.88 | -4.59 | -2.54 | -3.69 | -3.21 | -1.95 | -2.10 |
| 22 Jan 1993 | 5 Feb 1999 | 23 Jan 1907 | 8 Jan 2005 | 2 Feb 1922 | 16 Jan 1882 | 17 Jan 1993 | 8 Jan 2019 | 20 Jan 1882 |
| 26 Dec 1836 | 11 Nov 1891 | 26 Feb 1989 | 18 Oct 1835 | 23 Jan 1937 | 26 Feb 1889 | 28 Nov 1875 | 24 Jan 1937 | 24 Dec 1821 |

**Supplementary Table 4.** List of SSW events used in this study. We used the list of Lu and Rao[31] which is based on Charlton and Polvani[75], extended to the period October 1940 to December 2022 using ERA5. We added two earlier events in 1940 based on ERA5, consistent with reported SSWs in Brönnimann et al.[76], and one later event. Events described as "downward propagating" in Lu and Rao[31] are marked in bold.

| y | M | d | y | m | d | Y | m | d |
|---|---|---|---|---|---|---|---|---|
| 1940 | 1 | 11 | **1968** | **11** | **28** | 2000 | 3 | 20 |
| 1940 | 2 | 9 | 1969 | 3 | 13 | **2001** | **2** | **11** |
| **1941** | **2** | **7** | **1970** | **1** | **2** | 2001 | 12 | 30 |
| 1942 | 3 | 21 | **1971** | **1** | **18** | **2002** | **2** | **17** |
| 1945 | 3 | 19 | **1971** | **3** | **20** | 2003 | 1 | 18 |
| 1946 | 2 | 19 | 1973 | 1 | 31 | **2004** | **1** | **5** |
| 1946 | 3 | 19 | **1977** | **1** | **9** | **2006** | **1** | **21** |
| 1950 | 3 | 5 | **1979** | **2** | **22** | 2007 | 2 | 24 |
| **1952** | **2** | **22** | **1980** | **2** | **29** | **2008** | **2** | **22** |
| 1952 | 11 | 19 | 1981 | 3 | 4 | **2009** | **1** | **24** |
| **1954** | **12** | **18** | 1981 | 12 | 4 | **2010** | **2** | **9** |
| **1955** | **1** | **26** | **1984** | **2** | **24** | **2010** | **3** | **24** |
| **1957** | **2** | **4** | **1985** | **1** | **1** | **2013** | **1** | **6** |
| **1958** | **2** | **1** | **1987** | **1** | **23** | **2018** | **2** | **12** |
| **1960** | **1** | **17** | 1987 | 12 | 8 | **2019** | **1** | **1** |
| 1963 | 1 | 27 | 1988 | 3 | 14 | **2021** | **1** | **5** |
| 1965 | 12 | 16 | 1989 | 2 | 21 | 2023 | 2 | 16 |
| **1966** | **2** | **22** | 1998 | 12 | 15 | | | |
| **1968** | **1** | **7** | **1999** | **2** | **26** | | | |

**Supplementary Table 5.** List of historical solar storms between September and March used in this study. The selection largely follows the categorization of Cliver and Svalgaard.[77] If several events occurred within less than 15 days, the first one is chosen. Sources: A = Table 7 in Cliver et al. (2022)[78] and B = Table 4 in Usoskin et al. (2023)[79] for low-latitude aurorae, C = Table 2 in Lefèvre et al. (2016)[80] for geomagnetic storms (peak aa > 300 nT), D = Table 3 in Gopalswamy et al. (2005)[81] for fast transit events (<30 hours), E = Table 1 in Cliver et al. (2020)[82] and F = Table 1 in Usoskin et al. (2020)[83] for GLEs (statistically significant enhancements of count rates of at least two differently located neutron monitors), G = Table 7 in Hudson et al. (2024)[84] for solar soft-X-ray flares (class X10, i.e., $>10^{-3}$ W $m^{-2}$), H = NOAA Space Weather Prediction Center[85] for strong SPEs (>1000 pfu).

| Year | Mon | Day | Event | Source | Reference |
|---|---|---|---|---|---|
| 1730 | 2 | 15 | low-latitude aurora | A, B | Hayakawa et al. (2018)[86] |
| 1770 | 9 | 15 | low-latitude aurora | A, B | Hayakawa et al. (2017)[87] |
| 1788 | 10 | 21 | low-latitude aurora | B | Hattori et al. (2021)[88] |
| 1789 | 11 | 14 | low-latitude aurora | B | de la Paz Ramos-Lara et al. (2021)[89] |
| 1848 | 11 | 17 | low-latitude aurora | B | |
| 1859 | 9 | 1 | low-latitude aurora, geomagnetic storm, fast transit event, solar flare | A, B, D | Hayakawa et al. (2019, 2023)[65,66], Hudson et al. (2025)[67], Cliver and Dietrich (2013)[68] |
| 1870 | 9 | 24 | geomagnetic storm (aa index) | C | |
| 1870 | 10 | 24 | low-latitude aurora, geomagnetic storm (aa index) | B, C | Vaquero et al. (2008)[90] |
| 1871 | 2 | 12 | geomagnetic storm (aa index) | C | Hayakawa et al. (2019)[65] |
| 1872 | 2 | 4 | low-latitude aurora, geomagnetic storm | A, B | Hayakawa et al. (2019)[65] |
| 1872 | 10 | 14 | geomagnetic storm (aa index) | C | Hayakawa et al. (2019, 2023)[65,91] |
| 1881 | 1 | 31 | geomagnetic storm (aa index) | C | |
| 1882 | 11 | 17 | geomagnetic storm (aa index) | C | Love (2018)[92] |
| 1886 | 3 | 30 | geomagnetic storm (aa index) | C | |

| | | | | | |
|---|---|---|---|---|---|
| 1892 | 2 | 14 | geomagnetic storm (aa index) | C | |
| 1894 | 2 | 25 | geomagnetic storm (aa index) | C | |
| 1898 | 3 | 15 | geomagnetic storm (aa index) | C | |
| 1898 | 9 | 9 | geomagnetic storm (aa index) | C | |
| 1903 | 10 | 31 | geomagnetic storm (aa index) | A, C | Hayakawa et al. (2020)[93] |
| 1907 | 2 | 9 | geomagnetic storm (aa index) | C | |
| 1908 | 9 | 10 | geomagnetic storm (aa index), fast transit event | C, D | |
| 1909 | 9 | 24 | low-latitude aurora, geomagnetic storm (aa index), fast transit event | A, B, C, D | Hayakawa et al. (2019)[65] |
| 1916 | 1 | 10 | fast transit event | D | |
| 1917 | 2 | 14 | fast transit event | D | |
| 1920 | 3 | 23 | geomagnetic storm (aa index) | C | |
| 1926 | 1 | 25 | geomagnetic storm (aa index), fast transit event | C, D | |
| 1926 | 10 | 15 | geomagnetic storm (aa index) | C | |
| 1938 | 1 | 16 | low-latitude aurora, geomagnetic storm (aa index), fast transit event | B, C, D | Hayakawa et al. (2021a)[69] |
| 1940 | 3 | 24 | low-latitude aurora, geomagnetic storm (aa index) | B, C | Hayakawa et al. (2022)[94] |
| 1941 | 2 | 28 | low-latitude aurora, geomagnetic storm (aa index), fast transit event | A, C, D | Hayakawa et al. (2021b)[95] |
| 1941 | 9 | 17 | geomagnetic storm (aa index), fast transit event | C, D | |
| 1942 | 2 | 28 | geomagnetic storm (aa index), fast transit event, solar particle storm (GLE#1,2) | C, D, E | |
| 1944 | 12 | 16 | geomagnetic storm (aa index) | C | |
| 1946 | 2 | 6 | geomagnetic storm (aa index), fast transit event | C, D | |
| 1946 | 3 | 25 | low-latitude aurora, geomagnetic storm (aa index) | A, C | |
| 1946 | 9 | 22 | geomagnetic storm (aa index) | C | |
| 1948 | 10 | 17 | geomagnetic storm (aa index) | C | |
| 1949 | 1 | 25 | geomagnetic storm (aa index) | C | |
| 1949 | 11 | 19 | solar particle storm (GLE#4) | E | |
| 1951 | 10 | 28 | geomagnetic storm (aa index) | C | |
| 1956 | 2 | 23 | solar particle storm (GLE#5) | E, F | Hayakawa et al. (2024)[70] |
| 1957 | 1 | 20 | geomagnetic storm (aa index), fast transit event | C, D | |
| 1957 | 3 | 2 | geomagnetic storm (aa index) | C | |
| 1957 | 9 | 4 | geomagnetic storm (aa index) | C | |
| 1957 | 9 | 29 | geomagnetic storm (aa index) | C | |
| 1958 | 2 | 9 | low-latitude aurora, geomagnetic storm (aa index), fast transit event | B, C, D | |
| 1958 | 9 | 4 | geomagnetic storm (aa index) | C | |
| 1959 | 3 | 27 | geomagnetic storm (aa index) | C | |
| 1960 | 9 | 3 | solar particle storm (GLE#9) | E | |
| 1960 | 10 | 6 | geomagnetic storm (aa index) | C | |
| 1960 | 11 | 12 | geomagnetic storm (aa index), fast transit event, solar particle storm (GLE#10-12) | C, D, E, F | |
| 1961 | 10 | 28 | geomagnetic storm (aa index) | C | |
| 1963 | 9 | 22 | geomagnetic storm (aa index) | C | |
| 1967 | 1 | 28 | solar particle storm (GLE#16) | E, F | |
| 1967 | 2 | 16 | geomagnetic storm (aa index) | C | |
| 1968 | 9 | 29 | solar particle storm (GLE#18) | E, F | |
| 1968 | 11 | 18 | solar particle storm (GLE#19) | E, F | |
| 1969 | 2 | 2 | geomagnetic storm (aa index) | C | |
| 1969 | 2 | 25 | solar particle storm (GLE#20) | E, F | |
| 1969 | 3 | 30 | solar particle storm (GLE#21) | E, F | |
| 1970 | 3 | 8 | geomagnetic storm (aa index) | C | |
| 1971 | 1 | 24 | solar particle storm (GLE#22) | E, F | |
| 1971 | 9 | 1 | solar particle storm (GLE#23) | E, F | |
| 1977 | 9 | 19 | solar particle storm (GLE#28 and #29) | E, F | |
| 1977 | 11 | 22 | solar particle storm (GLE#30) | E, F | |
| 1978 | 9 | 23 | solar particle storm (GLE#32) | E, F, H | |
| 1981 | 10 | 12 | solar particle storm (GLE#36) | E, F, H | |
| 1982 | 9 | 6 | geomagnetic storm (aa index) | C | |

| | | | | | |
|---|---|---|---|---|---|
| 1982 | 11 | 26 | solar particle storm (GLE#37) | E, F | |
| 1982 | 12 | 17 | solar flare, solar particle storm (GLE#38, 8 Dec) | E, F, G, H | |
| 1984 | 2 | 16 | solar particle storm (GLE#39) | E, F | |
| 1986 | 2 | 8 | geomagnetic storm (aa index) | C | |
| 1989 | 3 | 6 | solar flare, strong SPE 8 Mar (and 17 Mar), low-latitude aurora, geomagnetic storm (aa index), 13 Mar | A, B, C, G, H | Boteler (2019)[96] |
| 1989 | 9 | 29 | solar particle storm (GLE#42) | E, F, H | |
| 1989 | 10 | 19 | geomagnetic storm (aa index), solar particle storm (GLE#43), solar flare | C, E, F, G, H | |
| 1989 | 11 | 15 | geomagnetic storm (aa index), solar particle storm (GLE#46) | C, E, F | |
| 1989 | 11 | 30 | strong SPE | H | |
| 1991 | 1 | 25 | solar flare | G | |
| 1991 | 3 | 24 | geomagnetic storm (aa index), strong SPE | C, H | |
| 1991 | 11 | 8 | geomagnetic storm (aa index) | C | |
| 1992 | 9 | 9 | geomagnetic storm (aa index) | C | |
| 1992 | 11 | 2 | solar particle storm (GLE#54) | E, H | |
| 1994 | 2 | 20 | strong SPE | H | |
| 1997 | 11 | 6 | solar particle storm (GLE#55) | E, F | |
| 1998 | 9 | 30 | strong SPE | H | |
| 2000 | 9 | 17 | geomagnetic storm (aa index) | C | |
| 2000 | 11 | 8 | strong SPE | H | |
| 2001 | 9 | 24 | strong SPE | H | |
| 2001 | 11 | 4 | geomagnetic storm (aa index), solar particle storm (GLE#62) | C, E, F, H | |
| 2001 | 11 | 24 | geomagnetic storm (aa index), strong SPE | C, H | |
| 2001 | 12 | 26 | solar particle storm (GLE#63) | E, F | |
| 2003 | 10 | 26 | geomagnetic storm (aa index), fast transit event, solar particle storm (GLE#65-67), solar flare | B, C, E, F, G, H | |
| 2003 | 11 | 20 | geomagnetic storm (aa index) | C | |
| 2004 | 11 | 8 | geomagnetic storm (aa index) | C | |
| 2005 | 1 | 17 | solar particle storm (GLE#68, 69) | E, F, H | |
| 2005 | 9 | 7 | solar flare, strong SPE | G, H | |
| 2006 | 12 | 13 | solar particle storm (GLE#70) | E, F, H | |
| 2012 | 1 | 23 | strong SPE | H | |
| 2012 | 3 | 7 | strong SPE | H | |
| 2014 | 1 | 6 | strong SPE | H | |
| 2017 | 9 | 6 | solar particle storm (GLE#72), solar flare | E, F, G | Zhu et al. (2021)[97] |
| 2024 | 10 | 11 | geomagnetic storm, strong SPE | H | Oliveira et al. (2025)[98] |

**Supplementary Table 6.** CMIP6 simulations used in this study. Model and simulation, institution, resolution and number of levels, and reference (° = SSP2-4.5, + = SSP3-7.0, * = SSP5-8.5; all others could be used for all scenarios).

| Nr | Model Name | Institution / Consortium | Nominal Resolution and Levels | Source |
|---|---|---|---|---|
| 1 | ACCESS-CM2 | CSIRO and Australia Research Council Centre of Excellence for Climate System Science (ARCCSS) Australia | 250 km, 85 L | Dix et al. (2019)[989] |
| 2 | ACCESS-ESM1-5°* | CSIRO and Australia Research Council Centre of Excellence for Climate System Science (ARCCSS) Australia | 250 km, 38 L | Ziehn et al. (2019)[100] |
| 3 | AWI-ESM-1-1-LR | Alfred Wegener Institut (AWI) Germany | 250 km, 47 L | Danek et al. (2020)[101] |
| 4 | BCC-CSM2-MR | Beijing Climate Centre (BCC), China | 100 km, 46 L | Xin et al. (2018)[102] |
| 5 | BCC-ESM1 | Beijing Climate Centre (BCC), China | 250 km, 26 L | Zhang et al. (2018)[103] |

| | | | | |
|---|---|---|---|---|
| 6 | CESM2* | National Center for Atmospheric Research (NCAR), USA | 100 km, 32 L | Danabasoglu (2019a)[104] |
| 7 | CESM2-WACCM | National Center for Atmospheric Research (NCAR), USA | 100 km, 70 L | Danabasoglu (2019b)[105] |
| 8 | CESM2-WACCM-FV2 | National Center for Atmospheric Research (NCAR), USA | 250 km, 70 L | Danabasoglu (2019c)[106] |
| 9 | CMCC-CM2-HR4* | Centro Euro-Mediterraneo sui Cambiamenti Climatici (CMCC), Italy | 100 km, 26 L | Scoccimarro et al. (2020)[107] |
| 10 | CMCC-CM2-SR5 | Centro Euro-Mediterraneo sui Cambiamenti Climatici (CMCC), Italy | 100 km, 30 L | Lovato and Peano (2020)[108] |
| 11 | CNRM-CM6-1-HR+* | Centre National de Recherches Meteorologiques (CNRM) and Centre European de Recherche et de Formation Avance en Calcul Scientifique (CERFACS), France | 100 km, 91 L | Voldoire (2019)[109] |
| 12 | EC-Earth3-AerChem | EC-Earth Consortium, Europe | 100 km, 91 L | EC-Earth Consortium (EC-Earth)(2019a)[110] |
| 13 | EC-Earth3-CC+* | EC-Earth Consortium, Europe | 100 km, 91 L | EC-Earth Consortium (EC-Earth)(2019b)[111] |
| 14 | EC-Earth3-Veg-LR° | EC-Earth Consortium, Europe | 250 km, 62 L | EC-Earth Consortium (EC-Earth)(2019c)[112] |
| 15 | FGOALS-f3-L | CAS (Chinese Academy of Sciences), China | 100 km, 32 L | Yu et al. (2018)[113] |
| 16 | FGOALS-g3 | CAS (Chinese Academy of Sciences), China | 250 km, 26 L | Li et al. (2019)[114] |
| 17 | GFDL-ESM4 | National Oceanic and Atmospheric Administration (NOAA), Geophysical Fluid Dynamics Laboratory (GFDL), USA | 100 km, 49 L | Krasting et al. (2018)[115] |
| 18 | HadGEM3-GC31-LL | Met Office Hadley Centre MOHC, UK | 250 km, 85 L | Ridley et al. (2018)[116] |
| 19 | HadGEM3-GC31-MM | Met Office Hadley Centre MOHC, UK | 100 km, 85 L | Ridley et al. (2019)[117] |
| 20 | KACE-1-0-G* | National Institute of Meteorological Sciences (NIMS), Korea Meteorological Administration (KMA), Republic of Korea | 250 km, 85 L | Byun et al. (2019)[118] |
| 21 | INM-CM4-8+° | Institute for Numerical Mathematics(INM), Russian Federation | 100 km, 21 L | Volodin et al. (2019)[119] |
| 22 | MIROC6+* | MIROC Consortium JAMSTEC, AORINIES, R-CCS Japan | 250 km, 81 L | Tatebe et al. (2018)[120] |
| 23 | MIROC-ES2L | MIROC Consortium JAMSTEC, AORINIES, R-CCS Japan | 500 km, 40 L | Hajima et al. (2019)[121] |
| 24 | MPI-ESM1-2-HR+° | Max Planck Institute for Meteorology (MPI-M), Germany | 100 km 95 L | Jungclaus et al. (2019)[122] |
| 25 | MPI-ESM1-2-LR | Max Planck Institute for Meteorology (MPI-M), Germany | 250 km, 47 L | Wieners et al. (2019)[123] |
| 26 | MRI-ESM2-0 | Meteorological Research Institute(MRI), Japan | 100 km, 80 L | Yukimoto et al. (2019)[124] |
| 27 | NorESM2-MM* | NorESM Climate Modelling Consortium (NCC), Norway | 100 km, 32 L | Bentsen et al. (2019)[125] |

| | | | | |
|---|---|---|---|---|
| 28 | SAM0-UNICON | Seoul National University (SNU), Republic of Korea | 100 km 30 L | Park et al. (2019)[165] |
| 29 | TaiESM1 | Academia Sinica Research Center for Environmental Changes (AS-RCEC), Taiwan, China | 100 km, 30 L | Lee and Liang (2019)[127] |

**Supplementary Table 7.** Breakpoints corrected in the Berlin pressure series. The reference series are used to correct the period preceding the breakpoint.

| Date | Segment | Metadata | Reference series |
|---|---|---|---|
| 2012-10-01 | Dahlem | | Tempelhof, Magdeburg, Potsdam |
| 1997-08-01 | Dahlem | New barometer type | Tempelhof, Leipzig, Magdeburg |
| 1950-01-01 | Dahlem | Relocation | Dahlem, Tempelhof |
| 1908-01-01 | Dahlem | | Potsdam, Görlitz, Jena, Magdeburg, Waren |
| 1901-01-01 | Dahlem | | Jena, Leipzig, Magdeburg |
| 1887-01-01 | Dahlem | | Jena, Leipzig, Magdeburg |
| 1883-01-01 | Dahlem | | Jena, Leipzig |
| 1878-07-01 | Jena | Data gap | De Bilt, Gdansk, Leipzig, Prague |
| 1838-01-01 | Jena | | De Bilt, Gdansk, Karlsruhe, Lund, Prague |
| 1830-07-01 | Jena | | De Bilt, Gdansk, Karlsruhe, Lund, Prague |
| 1794-05-01 | Gronau | | [Too short period (data deleted)] |
| 1794-02-01 | Gronau | | Lund, Prague |
| 1785-07-23 | Gronau | Data gap | Berlin (Palatine), Copenhagen, Erfurt, Mannheim, Zagan |
| 1783-10-01 | Gronau | | Berlin (Palatine), Copenhagen, Erfurt, Mannheim, Zagan |
| 1783-01-01 | Gronau | | Berlin (Palatine), Copenhagen, Erfurt, Mannheim, Zagan |
| 1780-08-01 | Gronau | | Basel, De Bilt, Franeker |
| 1785-01-01 | Brand | | Berlin (Palatine), Erfurt, Zagan, Mannheim, Copenhagen |
| 1780-08-01 | Brand | | Basel, De Bilt, Vienna |
| 1773-08-01 | Brand | | Basel, De Bilt, Padova |
| 1775-11-01 | Lambert | | London, Paris, Vienna |
| 1765-09-01 | Kirch | Relocation | London, Padova, Paris |
| 1761-04-01 | Kirch | | [Too short period (data deleted)] |
| 1760-10-22 | Kirch | | London, Padova, Paris |
| 1727-07-01 | Löbau | | London, Padova, Uppsala |
| 1722-01-01 | Kirch | | London |

**Supplementary Table 8.** Gap filling of pressure series. r = correlation of the gap-filling with the analog-EKF approach.

| Station | Gaps in raw series | After filling with neighbouring series | After filling with ERA5/20CRv3 | After filling with analogEKF | R |
|---|---|---|---|---|---|
| Padova | 0.88% | 0.46% | 0.015% | 0.000% | 0.85 |
| Uppsala | 0.98% | 0.42% | 0.006% | 0.000% | 0.56 |
| London | 0.48% | 0.48% | 0.071% | 0.000% | 0.96 |
| Berlin | 2.90% | 2.90% | 1.820% | 0.000% | 0.91 |

## Supplementary References


72 Brázdil, R., Řezníčková, L. and Valášek, H. Early instrumental meteorological observations in the Czech Lands III: František Jindřich Jakub Kreybich, Žitenice, 1787-1829. *Meteorologický časopis* **10(2),** 63-74 (2007).

73 Pappert, D., Brugnara, Y., Jourdain, S., Pospieszyńska, A., Przybylak, R., Rohr, C. & Brönnimann, S. Unlocking weather observations from the Societas Meteorologica Palatina (1781–1792). *Clim. Past* **17,** 2361–2379 (2021), https://doi.org/10.5194/cp-17-2361-2021

74 Brázdil, R., Kiss, A., Luterbacher, J. & Valášek, H. Weather patterns in eastern Slovakia 1717–1730, based on records from the Breslau meteorological network. *Int. J. Climatol.* **28,** 1639-1651 (2008), https://doi.org/10.1002/joc.1667

75 Charlton, A. J. & Polvani, L. M. A New Look at Stratospheric Sudden Warmings. Part I: Climatology and Modeling Benchmarks. *J. Clim.* **20,** 449–469 (2007), https://doi.org/10.1175/JCLI3996.1.

76 Brönnimann, S., Luterbacher, J., Staehelin J. & Svendby, T. M. An extreme anomaly in stratospheric ozone over Europe in 1940–1942. *Geophys. Res. Lett.* **31,** L08101 (2004), https://doi.org/10.1029/2004GL019611

77 Cliver, E. W. & Svalgaard, L. The 1859 Solar–Terrestrial Disturbance And the Current Limits of Extreme Space Weather Activity. *Sol. Phys*. **224,** 407–422 (2004), https://doi.org/10.1007/s11207-005-4980-z

78 Cliver, E. W., Schrijver, C. J., Shibata, K. & Usoskin, I. G. Extreme solar events. Living. *Rev. Sol. Phys.* **19,** 2 (2022). https://doi.org/10.1007/s41116-022-00033-8

79 Usoskin, I. et al. Extreme Solar Events: Setting up a Paradigm. *Space Sci. Rev.* **219,** 73 (2023). https://doi.org/10.1007/s11214-023-01018-1

80 Lefèvre, L. et al. Detailed Analysis of Solar Data Related to Historical Extreme Geomagnetic Storms: 1868 – 2010. *Sol. Phys.* **291,** 1483–1531 (2016), https://doi.org/10.1007/s11207-016-0892-3

81 Gopalswamy, N. et al. Coronal mass ejections and other extreme characteristics of the 2003 October–November solar eruptions. *J. Geophys. Res.* **110,** A09S15 (2005), doi:10.1029/2004JA010958.

82 Cliver, E. W., Hayakawa, H., Love, J. J. & Neidig, D. F. On the Size of the Flare Associated with the Solar Proton Event in 774 AD. *ApJ* **903,** 41 (2020).

83 Usoskin, I., Koldobskiy, S., Kovaltsov, G. A., Gil, A., Usoskina, I., Willamo, T. & Ibragimov, A. Revised GLE database: Fluences of solar energetic particles as measured by the neutron-monitor network since 1956. *A&A* **640,** A17 (2020)

84 Hudson, H. et al. The Greatest GOES Soft X-ray Flares: Saturation and Recalibration over Two Hale Cycles. *Sol. Phys.* **299,** 39 (2024), https://doi.org/10.1007/s11207-024-02287-x

85 NOAA Space Weather Prediction Center. Solar Proton Events Affecting the Earth Environment (last updated 12 Nov 2025), ftp://ftp.swpc.noaa.gov/pub/indices/SPE.txt

86 Hayakawa, H. et al. A great space weather event in February 1730. *A&A* **616,** A177 (2018), https://doi.org/10.1051/0004-6361/201832735

87 Hayakawa, H. et al. Long-lasting Extreme Magnetic Storm Activities in 1770 Found in Historical Documents. *ApJL* **850,** L31 (2017), https://doi.org/10.3847/2041-8213/aa9661

88 Hattori, K., Hayakawa, H. & Ebihara, Y. The extreme space weather events in October 1788. *Publ. Astron. Soc. Jpn.* **73,** 1367–1374 (2021), https://doi.org/10.1093/pasj/psab079

89 de la Paz Ramos-Lara, M., Durand-Manterola, H. J. & Adrián Canales-Pozos, S. The low latitude aurora borealis of 1789 from Mexico records. *Adv. Space Res.* **68,** 320-2331 (2021), https://doi.org/10.1016/j.asr.2021.05.001.

90 Vaquero, J. M., Valente, M. A., Trigo, R. M., Ribeiro, P. & Gallego, M. C. The 1870 space weather event: Geomagnetic and auroral records. *J. Geophys. Res.* **113,** A08230 (2008), https://doi.org/10.1029/2007JA012943.

91 Hayakawa, H. et al. The Extreme Space Weather Event of 1872 February: Sunspots, Magnetic Disturbance, and Auroral Displays. ApJ 959, 23 (2023), https://doi.org/10.3847/1538-4357/acc6cc

92 Love, J. J. The electric storm of November 1882. *Space Weather* **16,** 37–46 (2018), https://doi.org/10.1002/2017SW001795

93 Hayakawa, H. et al. The Extreme Space Weather Event in 1903 October/November: An Outburst from the Quiet Sun. *ApJL* **897,** L10 (2020).

94 Hayakawa, H. et al. The extreme solar and geomagnetic storms on 1940 March 20–25. *Mon. Not. R. Astron. Soc.* **517,** 1709–1723 (2022), https://doi.org/10.1093/mnras/stab3615

95 Hayakawa, H., Blake, S. P., Bhaskar, A., Hattori, K., Oliveira, D. M. & Ebihara, Y. The Extreme Space Weather Event in 1941 February/March. *ApJ* **908,** 209 (2021), https://doi.org/10.3847/1538-4357/abb772

96 Boteler, D. H. A 21st century view of the March 1989 magnetic storm. *Space Wea.* **17,** 1427–1441 (2019), https://doi.org/10.1029/2019SW002278

97 Zhu, B., Liu, Y. D., Kwon, R.-Y, Jin, M., Lee, L. C. & Xu, X. Shock Properties and Associated Characteristics of Solar Energetic Particles in the 2017 September 10 Ground-level Enhancement Event. *ApJ,* **921,** 26 (2021).

98 Oliveira, D. M., Zesta, E. & Nandy, D. The 10 October 2024 geomagnetic storm may have caused the premature reentry of a Starlink satellite. *Front. Astron. Space Sci.* **11,** 1522139 (2025), https://doi.org/10.3389/fspas.2024.1522139

99 Dix, M., et al. CSIRO-ARCCSS ACCESS-CM2 model output prepared for CMIP6 CMIP. Version 20240322. Earth System Grid Federation, https://doi.org/10.22033/ESGF/CMIP6.2281 (2019).

100 Ziehn, T., et al. CSIRO ACCESS-ESM1.5 model output prepared for CMIP6 CMIP. Version 20240322. Earth System Grid Federation, https://doi.org/10.22033/ESGF/CMIP6.2288 (2019).

101 Danek, C., et al. AWI AWI-ESM1.1LR model output prepared for CMIP6 CMIP. Version 20240322. Earth System Grid Federation, https://doi.org/10.22033/ESGF/CMIP6.9301 (2020).

102 Xin, X., et al. BCC BCC-CSM2MR model output prepared for CMIP6 CMIP. Version 20240322. Earth System Grid Federation, https://doi.org/10.22033/ESGF/CMIP6.1725 (2018).

103 Zhang, J., et al. BCC BCC-ESM1 model output prepared for CMIP6 CMIP. Version 20240322. Earth System Grid Federation, https://doi.org/10.22033/ESGF/CMIP6.1734 (2018).

104 Danabasoglu, G. NCAR CESM2 model output prepared for CMIP6 CMIP. Version 20240322. Earth System Grid Federation, https://doi.org/10.22033/ESGF/CMIP6.2185 (2019a).

105 Danabasoglu, G. NCAR CESM2-WACCM model output prepared for CMIP6 CMIP. Version 20240322. Earth System Grid Federation, https://doi.org/10.22033/ESGF/CMIP6.10024 (2019b).

106 Danabasoglu, G. NCAR CESM2-WACCM-FV2 model output prepared for CMIP6 CMIP. Version 20240322. Earth System Grid Federation, https://doi.org/10.22033/ESGF/CMIP6.11282 (2019c).

107 Scoccimarro, E., Bellucci, A. & Peano, D. CMCC CMCC-CM2-HR4 model output prepared for CMIP6 CMIP. Version 20240322. Earth System Grid Federation, https://doi.org/10.22033/ESGF/CMIP6.1358 (2019).

108 Lovato, T. & Peano, D. CMCC CMCC-CM2-SR5 model output prepared for CMIP6 CMIP. Version 20240322. Earth System Grid Federation, https://doi.org/10.22033/ESGF/CMIP6.1362 (2019).

109 Voldoire, A. CNRM-CERFACS CNRM-CM6-1-HR model output prepared for CMIP6 CMIP. Version 20240322. Earth System Grid Federation, https://doi.org/10.22033/ESGF/CMIP6.1385 (2019).

110 EC-Earth Consortium (EC-Earth). EC-Earth-Consortium EC-Earth3-AerChem model output prepared for CMIP6 CMIP. Version 20240322. Earth System Grid Federation, https://doi.org/10.22033/ESGF/CMIP6.639 (2019a).

111 EC-Earth Consortium (EC-Earth). EC-Earth-Consortium EC-Earth-3-CC model output prepared for CMIP6 CMIP. Version 20240322. Earth System Grid Federation, https://doi.org/10.22033/ESGF/CMIP6.640 (2019b).

112 EC-Earth Consortium (EC-Earth). EC-Earth-Consortium EC-Earth3-Veg-LR model output prepared for CMIP6 CMIP. Version 20240322. Earth System Grid Federation, https://doi.org/10.22033/ESGF/CMIP6.643 (2019c).

113 Yu, Y. CAS FGOALS-f3-L model output prepared for CMIP6 CMIP. Version 20240322. Earth System Grid Federation, https://doi.org/10.22033/ESGF/CMIP6.1782 (2019).

114 Li, L. CAS FGOALS-g3 model output prepared for CMIP6 CMIP. Version 20240322. Earth System Grid Federation, https://doi.org/10.22033/ESGF/CMIP6.1783 (2019).

115 Krasting, J. P., et al. NOAA-GFDL GFDL-ESM4 model output prepared for CMIP6 CMIP. Version 20240322. Earth System Grid Federation, https://doi.org/10.22033/ESGF/CMIP6.1407 (2018).

116 Ridley, J., Menary, M., Kuhlbrodt, T., Andrews, M. & Andrews, T. MOHC HadGEM3-GC31-LL model output prepared for CMIP6 CMIP. Version 20240322. Earth System Grid Federation, https://doi.org/10.22033/ESGF/CMIP6.419 (2019a).

117 Ridley, J., Menary, M., Kuhlbrodt, T., Andrews, M. & Andrews, T. MOHC HadGEM3-GC31-MM model output prepared for CMIP6 CMIP. Version 20240322. Earth System Grid Federation, https://doi.org/10.22033/ESGF/CMIP6.420 (2019b).

118 Byun, Y., et al. NIMS-KMA KACE1.0-G model output prepared for CMIP6 CMIP. Version 20240322. Earth System Grid Federation, https://doi.org/10.22033/ESGF/CMIP6.2241 (2019).

119 Volodin, E., et al. INM INM-CM4-8 model output prepared for CMIP6 CMIP. Version 20240322. Earth System Grid Federation, https://doi.org/10.22033/ESGF/CMIP6.1422 (2019).

120 Tatebe, H. & Watanabe, M. MIROC MIROC6 model output prepared for CMIP6 CMIP. Version 20240322. Earth System Grid Federation, https://doi.org/10.22033/ESGF/CMIP6.881 (2018).

121 Hajima, T., et al. MIROC MIROC6 model output prepared for CMIP6 CMIP. Version 20240322. Earth System Grid Federation, https://doi.org/10.22033/ESGF/CMIP6.902 (2018).

122 Jungclaus, J., et al. MPI-M MPIESM1.2-HR model output prepared for CMIP6 CMIP. Version 20240322. Earth System Grid Federation, https://doi.org/10.22033/ESGF/CMIP6.741 (2019).

123 Wieners, K. H. et al. MPI-M MPIESM1.2-LR model output prepared for CMIP6 CMIP. Version 20240322. Earth System Grid Federation, https://doi.org/10.22033/ESGF/CMIP6.742 (2019).

124 Yukimoto, S., et al. MRI MRI-ESM2.0 model output prepared for CMIP6 CMIP. Version 20240322. Earth System Grid Federation, https://doi.org/10.22033/ESGF/CMIP6.621 (2019).

125 Bentsen, M., et al. NCC NorESM2-MM model output prepared for CMIP6 CMIP. Version 20240322. Earth System Grid Federation, https://doi.org/10.22033/ESGF/CMIP6.506 (2019).

126 Park, S. & Shin, J. SNU SAM0-UNICON model output prepared for CMIP6 CMIP. Version 20240322. Earth System Grid Federation, https://doi.org/10.22033/ESGF/CMIP6.1489 (2019).

127 Lee, W. L. & Liang, H. C. AS-RCEC TaiESM1.0 model output prepared for CMIP6 CMIP. Version 20240322. Earth System Grid Federation, https://doi.org/10.22033/ESGF/CMIP6.9684 (2019).
