# Supplementary material for: A strengthening yet less extreme Atlantic–European jet during winter
Source: Nat Geosci. 2026 Sep 10;19(10):1182–9. doi: 10.1038/s41561-026-02069-z (PMC13645667; doi:10.1038/s41561-026-02069-z)
Supplement: Supplementary file 2 [file 41561_2026_2069_MOESM2_ESM.pdf]

nature portfolio

Peer Review File

A strengthening yet less extreme Atlantic European jet during winter

Corresponding Author: Professor Stefan Bronnimann

Version 0:

Reviewer comments:

Reviewer #1

(Remarks to the Author)
Summary

This is an interesting article that reconstructed the weather pattern in a high time resolution (including brand new datasets from Berlin) and assessed how solar storms in the past impacted the terrestrial weather. There were numerous studies who tried to model such interactions. However, these attempts have been in want of the data to either validate or criticize their models. The authors' study offers excellent data to critically reconsider such modeling efforts. This is ultimately worth publication, while there are several critical issues to be modified. I hope these comments are of some use.

:: Major Comments

(1) The term of "solar or geomagnetic storms" are a wide umbrella term. The authors explain that the solar storms affect nitrogen chemistry of the polar mesosphere and cause ozone destruction and strengthen the polar vortex. The authors need to clarify which aspects of the solar storms trigger such mechanisms. What do they mean here: solar flares, ground level enhancements (solar particle storms), geomagnetic storms, or everything? Phenomenologically, they are all different from one another. Perhaps in the appendix, the authors should explain when EEP get enhanced, citing not only Seppälä et al. (2025) but also DOI: 10.1038/ncomms6197, contextualizing solar flare, GLEs, and geomagnetic storms.

(2) The Carrington storm is known the "strongest" as a solar flare (DOI: 10.3847/2041-8213/acd853; DOI: 10.1093/mnras/staf967) and a geomagnetic storm (DOI: 10.1051/swsc/2013053; DOI: 10.3847/1538-4357/ac2601), while the SEP signal is robustly unconfirmed (DOI: 10.1051/swsc/2023030; DOI: 10.1007/s41116-023-00036-z). If the authors wish to raise the Carrington storm as the strongest solar particle storm in our period, they may wish to cite DOI:10.1029/2023GL106632 for a potential 14C signal detection. However, I personally know that the ice core data have shown a negative result and their interpretation will be rejected in the nearest future. The authors should clarify these different aspects of the Carrington storm, citing the said articles.

(3) The geographical classifications need some elaborations. Not too many readers would take Berlin as a representative of Eastern Europe. How do the authors define the geographical sector in Europe? How can these classifications get justified? They need some additional explanations.

(4) The authors indicate that "extreme excursions of the jet were always associated with the extreme weather" in the abstract. It will make their statement more convincing if they list such extreme excursions above certain thresholds that are justified with proper references and pair all such extreme weather tp each case. This can be an additional supplementary table.

(5) Table S5 is crucial for their analyses for the impact of the solar storm to the terrestrial impacts. We need to be extra careful for this table, as this is one of the key datasets for this article.

Firstly, the authors must not rely on Wikipedia for such events. The authors need to rely on refereed articles or at least some meaningful datasets for their analyses.

While Cliver and Svalgaard (2004) have been a benchmark, their results have been somewhat outdated owing to the research development in the last two decades. The authors are selecting these events for solar flares, fast-transit events, low-latitude aurorae, geomagnetic storms, and solar particle storms. Ideally, the authors should clarify thresholds for each of these aspects and generate a homogenized dataset. However, I know it takes ages.

For the time being, I would suggest the authors to use the following catalogs.

:: solar flares -- Table 7 of DOI: 10.1007/s11207-024-02287-x
:: fast-transit events -- Table 5 of DOI: 10.1007/s41116-022-00033-8
:: solar particle storms -- Table 1 of DOI: 10.3847/1538-4357/abad93 and Table 1 of DOI: 10.1051/0004-6361/202038272
:: geomagnetic storms -- Table 2 of DOI:10.1007/s11207-016-0892-3 for the aa index and Table 7 of DOI: 10.1007/s41116-022-00033-8
:: low-latitude aurorae -- Figure 17 of DOI: 10.3847/1538-4357/ad9335 and Table 4 of DOI: 10.1007/s11214-023-01018-1

For the latest storms, the author should cite review articles such as DOI:10.3847/1538-4357/ad9335 for the May 2024 storm and DOI: 10.3389/fspas.2024.1522139 for the Oct 2024 storm.

:: Minor Comments

Page 2 Paragraph 1: What do the authors mean the reanalysis data? Do they mean the official data from the modern authorities? They should elaborate the explanation here.

Some of the quotes to historical records are difficult to trace. The authors should elaborate their referencing to the Portland Guardian (with the release date and page number) and the Monthly Weather Review (volume and pages).

Page 6 Paragraph 4: The authors need to clarify how they selected the sudden

Page 7 Paragraph 1: Are the authors surveying the storms between September and March, as the CMIP6 model project a strengthening and narrowing of the Northern Atlantic jet in winter? It would be nice to clarify the reason of this selection.

(Remarks on code availability)

Reviewer #2

(Remarks to the Author)
Review of "Trends, Extremes and Imprint of Solar Storms in Daily Atlantic-European Jet Variability, 1725-2100" bx Bronnimann et al.

S. Bronnimann et al. define a new index that describe the characteristics (strength, tilt, and latitude) of the jet stream over the Atlantic-European region. The index is based on existing and newly made available datasets (four deseasonalized and standardized sets) from different locations over western Europe.

The authors study observation based daily jet stream variations since 1725 and compare it with climate model simulations to investigate past and future trends. The authors corelate their indices with climate data and investigate the influence of the jet stream on weather extremes, the effect of solar and geomagnetic activity on the jet stream parameters, and try to predict future variability of the jet.

Their results show that weather extremes over western Europe were always corelated with excursions of the jet stream. Their indices are able to explain a large part of day-day temperature variability and imprints of sudden stratospheric warmings due to solar induced geomagnetic storms were found (although considered not significant).
Not being an expert in this field, I find their study very interesting and worth being published. The results seem quite robust but I honestly think that the manuscript needs to be reviewed by other climate modelers or atmospheric scientists.
While I support publication of their results with some minor revisions, I focus my review on the construction of the observational dataset and a few general points.

In general, the manuscript often uses unexplained acronyms, which seem to be obvious in the climate modeller community but may not be so for the broad readership of Nature. So please go through the manuscript and check e.g., explain CMIP6; 20CRv3, SLP, etc.

Methods:
P 11: Data was aggregated to daily means – if the data was non evenly distributed, how was this done? Using interpolation or weighted averages?

Outlier test: series of tests were conducted to detect outliers (which tests?), why did the authors choose asymmetric exclusion criteria ? -5/+4 SD? "Most outliers were kept= - can you say how many % were discarded/kept?

What exactly does "homogenized data" mean?, please say very briefly

For the non-expert reader – please give a hint what Craddock and Pettit tests are; same for Wilcoxon test?

“breakpoints were detected using Craddock test with the support of some metadata” not easy to understand what was done - which metadata was used?

Neuchatel was detrended – which trend was applied? hPa/month?

“all series were deseasonalized and standardised” just briefly state what was done - ...by doing “what”...?

“data gaps were filled with reanalysis data” how was this done exactly?

“For filling these gaps, we used an analog method augmented with an offline Ensemble Kalman Filter approach” please give at least a reference for that is it the same as an “an off-line Kalman Filter”? that appears in the line below?

(Remarks on code availability)

Reviewer #3

(Remarks to the Author)
Review of Brönnimanne et al.

The manuscript by Brönnimanne et al. addresses a timely and highly relevant topic. Discrepancies between circulation and simulated trends are mounting, casting doubt on projections. One of the big issues is the relatively short observational record, and this is precisely the issue tackled in this paper.

The authors use daily sea-level pressure (SLP) observations, including a newly digitised Berlin time series, as a fingerprint of the large-scale flow often referred to as the “jet.” From these data, they construct indices characterising jet strength, latitude, and tilt. Overall, I find the paper to be original, well-crafted, and a valuable contribution to the literature. I have, however, a few concerns that I encourage the authors to consider.
Major points

My main concern is primarily terminological. The analysis is framed around “the jet”, and the conclusions refer to it. However, the indices employed are defined using continental SLP data and are therefore most directly linked to continental circulation patterns, including westerly flow over Europe.

In winter, the North Atlantic jet stream is a well-defined dynamical structure, especially over the ocean and western Europe. In summer, however, the notion of a coherent, persistent jet is more ambiguous. The appearance of a well-defined jet in summer largely arises from temporal averaging, whereas the instantaneous flow is better described as a succession of short-lived, spatially varying “jetlets” (e.g., Gammon, 2001). In this context, I wonder whether what the authors refer to as the jet—especially in summer—would be more accurately described as continental westerly winds.
Related to this point, “classical” definitions of the North Atlantic jet latitude and speed (e.g., Woollings et al., 2010) have been shown to be strongly correlated with the NAO. In contrast, the indices presented here exhibit only weak correlations with the NAO, further suggesting that they may be capturing a somewhat different aspect of the circulation.
These concerns do not invalidate the authors’ results, which I find robust and relevant. Rather, they pertain to interpretation and framing. It may be more appropriate to refer explicitly to “westerly winds” or “continental westerly flow,” and then demonstrate how the proposed indices relate to more classical jet-stream diagnostics during the period with sufficient data coverage (e.g., from 1950 onward). Such a clarification would, in my view, strengthen the paper and avoid potential conceptual ambiguity.

Minor comments:

“In fact, the observed strengthening is greater than in any one of the CMIP6 simulations”. See also Patterson and O’Reilly (2025)

“Given the large underestimation of the strengthening by CMIP6 models in the historical period, there is little confidence in the projected future trends”. See also Ossó et al., (2025)

“Two-year moving time series of lag 1 autocorrelation and of the variance do not reveal obvious changes (Fig. S1).” I believe the authors refer here to Fig. S2.

References
Patterson, M., & O’Reilly, C. H. (2025). Climate models struggle to simulate observed North Pacific jet trends, even accounting for tropical Pacific sea surface temperature trends. Geophysical Research Letters, 52, e2024GL113561. https://doi.org/10.1029/2024GL113561

Nielsen-Gammon JW. A Visualization of the Global Dynamic Tropopause. Bull Am Meteorol Soc. 2001;82(6):1151–67. https:// doi. org/ 10. 1175/ 1520- 0477(2001) 082>1151:AVOTGD<2.3.CO;2.

Ossó, A., Bladé, I., Karpechko, A. et al. Advancing Our Understanding of Eddy-driven Jet Stream Responses to Climate

Change – A Roadmap. Curr Clim Change Rep 11, 2 (2025). https://doi.org/10.1007/s40641-024-00199-3

(Remarks on code availability)

Version 1:

Reviewer comments:

Reviewer #1

(Remarks to the Author)
The authors have reasonably improved their manuscript. Still, their omissions though the revision process seem too radical. It is definitely better to pay more attention to the individual solar storms. I therefore have to suggest two major improvements to this manuscript.

(1) Their case studies on the weather response

This is particularly the case for their omission of the Carrington storm. It is definitely better and convincing to show the authors some concrete examples, apart from their statistical analyses.

The authors should revive their figure for the Carrington storm. Here, the authors need to describe this event as having the greatest flare magnitude (DOI: 10.3847/2041-8213/acd853; DOI: 10.1093/mnras/staf967) and the geomagnetic disturbance (DOI: 10.1051/swsc/2013053; DOI: 10.3847/1538-4357/ac2601) within the authors' catalog but lacking an evidence as the greatest particle storm (DOI: 10.1051/swsc/2023030).

The authors should add such figures for the greatest solar storms in the other categories (the greatest GLE and the fastest-transit event). For the greatest GLE, the authors should show their case analyses on GLE #5 on 23 Feb 1956 (DOI: 10.1051/0004-6361/202348699). For the fastest-transit event, the authors should show their case analyses on the solar storm on 4 Aug 1972 (DOI: 10.1029/2018SW002024; DOI: 10.3847/1538-4357/acf9fd).

(2) Table S5

Besides, the authors need to be mindful for their catalog. The authors need to explain that the table follows Cliver and Svalgaard's event category.

While I suggested to use these catalogs, we definitely need to consult event studies and avoid any unwanted confusions. I can easily raise several examples. For example, the Carrington storm is categorized as "fast transit event, low-latitude aurora" based on these catalogs. However, in reality, the Carrington event had the greatest flare magnitude in comparison with any modern flares that Hudson et al. (2024) cataloged (DOI: 10.3847/2041-8213/acd853; DOI: 10.1093/mnras/staf967). Carrington event was also greater than any such storms, if not unique (DOI: 10.3847/1538-4357/ac2601). This is missed in Lefèvre et al. (2016), mostly because the aa index started only from 1868. Therefore, the Carrington event should be categorized as "solar flare, fast transit event, geomagnetic storm, low-latitude aurora"

The authors need to cite chief event studies or reviews for each event. The authors have omitted such reference during the revision. This should be reverted. It is always helpful for the readers to have references for dedicated event studies.

I have suggested DOI: 10.1051/0004-6361/202038272 for a reference of the events in the International GLE database. However, I realised that this dataabse missed the large GLEs in the 1940s. The authors can fill the datagap in the 1940s with DOI: 10.1098/rsta.2025.0346. In fact, the particle precipitations look the most important factor for their discussions. This addition will improve their statistics and discussion.

(Remarks on code availability)

Reviewer #3

(Remarks to the Author)
The authors have addressed all of my questions and concerns. The revisions have strengthened the manuscript, and I therefore recommend it for publication without reservation.

(Remarks on code availability)

Reviewer #4

(Remarks to the Author)
Review of Brönnimann et al.
Trends, Extremes and Imprint of Solar Storms in Daily Atlantic-European Jet Variability, 1725-2100

• What are the major claims of the paper? Are they novel and do you think that they represent a significant advance in the field?
The authors constructed indicators of European daily jet stream variations from 1725 to present based on historical sea level pressure observations from Padova, Uppsala, London and Berlin. Surface pressure data from these stations were used to develop indices that are related to the strength, tilt, and latitude of the Atlantic-European jet extension and associated blocking patterns. The indices explain large fractions of day-to-day temperature variability at other locations in Western Europe. The daily jet stream variations since 1725 can be compared with the results with climate model simulations. Of particular interest is comparisons with reports of extreme surface weather during intervals when the jet stream is unusually fast/slow or latitudinally deviated from the jets normal position.

Specific Focus of this Review: In addition to tropospheric dynamics, the authors investigate several facets of heliospheric and geospace phenomena to discern if and how these phenomena associate with variations in the north European tropospheric jet stream and surface wind/pressure. These facets include sudden stratospheric warming (SSW) events from 1940 to present (Table S4) and a combined list of 'solar storms' (Table S5) from the 1700's to 2004. Relational inferences about effects of the SSWs and solar storms are based on indice composites that represent tropospheric response to upper atmosphere forcing (Fig. 3).

• If the conclusions are not original, it would be helpful if you could provide relevant references.
I have provided a few additional references in the body of the review below
• Is the work convincing, and if not, what further evidence would be required to strengthen the conclusions?
I have suggested some sensitivity studies/tests in the review below

• On a more subjective note, do you feel that the paper will influence thinking in the field and be of interest to a wider community of researchers?
Yes I believe it will generate of lot of interest. Of particular note the authors present evidence of solar storm connection to shifting tropospheric jet stream location and intensity,

• If you recommend publication, please outline, in a paragraph or so, what you consider to be the outstanding features.
Please note I am recommendation publication after some major revisions/additions
The authors composite sudden stratospheric warming events back to the 1940's and strong 'solar storms' back to 1730 (using different evidence including low-latitude aurorae, geomagnetic or solar observations, see Methods, Table S5)
To my knowledge this is the first study to find effects on solar/space weather in the troposphere over extended intervals of weeks and months.

The new results come from the use of long record of proxies built around sea surface pressure variations go back to the 1700's and building such relational composites from these. This form of superposed epoch analysis provides insight about immediate and/or lagged responses in tropospheric wind/pressure patterns to upper atmosphere changes that are driven by SSW and or solar storms. Some of the most significant responses occur in the 30–80-day time frame.
Body of Review for SSW and Solar Storm Portion of the Manuscript

SUDDEN STRATOSPHERIC WARMINGS (SSWS) : SSWs are wintertime phenomena associated with polar vortex disruption, typically in the post winter solstice time frame and with continuation into early spring. Most SSWs occur in the northern hemisphere. The paragraph devoted to the SSW analysis (Fig 3a) reveals a weakening of the jet and significant equatorward shift in European sector jet location with a delay of ~ 10-30 days after first appearance of the SSW. The authors state this delay corresponds to the 'expected' downward disturbance propagation time from the intense polar vortex to the surface.
1. Minor Recommendation for SSW Senstivity: It would be useful to provide an example(s) of the effects of surface weather changes associated with SSWs. SSWs are often categorized as minor or major.
-Possible Sensitivity Analysis: Is there sufficient information from the 58 events to differetiate changes in jet intensity or structure between mild and major events?
-If yes do more major or intense SSW's hasten or slow downward propagation?

SOLAR STORMS: For the solar storms analysis, the authors make a commendable effort in joining disparate data sets from different authors for the analysis. As the authors suggest, this does appear to be a 'first-of-its-kind' decadal-to-century scale investigation of solar storm effects on the coupled upper atmosphere-troposphere system.
The authors combine several categories of 'solar storms' for purposes of compositing. These include: low-latitude aurora sightings, geomagnetic storms, fast-transit events, solar particle storms and solar flares. (Note these roughly in order of how long humans have been able to make reliable measurements in these categories)

2. Major Sensitivity question: Does the transition to more sophisticated measurements (and lack of such measurements in say the 1700 and 1800's) influence your results? Doing the analysis in two parts: 1700's to 1940 and 1940 to present may reveal a shift

3. Major concern and sensitivity recommendation: From looking at the sources of these, likely extreme events, I am assuming that the 'solar particle storms' listed are actually what are more commonly known as Ground Level Enhancements (GLEs). GLEs are a particularly intense form of the more general category of solar proton events, sometimes called solar energetic particle events (SPEs, SEPs). The analysis apparently does not include SPEs as a distinct category. These events are known sources of upper atmospheric-tropospheric coupling and should be addressed as a separate

phenomenon. See these recent papers and citations in bold below.
As a very quick way to address this shortcoming. I recommend that the authors perform a sensitivity analysis for the 'solar particle storms' (GLEs) to determine if these events contribute in a disproportionate manner compared to say geomagnetic storms in the same interval. GLEs tend to be short impulsive events that may have long duration effects.
Denton, M. H., Kivi, R., Ulich, T., Clilverd, M. A., Rodger, C. J., & von derGathen, P. (2018). Northern hemisphere stratospheric ozone depletion caused by solar proton events: The role of the polar vortex. Geophysical Research Letters, 45, 2115–2124. https://doi.org/10.1002/2017GL075966
Li, H., Li, Y., Wang, Y., Sun, J., & Wang, C. (2025). Impact of solar proton events on the stratospheric polar vortex in the Northern Hemisphere: A quantitative analysis. Journal of Geophysical Research: Space Physics, 130, e2024JA033068. https://doi.org/10.1029/2024JA033068
If GLEs do dominate, then the authors definitely need to include the more ubiquitous solar proton events in their analysis. Solar protons (distinct from GLEs) are an important source of ozone loss.
A list of solar proton events since the early 1980s (used in many studies) can be found at:
https://www.ngdc.noaa.gov/stp/satellite/goes/dataaccess.html (solar proton flux data onboard GOES satellites)

4. Major Recommendation: Please report thresholds (and units if any) for the different measures. For example, what level of 'aa' index is associated with the listed geomagnetic storms? Similar information should be provided for other categories. The data should be available in the tables from which the events were taken and could be added to Table S5

5. Major Recommendation 4: Please explain in general terms how the different solar storm categories produce ozone depletions or other phenomena relevant to the polar vortex and influence on the tropospheric jet. This could be added to the supplementary material if there is an issue with manuscript length. The effects of energetic particle (electron) precipitation from geomagnetic storms and the effects of solar proton events may have different time scale and paths ways from the upper atmosphere to the troposphere.

6. Minor Recommendation: In Table S5, the 'fast transit events' appear to describe fast eruptions from the Sun that intercept Earth. The table cited is attributed to Cliver, however I believe the table in this review article is directly copied from Gopalswamy (2005). Please check and attribute as appropriate. I believe this would be another name for what is currently called fast interplanetary shock events. Please elaborate and provide a threshold value for such events.

7. Major Recommendation: The authors cite Sukhodolov et al. as a single source of agreement/verification of the 70-80 day enhancement of jet strength. I recommend considering/including
Xu, X., Wang, Y., Wei, F. et al. The disrupted jet stream and its influence on flight time during solar proton events. Sci Rep 15, 22969 (2025). https://doi.org/10.1038/s41598-025-07137-9
I believe the results from the present study and the Xu et al study are consistent on shorter time frames, however Xu et al (2025) study a different longitude region and put more emphasis on latitudinal shift of the jet stream than enhancement of jet stream at higher latitudes. The Xu et al paper may contain important references for this study

8. Question: Can the authors explain the significant time (and effect) differences between SSWs and solar storm effects?

9. Minor Recommendation for a possible extreme event that may support the analysis
The event from mid -January 1938 was deeply studied by Hayakawa et al. (2021). The intensity and evolution of the extreme solar and geomagnetic storms in 1938 January. Astrophys J 909: 197.https://doi.org/10.3847/1538-4357/abc427. The event likely had multiply solar flares, and a sequence of magnetic storms that produced low latitude aurora for several nights, as well as a plausible 'solar particle storm'. Is there sufficient information from the compositing results to tell if this produced an intense tropospheric response?

Delores J Knipp

(Remarks on code availability)
I believe the request was for a review of the code written in R, which is not a software package/platform that I use. I do have a statistician colleague who works with R. I did not want to go outside of the review chain without checking with the editor.

Reviewer #5

(Remarks to the Author)
I think Brönnimann et al. present an interesting reconstruction of the Atlantic-European jet stream and possible influencing factors and effects, e.g. on extreme weather. The jet reconstruction and links to weather and extreme events is interesting and I have no major criticism there. I will focus my comments on the solar storm discussion which I find a little bit problematic. Nevertheless, after additional discussion of the uncertainties I think the manuscript can be recommended for publication.

I think the solar storm discussion is problematic as the lower panel in figure 3 (solar storm influence) is not very convincing to me. If there is a signal it might come after 80 days but why do the authors cut the figure off at 90 days when the supposed strengthening is the strongest?
In my opinion an additional problem is the selection of solar storms. Energetic particle events are only known for the last about 80 years of neutron monitor data and these events are very different from geomagnetic storms. The authors write that

both might affect ozone in the stratosphere but there are likely differences since energetic particle events involve mostly high-energy protons penetrating deeper into the atmosphere compared to electron precipitation events. To me it is not clear that both events should have the same effects (e.g altitude profile, effects on ozone etc are likely different). I think a more robust analysis would be to separate energetic particle events (the known ones) and the geomagnetic storm events (where we have data going further back & aurorae) to evaluate the possible effects and differences.
Sukhodolov et al. might point to a possible mechanism but they model an event that is two orders of magnitude bigger than any know solar energetic particle event. So it is not at all clear that this can serve as an explanation for a solar influence on the strength of the jet.

(Remarks on code availability)

Version 2:

Reviewer comments:

Reviewer #1

(Remarks to the Author)
This manuscript has been reasonably improved. I have only several comments for cosmetic improvements. I would expect no less from these authors.

Figure S9: Extreme FTE is misleading. Carrington event is also an EFT (transit time of ≈ 17.6 hours). The 1972 event should be the fastest transit event.

It is probably beneficial to put some more references that these reviews used to derive pertinent parameters, such as flare magnitude, Dst index, aa index, transit time, and auroral visibility extents.

1788 - DOI: 10.1093/pasj/psab079
1789 - DOI: 10.1016/j.asr.2021.05.001
1870 - DOI: 10.1029/2007JA012943
1872 - DOI: 10.3847/1538-4357/acc6cc
1882 - Replace Capron (1883) with DOI: 10.1002/2017SW001795
1940 - DOI: 10.1093/mnras/stab3615
1941 - DOI: 10.3847/1538-4357/abb772
1989 - DOI: 10.1029/2019SW002278

(Remarks on code availability)

Reviewer #4

(Remarks to the Author)
The authors have addressed all of my major concerns. In particular, they have separated Ground Level Enhancements (GELs) from the less energetic solar energetic particle events (SEP). They have performed sensitivity studies that include low latitude aurora, geomagnetic storms but higher flux,, and fast transit events. They find general consistency in the polar front jet stream response to these disturbance categories.

The authors have improved the supplementary table of solar event drivers (Table S5), and provided thresholds for each type of event.
They provide clear statements about the expected chain of events that connects earth-directted solar eruptions with polar vortex and jet stream dynamics.

Minor repair: Please note that references 68 and 69 are identical.

I recommend publication of the present version of the article.

Delores Knipp

(Remarks on code availability)

Reviewer #5

(Remarks to the Author)
I think this is an interesting study that provides useful insights into the past Atlantic-European jet stream and possible links to solar storms. The authors addressed all my comments to my satisfaction and I therefore recommend publication of the study.

I found a few smaller typos (resuls, minotors) and some missing or misplaced full stops (e.g. reference after full stop) that the authors should check again.

(Remarks on code availability)

Open Access This Peer Review File is licensed under a Creative Commons Attribution 4.0 International License, which permits use, sharing, adaptation, distribution and reproduction in any medium or format, as long as you give appropriate credit to the original author(s) and the source, provide a link to the Creative Commons license, and indicate if changes were made.
In cases where reviewers are anonymous, credit should be given to 'Anonymous Referee' and the source.
The images or other third party material in this Peer Review File are included in the article's Creative Commons license, unless indicated otherwise in a credit line to the material. If material is not included in the article's Creative Commons license and your intended use is not permitted by statutory regulation or exceeds the permitted use, you will need to obtain permission directly from the copyright holder.
To view a copy of this license, visit https://creativecommons.org/licenses/by/4.0/

Reviewer #1 (Remarks to the Author):

Summary

This is an interesting article that reconstructed the weather pattern in a high time resolution (including brand new datasets from Berlin) and assessed how solar storms in the past impacted the terrestrial weather. There were numerous studies who tried to model such interactions. However, these attempts have been in want of the data to either validate or criticize their models. The authors' study offers excellent data to critically reconsider such modeling efforts. This is ultimately worth publication, while there are several critical issues to be modified. I hope these comments are of some use.

:: Major Comments

(1) The term of "solar or geomagnetic storms" are a wide umbrella term. The authors explain that the solar storms affect nitrogen chemistry of the polar mesosphere and cause ozone destruction and strengthen the polar vortex. The authors need to clarify which aspects of the solar storms trigger such mechanisms. What do they mean here: solar flares, ground level enhancements (solar particle storms), geomagnetic storms, or everything? Phenomenologically, they are all different from one another. Perhaps in the appendix, the authors should explain when EEP get enhanced, citing not only Seppälä et al. (2025) but also DOI: 10.1038/ncomms6197, contextualizing solar flare, GLEs, and geomagnetic storms.
Eventually the mechanism proceeds through particles, but records do not reach far back. Since particle storms often co-occur with other aspects of solar storms that are observable and can be traced back, we use these aspects as proxies.

We change “solar and geomagnetic storms” to “solar storms” in the Abstract and in the last paragraph. In the results section, we change:

“We then composited strong solar or geomagnetic storms back to 1730 (see Methods, Table S5), which to our knowledge has not been attempted before. Solar storms affect the nitrogen chemistry of the polar mesosphere, which can lead to ozone destruction and strengthen the polar vortex.”

To:

“We then composited strong solar storms back to 1730 (using different evidence including low-latitude aurorae, geomagnetic or solar observations, see Methods, Table S5), which to our knowledge has not been attempted before. Solar storms are often accompanied by increased solar energetic particle fluxes or by geomagnetic storms that trigger energetic electron precipitation events. Both can affect the nitrogen chemistry of the polar mesosphere, which can lead to ozone destruction and strengthen the polar vortex.” Here we also cite the suggested Andersson paper

In the appendix, together with the new list (Comment 3), we provide more details on the selection of events.

(2) The Carrington storm is known the "strongest" as a solar flare (DOI: 10.3847/2041-8213/acd853; DOI: 10.1093/mnras/staf967) and a geomagnetic storm (DOI: 10.1051/swsc/2013053; DOI: 10.3847/1538-4357/ac2601), while the SEP signal is robustly unconfirmed (DOI: 10.1051/swsc/2023030; DOI: 10.1007/s41116-023-00036-z). If the authors wish to raise the Carrington storm as the strongest solar particle storm in our period, they may wish to cite DOI:10.1029/2023GL106632 for a potential 14C signal detection. However, I personally know that the ice core data have shown a negative result and their interpretation will be rejected in the nearest future. The authors should clarify these different aspects of the Carrington storm, citing the said articles.
Thanks. Eventually it is the particle signal that is relevant for the atmospheric effect. If that is contested, then perhaps it is best not to show the Carrington storm, as it does not help much

anyway. The results of the composites (see replies below) are now more significant and the Carrington storm is removed. The figure is thus a bit simpler and the text shorter.

(3) The geographical classifications need some elaborations. Not too many readers would take Berlin as a representative of Eastern Europe. How do the authors define the geographical sector in Europe? How can these classifications get justified? They need some additional explanations.
Thanks.

We change "To capture the west-east gradient and the ridge pattern, an additional series in Eastern Europe is needed. We therefore searched for pressure data in Eastern Europe." to: "To capture the west-east gradient and the ridge pattern, an additional series further east is needed." (omitting the second sentence).

(4) The authors indicate that "extreme excursions of the jet were always associated with the extreme weather" in the abstract. It will make their statement more convincing if they list such extreme excursions above certain thresholds that are justified with proper references and pair all such extreme weather tp each case. This can be an additional supplementary table.
Thanks for the comment. In the revised paper we go into more detail. We show statistics not only for precipitation extremes, but also for hot and cold extremes as well as for wind extremes. We select all cases above thew 98th percentile (below the 2nd percentile) for each grid cell and then plot the average of the indices at this grid cell. This confirms the close association of extreme weather with strong deviation in the jet indices. The link is particularly strong for wind extremes, but also for temperatures and heavy rainfall. This figure also shows that the selected extreme examples (Fig. below) are indeed typical. Fig. S4 (now S5) is correspondingly extended and the text is adjusted.

We also go the other way and composite different variables for extremes (>98th percentile, <2nd percentile) of the indices. This is done for blocking frequency and for the jet stream location. All this shows the close association between jet excursions and extreme weather.

(5) Table S5 is crucial for their analyses for the impact of the solar storm to the terrestrial impacts. We need to be extra careful for this table, as this is one of the key datasets for this article.

Firstly, the authors must not rely on Wikipedia for such events. The authors need to rely on refereed articles or at least some meaningful datasets for their analyses.

While Cliver and Svalgaard (2004) have been a benchmark, their results have been somewhat outdated owing to the research development in the last two decades. The authors are selecting these events for solar flares, fast-transit events, low-latitude aurorae, geomagnetic storms, and solar particle storms. Ideally, the authors should clarify thresholds for each of these aspects and generate a homogenized dataset. However, I know it takes ages.

For the time being, I would suggest the authors to use the following catalogs.
:: solar flares -- Table 7 of DOI: 10.1007/s11207-024-02287-x
:: fast-transit events -- Table 5 of DOI: 10.1007/s41116-022-00033-8
:: solar particle storms -- Table 1 of DOI: 10.3847/1538-4357/abad93 and Table 1 of DOI: 10.1051/0004-6361/202038272
:: geomagnetic storms -- Table 2 of DOI:10.1007/s11207-016-0892-3 for the aa index and Table 7 of DOI: 10.1007/s41116-022-00033-8
:: low-latitude aurorae -- Figure 17 of DOI: 10.3847/1538-4357/ad9335 and Table 4 of DOI: 10.1007/s11214-023-01018-1

For the latest storms, the author should cite review articles such as DOI:10.3847/1538-4357/ad9335 for the May 2024 storm and DOI: 10.3389/fspas.2024.1522139 for the Oct 2024 storm.

Thank, we now work with the suggested list of storms. This increases the number of events from 36 to 99. Compositing the indices for this increased sample of solar storms, we find more significant results. The jet strength signal that was just marginally significant is now more clearly significant. The other two indices show now response (they also were not significant in the previous draft). The text is rewritten.

“Results show a significant strengthening of the jet after 80 days(Fig. 3). Hence, our results are consistent with an impact of solar storms on the jet stream and thus a link between space weather and Earth’s weather.”

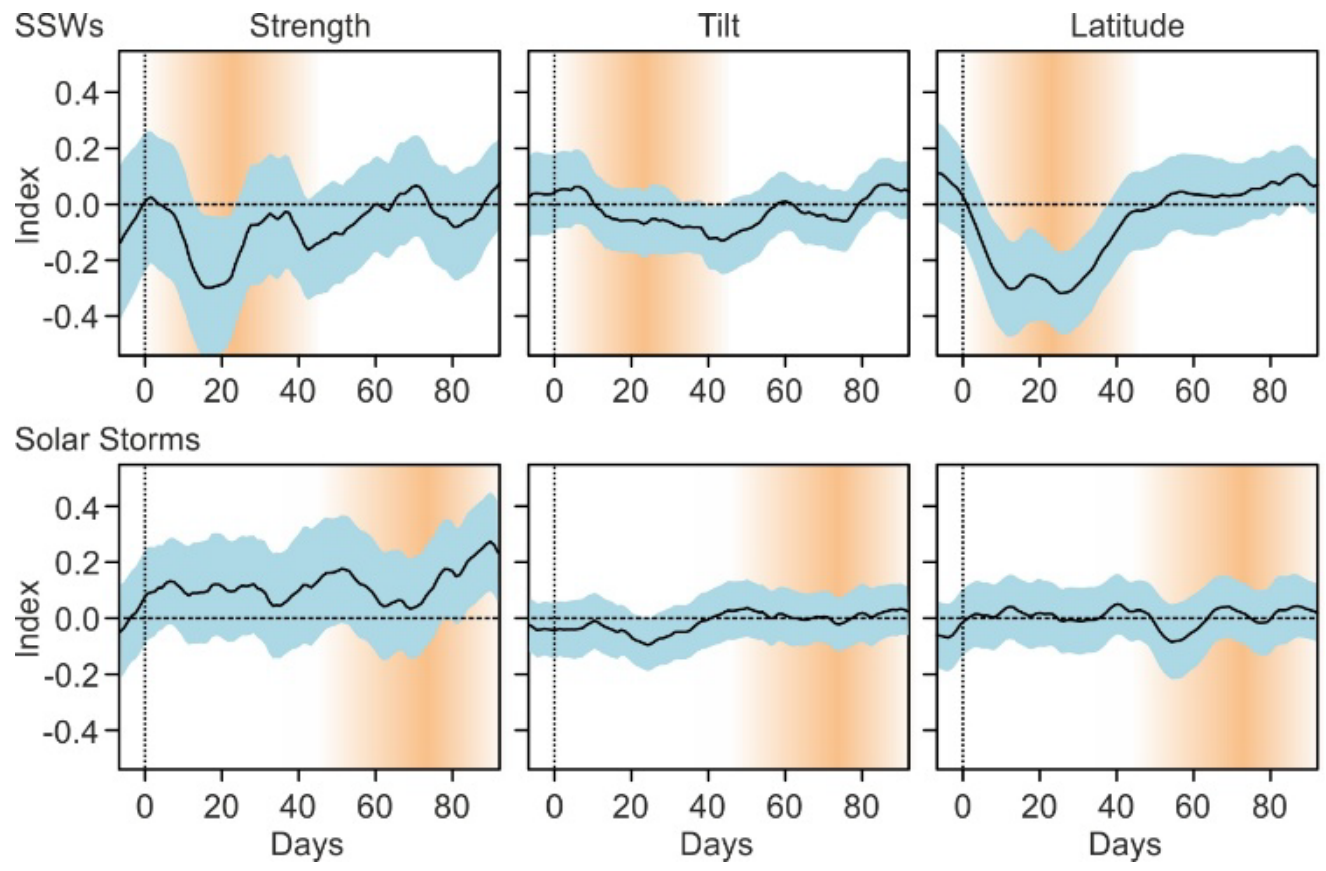

Fig. Composites of 15-day averaged indices for SSWs or solar storms. Top: Composites for 58 SSWs since 1940 (Table S4). Bottom: Composites for 99 solar or geomagnetic storms since 1730 that occurred in the September-March period (Table S5). Lines show the composite mean, shading indicates the 95% confidence interval of the mean. The orange shading approximately indicates the expected time scale of an effect on tropospheric weather via downward propagation of polar vortex changes.

:: Minor Comments

Page 2 Paragraph 1: What do the authors mean the reanalysis data? Do they mean the official data from the modern authorities? They should elaborate the explanation here.

Reanalysis data are generated by combining observations with climate model simulations using methods of data assimilation, which are typically Bayesian methods. Yes, typically only very large centres (ECMWF, NASA, etc.) are able to produce such data sets. Space is too short to give a full description of what a reanalysis is. Note also that reanalysis data are arguably the most widely used data in all of geosciences and thus widely known to geoscientists, but perhaps less well known to readers from other disciplines.

In the text we replace “Reanalysis data” with “three-dimensional data from atmospheric reanalyses such as ERA5” (and give a reference to ERA5).

Some of the quotes to historical records are difficult to trace. The authors should elaborate their referencing to the Portland Guardian (with the release date and page number) and the Monthly Weather Review (volume and pages).

We now give volume and pages: *Portland Guardian* (Fri, 13 Nov. 1891, p. 2), *Mon. Wea. Rev.* **35** (1907), p. 1.

Page 6 Paragraph 4: The authors need to clarify how they selected the sudden

We use the criteria of Charlton and Polvani, namely a a reversal of the zonal mean zonal wind at 10 hPa at 60° N. This is indicated in the text and in the caption of Table S4. While these authors use the period 1958-2002, we now a have more recent data set (ERA5) that covers 1940 to present and we can apply the same definition. For pre-1958 cases, for which ERA5 is arguably more uncertain, we searched for additional confirmation in the literature, which we cite in Table S4. This is all quite well explained already in the submitted paper. Perhaps a source of misunderstanding could be the place

of the remark in brackets, which we now shift to make it more clear. We changed the sentence Rather than “We analysed 58 SSWs since 1940 (Table S4) and composited the 15-day filtered jet indices around the dates of the SSWs (defined as a reversal of the zonal mean zonal wind at 10 hPa at 60° N).” we now write:

“We analysed 58 SSWs (defined as a reversal of the zonal mean zonal wind at 10 hPa at 60° N) since 1940 (Table S4) and composited the 15-day filtered jet indices around the dates of the SSWs (first day of the reversal).”

Page 7 Paragraph 1: Are the authors surveying the storms between September and March, as the CMIP6 model project a strengthening and narrowing of the Northern Atlantic jet in winter? It would be nice to clarify the reason of this selection.
A signal is only expected if stratosphere-troposphere coupling is possible. This is typically between around November to March, when lower stratospheric winds at mid-latitudes are westerly. For solar storms, which interact higher up in the atmosphere, we have a varying but possibly longer downward propagation time than for SSWs, we therefore start the window already in September, when the mesosphere-stratosphere vortex forms, in agreement with Sukhodolov that is cited in the sentence before. We now justify this better.

The sentence “For the compositing, to accommodate for longer downward propagation times, we considered all 36 events between September and March.”
Is changed to
“For the compositing, we focused on the active season of stratosphere-troposphere coupling, i.e., winter and early spring, but accounted for possibly longer downward propagation times and considered all 99 events between September, when the mesosphere-stratosphere Arctic polar vortex typically forms, and March.”

Reviewer #2 (Remarks to the Author):

Review of "Trends, Extremes and Imprint of Solar Storms in Daily Atlantic-European Jet Variability, 1725-2100" bx Bronnimann et al.

S. Bronnimann et al. define a new index that describe the characteristics (strength, tilt, and latitude) of the jet stream over the Atlantic-European region. The index is based on existing and newly made available datasets (four deseasonalized and standardized sets) from different locations over western Europe.

The authors study observation based daily jet stream variations since 1725 and compare it with climate model simulations to investigate past and future trends. The authors corelate their indices with climate data and investigate the influence of the jet stream on weather extremes, the effect of solar and geomagnetic activity on the jet stream parameters, and try to predict future variability of the jet.

Their results show that weather extremes over western Europe were always corelated with excursions of the jet stream. Their indices are able to explain a large part of day-day temperature variability and imprints of sudden stratospheric warmings due to solar induced geomagnetic storms were found (although considered not significant).
Not being an expert in this field, I find their study very interesting and worth being published. The results seem quite robust but I honestly think that the manuscript needs to be reviewed by other climate modelers or atmospheric scientists.
While I support publication of their results with some minor revisions, I focus my review on the construction of the observational dataset and a few general points.

In general, the manuscript often uses unexplained acronyms, which seem to be obvious in the climate modeller community but may not be so for the broad readership of Nature. So please go through the manuscript and check e.g., explain CMIP6; 20CRv3, SLP, etc.
Thanks.

SLP, 20CRv3, and CMIP6 are now explained at their first occurrence.

Methods:
P 11: Data was aggregated to daily means – if the data was non evenly distributed, how was this done? Using interpolation or weighted averages?
Thanks for the comment. No weighting was applied. If there were two or more measurements (typically early morning and late afternoon, sometimes morning, noon, evening) these were simply averaged.

We change "aggregated" to "averaged".

Outlier test: series of tests were conducted to detect outliers (which tests?), why did the authors choose asymmetric exclusion criteria ? -5/+4 SD? "Most outliers were kept= - can you say how many % were discarded/kept?
Thanks, we were indeed very short and are happy to provide more information. The text is expanded to answer all of these questions and now reads:
"We then performed a sequence of outlier tests marking measurements (1) outside the interval 950-1060 hPa, (2) outside the interval -5/+4 standard deviations (as negative extremes are more common), (3) steps of 30 hPa or more in a day an (4) four or more consecutive identical values. All 2761 marked measurements were analysed individually by analysing two or more time steps before and after and by analysing all available stations. Most of the marked measurement were kept as they were well in line with the other stations. Only about 5% were eventually flagged."

What exactly does “homogenized data” mean?, please say very briefly

Thanks. It is not fully clear which instance in the text was meant. Arguably the reviewer refers to the homogenisation of the Berlin series, where we now add “(i.e., adjusted for breaks)”. The sentence continues with stating quite precisely what homogenisation mean.

The entire sentence now reads: “The Berlin series was homogenised (i.e., adjusted for breaks) by using several reference series from DWD after 1876 and from the HCLIM dataset before that. Each segment in Table S1 was first homogenised independently and then merged with the others by proceeding backward in time and adjusting the older segment according to the overlap with the more recent one.”

For the non-expert reader – please give a hint what Craddock and Pettit tests are; same for Wilcoxon test?

When mentioning the Craddock and Pettitt tests, we add the sentence
“The former is a graphical test based on the cumulative sum of differences to a reference series, whose shape indicates the type (step or trend) of an inhomogeneity as well as the breakpoints, the latter is a non-parametric test for break points.”

Furthermore, we change “Wilcoxon test” to “Wilcoxon rank sum test (which is equivalent to the Mann Whitney test”.

“breakpoints were detected using Craddock test with the support of some metadata” not easy to understand what was done - which metadata was used?

We added “...such as such as station relocations or instrument changes”. Table S7, which is quoted, gives more information.

Neuchatel was detrended – which trend was applied? hPa/month?

The trend was -0.58 hPa/yr. This number is now given in the text.

“all series were deseasonalized and standardised” just briefly state what was done - ...by doing “what”...?

Thanks for the comment. Again, we were too brief and now give more details: “by subtracting the first two harmonics of the seasonal cycle and dividing the residuals by the standard deviation”

“data gaps were filled with reanalysis data” how was this done exactly?

This was done based on the deseasonalised and standardised (DS) reanalysis data taken from the closest grid point at 12 UTC.

We are now more explicit and change “…filled remaining gaps with reanalysis data.” with “…filled remaining gaps with DS reanalysis data from the closest grid cell at 12 UTC”.

“For filling these gaps, we used an analog method augmented with an offline Ensemble Kalman Filter approach” please give at least a reference for that is it the same as an “an off-line Kalman Filter”? that appears in the line below?

Thanks for pointing to the inconsistency in the wording. Yes, it is the same.

We now use in both instances “off-line Ensemble Kalman Filter, and we give a reference.“

Reviewer #3 (Remarks to the Author):

The manuscript by Brönnimanne et al. addresses a timely and highly relevant topic. Discrepancies between circulation and simulated trends are mounting, casting doubt on projections. One of the big issues is the relatively short observational record, and this is precisely the issue tackled in this paper.

The authors use daily sea-level pressure (SLP) observations, including a newly digitised Berlin time series, as a fingerprint of the large-scale flow often referred to as the "jet." From these data, they construct indices characterising jet strength, latitude, and tilt. Overall, I find the paper to be original, well-crafted, and a valuable contribution to the literature. I have, however, a few concerns that I encourage the authors to consider.
Major points

My main concern is primarily terminological. The analysis is framed around "the jet", and the conclusions refer to it. However, the indices employed are defined using continental SLP data and are therefore most directly linked to continental circulation patterns, including westerly flow over Europe.

In winter, the North Atlantic jet stream is a well-defined dynamical structure, especially over the ocean and western Europe. In summer, however, the notion of a coherent, persistent jet is more ambiguous. The appearance of a well-defined jet in summer largely arises from temporal averaging, whereas the instantaneous flow is better described as a succession of short-lived, spatially varying "jetlets" (e.g., Gammon, 2001). In this context, I wonder whether what the authors refer to as the jet—especially in summer—would be more accurately described as continental westerly winds. Related to this point, "classical" definitions of the North Atlantic jet latitude and speed (e.g., Woollings et al., 2010) have been shown to be strongly correlated with the NAO. In contrast, the indices presented here exhibit only weak correlations with the NAO, further suggesting that they may be capturing a somewhat different aspect of the circulation.
These concerns do not invalidate the authors' results, which I find robust and relevant. Rather, they pertain to interpretation and framing. It may be more appropriate to refer explicitly to "westerly winds" or "continental westerly flow," and then demonstrate how the proposed indices relate to more classical jet-stream diagnostics during the period with sufficient data coverage (e.g., from 1950 onward). Such a clarification would, in my view, strengthen the paper and avoid potential conceptual ambiguity.

Thank you very much for this very valid comment. Indeed, what we capture with our data is the flow at the surface over the continent. Whether or not – and if so, how – this is connected to the "jet stream" is not obvious and needs to be shown.

We approach this comments from two sides. On the one hand, we indeed are more careful with terminology and introduce numerous changes. For instance, we now often use "circulation indices", "strength, tilt, and latitude indices" or simply "indices" rather than "jet indices". When speaking about the jet, we are more careful to point out that we capture the extension region of the Atlantic-European jet and not its core region and remind the reader that our data directly sample only the surface level. In the Abstract, the sentence "The four locations allow defining indices that are related to the strength, tilt, and latitude of the Atlantic-European jet." is complemented with "…of the Atlantic-European jet extension"
Second, we perform additional new analyses, not just on 500 hPa zonal wind as in the submitted paper, but tropopause level jet metrics, specifically the latitude and strength of the polar front at 315 K and the subtropical jet at 350 K (Rivoire et al., 2026, doi:10.1038/s43247-026-03262-z) based on a Lagrangian approach. Compositing extreme cases of our indices in these jet metrics shows that our indices are indeed closely related to variations in the polar front jet extension region. At the same time, the analysis also shows important asymmetries, seasonal differences and (non-)co-

locations of polar front jet and subtropical jet. The corresponding plot is shown below (it is now Fig. S2), and the explanatory text reads:

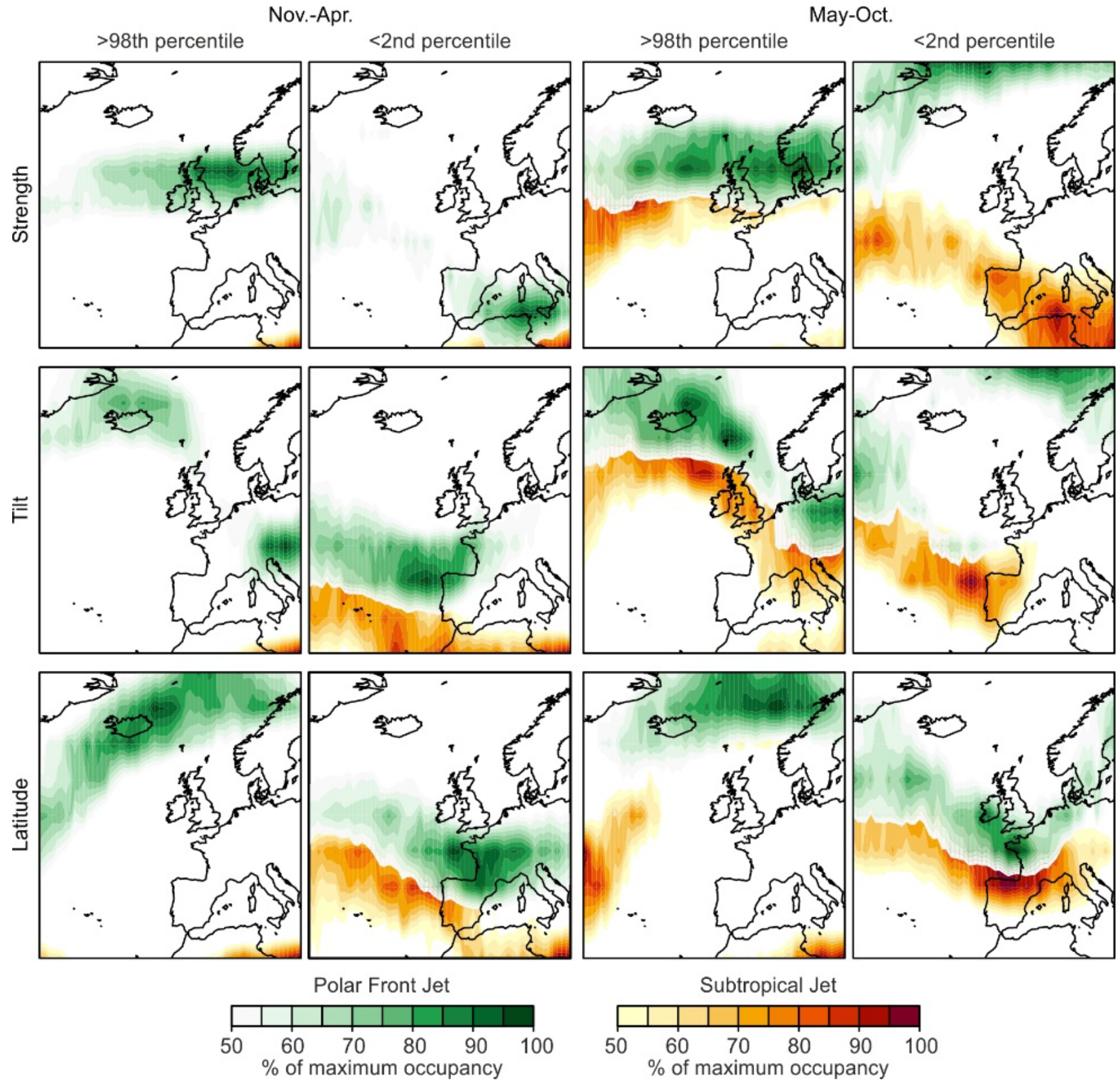

Fig. 1: Position of the PFJ and STJ (each field scaled to maximum occupancy in the field) 1941-2024 for extremes in the jet indices per season.

"We also analysed a data set of position and speed of the polar front jet (PFJ) at 315 K and of the subtropical jet (STJ) on 350 K based on a Lagrangian approach, covering 1941-2024. To address the nonlinearity, we composited jet positions for indices below the 2nd or above the 98th percentile per season (Fig. S2). For strong index cases, the PFJ is zonal, whereas for weak cases it is diverted to the Mediterranean in the cold season but to the North in the warm season. A positive tilt index is associated with a northwest-southeast tilt of the PFJ. The latitude index is associated with strong latitudinal shift in the PFJ. Hence, although our indices directly measure the continental westerly flow near the surface rather than the upper-level jet (which furthermore is not well defined in summer), they are statistically strongly associated with strength, tilt, and latitude of the Atlantic-European jet extension."

As to the NAO, we repeated the correlation analysis using daily NAO data back to 1836 from 20CRv3 and found cold season correlations of around 0.4, thus slightly higher as reported for the monthly means, but still low. This is arguably due to the different longitude represented by the diffetrent indices (Uppsala is 17.5° E in the jet extension region, Reykjavik is 22° W in the core region of the jet). The corresponding text (which now is on the "Methods" section) reads:

"Correlations with a daily North Atlantic Oscillation (NAO) index from 1836 to 2015 in the cold season are 0.393, 0.175 and 0.142 for strength, tilt, and latitude indices. This indicates that our indices are related to, but different from the classical NAO, arguably due to the different longitude (Uppsala is 17.5° E in the jet extension region, Reykjavik is 22° W in the core region of the jet). […] Averaging the daily indices to monthly means and then comparing them with the indices derived from monthly ERA5 500 hPa GPH fields yields high correlations (ca. 0.75) for strength and tilt in both seasons, but lower (ca. 0.62) for jet latitude. There is no one-to-one relation to monthly indices of

the NAO, the East Atlantic Pattern (EA), and the Scandinavian pattern (SCAND) of the NOAA Climate Prediction Centre (https://www.cpc.ncep.noaa.gov/data/teledoc/). The jet strength is weakly (0.31) correlated with the NAO and more strongly (-0.51) with SCAND, tilt is negatively correlated with both NAO (-0.44) and EA (-0.45), whereas latitude is weakly (0.28) correlated with EA."

Minor comments:
"In fact, the observed strengthening is greater than in any one of the CMIP6 simulations". See also Patterson and O'Reilly (2025)
Thanks, the reference is added

"Given the large underestimation of the strengthening by CMIP6 models in the historical period, there is little confidence in the projected future trends". See also Ossó et al., (2025)
Thanks, the reference is added

"Two-year moving time series of lag 1 autocorrelation and of the variance do not reveal obvious changes (Fig. S1)." I believe the authors refer here to Fig. S2.
Thanks, yes, it is Fig. S2.

References

Patterson, M., & O'Reilly, C. H. (2025). Climate models struggle to simulate observed North Pacific jet trends, even accounting for tropical Pacific sea surface temperature trends. Geophysical Research Letters, 52, e2024GL113561. https://doi.org/10.1029/2024GL113561

Nielsen-Gammon JW. A Visualization of the Global Dynamic Tropopause. Bull Am Meteorol Soc. 2001;82(6):1151–67. https:// doi. org/ 10. 1175/ 1520- 0477(2001) 082>1151:AVOTGD<2.3.CO;2.

Ossó, A., Bladé, I., Karpechko, A. et al. Advancing Our Understanding of Eddy-driven Jet Stream Responses to Climate Change – A Roadmap. Curr Clim Change Rep 11, 2 (2025). https://doi.org/10.1007/s40641-024-00199-3

Trends, Extremes and Imprint of Solar Storms in Daily Atlantic-European Jet Variability, 1725-2100

Reply to the reviewers' comments

Reviewer #1 (Remarks to the Author):
The authors have reasonably improved their manuscript. Still, their omissions though the revision process seem too radical. It is definitely better to pay more attention to the individual solar storms. I therefore have to suggest two major improvements to this manuscript.

(1) Their case studies on the weather response
This is particularly the case for their omission of the Carrington storm. It is definitely better and convincing to show the authors some concrete examples, apart from their statistical analyses.

The authors should revive their figure for the Carrington storm. Here, the authors need to describe this event as having the greatest flare magnitude (DOI: 10.3847/2041-8213/acd853; DOI: 10.1093/mnras/staf967) and the geomagnetic disturbance (DOI: 10.1051/swsc/2013053; DOI: 10.3847/1538-4357/ac2601) within the authors' catalog but lacking an evidence as the greatest particle storm (DOI: 10.1051/swsc/2023030).

The authors should add such figures for the greatest solar storms in the other categories (the greatest GLE and the fastest-transit event). For the greatest GLE, the authors should show their case analyses on GLE #5 on 23 Feb 1956 (DOI: 10.1051/0004-6361/202348699). For the fastest-transit event, the authors should show their case analyses on the solar storm on 4 Aug 1972 (DOI: 10.1029/2018SW002024; DOI: 10.3847/1538-4357/acf9fd).

Thanks for the comment. We now add a figure to the supplement showing the series for outstanding storms of different categories: solar flare and geomagnetic storms (Carrington event), low-latitude aurorae and multiple flares (1938 event, recommended by Reviewer 4 as a separate analysis), GLE (1956 event) and fast transit event (1972, although this is slightly earlier than our seasonal cut-off). We also add the references for individual storms back to our Table and include several additional storms (see reply to rev. #4). The new figure on the individual cases is consistent with the statistical analyses as the cases seem to indicate a positive response of the jet strength. However, it should be kept in mind that these are individual cases and the variability of the jet is very large. Statistical significance emerges only when compositing dozens of events. Even for a strong event, we may not necessarily see a signal.

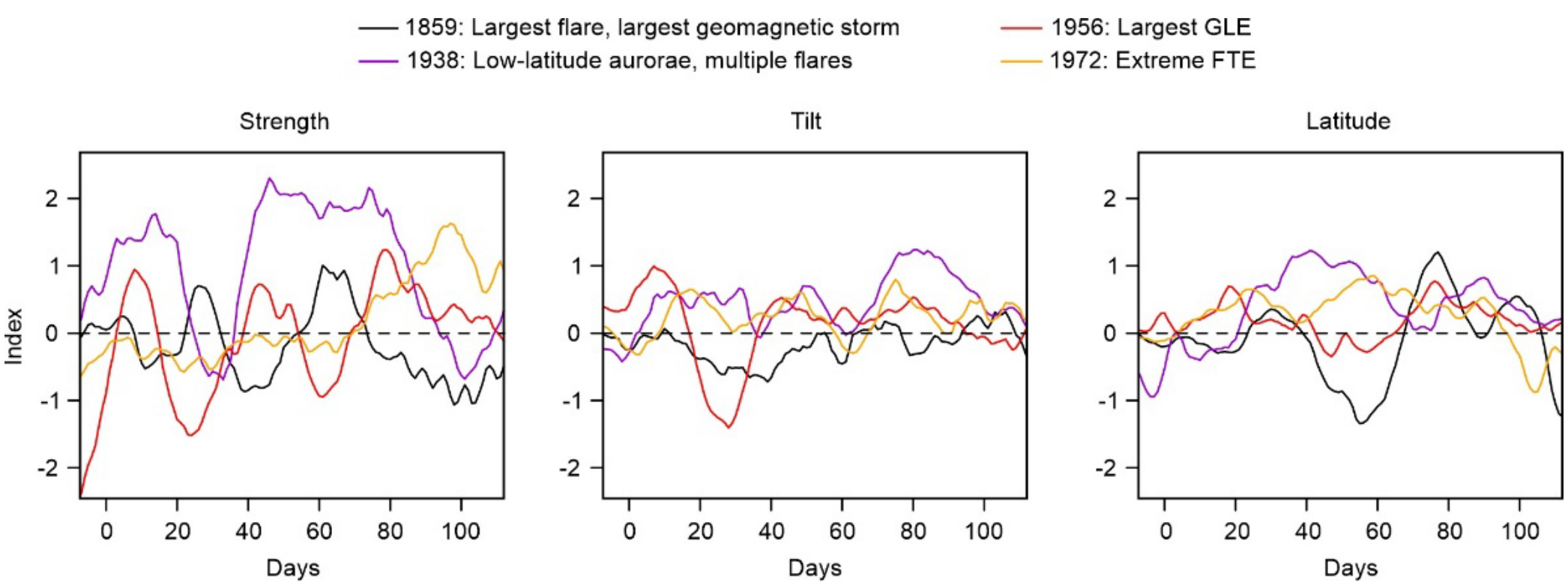

Fig. S9: 15-day averaged indices for the strongest solar storms. Shown are the Carrington event on 1 Sep 1859 (largest geomagnetic storm),[78-80] the low-latitude aurora 16 Jan 1938,84 GLE #5 on 23 Feb 1956,[85] and the fastest-transit event on 4 Aug 1972.[117]

(2) Table S5

Besides, the authors need to be mindful for their catalog. The authors need to explain that the table follows Cliver and Svalgaard's event category.

The reference is added back along with the sentence: "The selection largely follows the categorization of Cliver and Svalgaard.[67]"

While I suggested to use these catalogs, we definitely need to consult event studies and avoid any unwanted confusions. I can easily raise several examples. For example, the Carrington storm is categorized as "fast transit event, low-latitude aurora" based on these catalogs. However, in reality, the Carrington event had the greatest flare magnitude in comparison with any modern flares that Hudson et al. (2024) cataloged (DOI: 10.3847/2041-8213/acd853; DOI: 10.1093/mnras/staf967). Carrington event was also greater than any such storms, if not unique (DOI: 10.3847/1538-4357/ac2601). This is missed in Lefèvre et al. (2016), mostly because the aa index started only from 1868. Therefore, the Carrington event should be categorized as "solar flare, fast transit event, geomagnetic storm, low-latitude aurora"

The authors need to cite chief event studies or reviews for each event. The authors have omitted such reference during the revision. This should be reverted. It is always helpful for the readers to have references for dedicated event studies.

Thanks for this helpful comment. We changed the description of some of the storms, as suggested for the Carrington event. We also made the description more consistent. Furthermore, we add the GLE event number and we mark all events that are strong SPEs (>1000 pfu). Additionally, eight new events are added according to the suggestion of reviewer #4 (strong SPEs that are not GLEs). The table caption now also contains the definitions (thresholds) of the events.

Table S5. List of historical solar storms between September and March used in this study. The selection largely follows the categorization of Cliver and Svalgaard.[67] If several events occurred within less than 10 days, the first one is chosen. Sources: A = Table 7 in Cliver et al. (2022)[68] and B = Table 4 in Usoskin et al. (2023)[69] for low-latitude aurorae, C = Table 2 in Lefèvre et al. (2016)[70] for geomagnetic storms (peak aa > 300 nT), D = Table 3 in Gopalswamy et al. (2005)[71] for fast transit events (<30 hours), E = Table 1 in Cliver et al. (2020)[72] and F = Table 1 in Usoskin et al. (2020)[73] for GLEs (statistically significant enhancements of count rates of at least two differently located neutron monitors), G = Table 7 in Hudson et al. (2024)[74] for solar soft-X-ray flares (class X10, i.e., >10^{-3} W m^{-2}), H = NOAA Space Weather Prediction Center[75] for strong SPEs (>1000 pfu).

Year	Mon	Day	Event	Source	Reference
1730	2	15	low-latitude aurora	A, B	Hayakawa et al. (2018)[76]
1770	9	15	low-latitude aurora	A, B	Hayakawa et al. (2017)[77]
1788	10	21	low-latitude aurora	B	
1789	11	14	low-latitude aurora	B	
1848	11	17	low-latitude aurora	B	
1859	9	1	low-latitude aurora, geomagnetic storm, fast transit event, solar flare	A, B, D	Hayakawa et al. (2019, 2023)[78,79], Hudson et al. (2025)[80], Cliver and Dietrich (2013)[81]
1870	9	24	geomagnetic storm (aa index)	C	
1870	10	24	low-latitude aurora, geomagnetic storm (aa index)	B, C	
1871	2	12	geomagnetic storm (aa index)	C	Hayakawa et al. (2019)[78]
1872	2	4	low-latitude aurora, geomagnetic storm	A, B	Hayakawa et al. (2019)[78]
1872	10	14	geomagnetic storm (aa index)	C	Hayakawa et al. (2019)[78]

1881	1	31	geomagnetic storm (aa index)	C	
1882	11	17	geomagnetic storm (aa index)	C	Capron (1883)[82]
1886	3	30	geomagnetic storm (aa index)	C	
1892	2	14	geomagnetic storm (aa index)	C	
1894	2	25	geomagnetic storm (aa index)	C	
1898	3	15	geomagnetic storm (aa index)	C	
1898	9	9	geomagnetic storm (aa index)	C	
1903	10	31	geomagnetic storm (aa index)	A, C	Hayakawa et al. (2020)[83]
1907	2	9	geomagnetic storm (aa index)	C	
1908	9	10	geomagnetic storm (aa index), fast transit event	C, D	
1909	9	24	low-latitude aurora, geomagnetic storm (aa index), fast transit event	A, B, C, D	Hayakawa et al. (2019)[78]
1916	1	10	fast transit event	D	
1917	2	14	fast transit event	D	
1920	3	23	geomagnetic storm (aa index)	C	
1926	1	25	geomagnetic storm (aa index), fast transit event	C, D	
1926	10	15	geomagnetic storm (aa index)	C	
1938	1	16	low-latitude aurora, geomagnetic storm (aa index), fast transit event	B, C, D	Hayakawa et al. (2021)[84]
1940	3	24	low-latitude aurora, geomagnetic storm (aa index)	B, C	
1941	2	28	fast transit event	D	
1941	3	1	low-latitude aurora, geomagnetic storm (aa index)	A, C	
1941	9	17	geomagnetic storm (aa index), fast transit event	C, D	
1942	2	28	geomagnetic storm (aa index), fast transit event, solar particle storm (GLE#1,2)	C, D, E	
1944	12	16	geomagnetic storm (aa index)	C	
1946	2	6	geomagnetic storm (aa index), fast transit event	C, D	
1946	3	25	low-latitude aurora, geomagnetic storm (aa index)	A, C	
1946	9	22	geomagnetic storm (aa index)	C	
1948	10	17	geomagnetic storm (aa index)	C	
1949	1	25	geomagnetic storm (aa index)	C	
1949	11	19	solar particle storm (GLE#4)	E	
1951	10	28	geomagnetic storm (aa index)	C	
1956	2	23	solar particle storm (GLE#5)	E, F	Hayakawa et al. (2024)[85]
1957	1	20	geomagnetic storm (aa index), fast transit event	C, D	
1957	3	2	geomagnetic storm (aa index)	C	
1957	9	4	geomagnetic storm (aa index)	C	
1957	9	29	geomagnetic storm (aa index)	C	
1958	2	9	low-latitude aurora, geomagnetic storm (aa index), fast transit event	B, C, D	
1958	9	4	geomagnetic storm (aa index)	C	
1959	3	27	geomagnetic storm (aa index)	C	
1960	9	3	solar particle storm (GLE#9)	E	
1960	10	6	geomagnetic storm (aa index)	C	
1960	11	12	geomagnetic storm (aa index), fast transit event, solar particle storm (GLE#10-12)	C, D, E, F	
1961	10	28	geomagnetic storm (aa index)	C	
1963	9	22	geomagnetic storm (aa index)	C	
1967	1	28	solar particle storm (GLE#16)	E, F	
1967	2	16	geomagnetic storm (aa index)	C	
1968	9	29	solar particle storm (GLE#18)	E, F	
1968	11	18	solar particle storm (GLE#19)	E, F	
1969	2	2	geomagnetic storm (aa index)	C	
1969	2	25	solar particle storm (GLE#20)	E, F	
1969	3	30	solar particle storm (GLE#21)	E, F	
1970	3	8	geomagnetic storm (aa index)	C	
1971	1	24	solar particle storm (GLE#22)	E, F	
1971	9	1	solar particle storm (GLE#23)	E, F	
1977	9	19	solar particle storm (GLE#28)	E, F	

1977	9	24	solar particle storm (GLE#29)	E, F	
1977	11	22	solar particle storm (GLE#30)	E, F	
1978	9	23	solar particle storm (GLE#32)	E, F, H	
1981	10	12	solar particle storm (GLE#36)	E, F, H	
1982	9	6	geomagnetic storm (aa index)	C	
1982	11	26	solar particle storm (GLE#37)	E, F	
1982	12	8	solar particle storm (GLE#38)	E, F, H	
1982	12	17	solar flare	G	
1984	2	16	solar particle storm (GLE#39)	E, F	
1986	2	8	geomagnetic storm (aa index)	C	
1989	3	6	solar flare (strong SPE 8 Mar)	G, H	
1989	3	13	low-latitude aurora, geomagnetic storm (aa index), strong SPE 17 Mar	A, B, C, H	
1989	9	29	solar particle storm (GLE#42)	E, F, H	
1989	10	19	geomagnetic storm (aa index), solar particle storm (GLE#43), solar flare	C, E, F, G, H	
1989	11	15	geomagnetic storm (aa index), solar particle storm (GLE#46)	C, E, F	
1989	11	30	strong SPE	H	
1991	1	25	solar flare	G	
1991	3	24	geomagnetic storm (aa index), strong SPE	C, H	
1991	11	8	geomagnetic storm (aa index)	C	
1992	9	9	geomagnetic storm (aa index)	C	
1992	11	2	solar particle storm (GLE#54)	E, H	
1994	2	20	strong SPE	H	
1997	11	6	solar particle storm (GLE#55)	E, F	
1998	9	30	strong SPE	H	
2000	9	17	geomagnetic storm (aa index)	C	
2000	11	8	strong SPE	H	
2001	9	24	strong SPE	H	
2001	11	4	geomagnetic storm (aa index), solar particle storm (GLE#62)	C, E, F, H	
2001	11	24	geomagnetic storm (aa index), strong SPE	C, H	
2001	12	26	solar particle storm (GLE#63)	E, F	
2003	10	26	geomagnetic storm (aa index), fast transit event, solar particle storm (GLE#65,66), solar flare	B, C, E, F, G, H	
2003	11	4	solar particle storm (GLE#67), solar flare	G, H	
2003	11	20	geomagnetic storm (aa index)	C	
2004	11	8	geomagnetic storm (aa index)	C	
2005	1	17	solar particle storm (GLE#68, 69)	E, F, H	
2005	9	7	solar flare, strong SPE	G, H	
2006	12	13	solar particle storm (GLE#70)	E, F, H	
2012	1	23	strong SPE	H	
2012	3	7	strong SPE	H	
2014	1	6	strong SPE	H	
2017	9	6	solar particle storm (GLE#72), solar flare	E, F, G	Zhu et al. (2021)[86]
2024	10	11	geomagnetic storm, strong SPE	H	Oliveira et al. (2025)[87]

I have suggested DOI: 10.1051/0004-6361/202038272 for a reference of the events in the International GLE database. However, I realised that this dataabse missed the large GLEs in the 1940s. The authors can fill the datagap in the 1940s with DOI: 10.1098/rsta.2025.0346. In fact, the

particle precipitations look the most important factor for their discussions. This addition will improve their statistics and discussion.

Thanks for the comments. Three of the four cases are already included (note that GLE#1 and 2 were too close together to be separated according to our criterion of a minimum 10-day distance). The fourth case in 25 July 1946 is outside of our seasonal window.

Reviewer #3 (Remarks to the Author):
The authors have addressed all of my questions and concerns. The revisions have strengthened the manuscript, and I therefore recommend it for publication without reservation.

Thanks for this very positive comment.

Reviewer #4 (Remarks to the Author):

Review of Brönnimann et al.
Trends, Extremes and Imprint of Solar Storms in Daily Atlantic-European Jet Variability, 1725-2100

• What are the major claims of the paper? Are they novel and do you think that they represent a significant advance in the field?
The authors constructed indicators of European daily jet stream variations from 1725 to present based on historical sea level pressure observations from Padova, Uppsala, London and Berlin. Surface pressure data from these stations were used to develop indices that are related to the strength, tilt, and latitude of the Atlantic-European jet extension and associated blocking patterns. The indices explain large fractions of day-to-day temperature variability at other locations in Western Europe. The daily jet stream variations since 1725 can be compared with the results with climate model simulations. Of particular interest is comparisons with reports of extreme surface weather during intervals when the jet stream is unusually fast/slow or latitudinally deviated from the jets normal position.

Specific Focus of this Review: In addition to tropospheric dynamics, the authors investigate several facets of heliospheric and geospace phenomena to discern if and how these phenomena associate with variations in the north European tropospheric jet stream and surface wind/pressure. These facets include sudden stratospheric warming (SSW) events from 1940 to present (Table S4) and a combined list of 'solar storms' (Table S5) from the 1700's to 2004. Relational inferences about effects of the SSWs and solar storms are based on indice composites that represent tropospheric response to upper atmosphere forcing (Fig. 3).

• If the conclusions are not original, it would be helpful if you could provide relevant references.
I have provided a few additional references in the body of the review below
• Is the work convincing, and if not, what further evidence would be required to strengthen the conclusions?
I have suggested some sensitivity studies/tests in the review below

• On a more subjective note, do you feel that the paper will influence thinking in the field and be of interest to a wider community of researchers?

Yes I believe it will generate of lot of interest. Of particular note the authors present evidence of solar storm connection to shifting tropospheric jet stream location and intensity,

• If you recommend publication, please outline, in a paragraph or so, what you consider to be the outstanding features.
Please note I am recommendation publication after some major revisions/additions
The authors composite sudden stratospheric warming events back to the 1940's and strong 'solar storms' back to 1730 (using different evidence including low-latitude aurorae, geomagnetic or solar observations, see Methods, Table S5)
To my knowledge this is the first study to find effects on solar/space weather in the troposphere over extended intervals of weeks and months.

The new results come from the use of long record of proxies built around sea surface pressure variations go back to the 1700's and building such relational composites from these. This form of superposed epoch analysis provides insight about immediate and/or lagged responses in tropospheric wind/pressure patterns to upper atmosphere changes that are driven by SSW and or solar storms. Some of the most significant responses occur in the 30–80-day time frame.
Body of Review for SSW and Solar Storm Portion of the Manuscript

SUDDEN STRATOSPHERIC WARMINGS (SSWS) : SSWs are wintertime phenomena associated with polar vortex disruption, typically in the post winter solstice time frame and with continuation into early spring. Most SSWs occur in the northern hemisphere. The paragraph devoted to the SSW analysis (Fig 3a) reveals a weakening of the jet and significant equatorward shift in European sector jet location with a delay of ~ 10-30 days after first appearance of the SSW. The authors state this delay corresponds to the 'expected' downward disturbance propagation time from the intense polar vortex to the surface.
1. Minor Recommendation for SSW Senstivity: It would be useful to provide an example(s) of the effects of surface weather changes associated with SSWs. SSWs are often categorized as minor or major.

Thanks for the detailed and constructive comments. We added a sentence on the mechanisms and the surface weather effects including an additional reference to extreme weather events in surface weather (see below) and we now also address minor warmings, as explained in the next comment.

"Major SSWs, defined as a collapse of the polar vortex in the upper stratosphere, can propagate downward by wave-mean flow interaction, eventually affecting the westerly circulation in the troposphere and extreme weather."

Domeisen, D.I.V., Butler, A.H. Stratospheric drivers of extreme events at the Earth's surface. *Commun Earth Environ* **1**, 59 (2020). https://doi.org/10.1038/s43247-020-00060-z

-Possible Sensitivity Analysis: Is there sufficient information from the 58 events to differetiate changes in jet intensity or structure between mild and major events?
-If yes do more major or intense SSW's hasten or slow downward propagation?
Thanks for this comments. All chosen event were major warmings, i.e., the polar vortex in the stratosphere collapsed. Minor warmings (defined as sequences of at least 5 days during which the temperature gradient between 60° and 90° N reverses at 10 hPa while the wind does not reverse) are not expected to have an influence on the troposphere. We now calculated these events from ERA5 and also composited the indices. So, we incorporated the following changes:

(1) A very recent paper (Lu and Rao, 2026) published a list of sudden stratospheric warmings based on ERA5, October 1940 to December 2022, that is almost identical to ours. We extend it backward to January 1940 based on ERA5 (and supported by direct observations) and forward to 2024, which adds three events. Thus we now have a list that is consistent with the literature.
(2) An advantage of this selection is that Lu and Rao (2026) additionally partitioned the events into "downward propagating" and "not downward propagating", which we can use to test whether our indices indeed capture the downward propagation.
(3) We calculated composites for minor warmings as described above.

The results show significant effects of major SSWs in our indices, as in the submitted paper. In addition, by adding two additional lines, we show that minor warmings have no effect and that "downward propagating SSWs" have the largest effect, as expected. This shows that our indices capture downward propagating changes in the polar vortex.

The new Table and Figure are shown below.

Table S4. List of SSW events used in this study. We used the list of Lu and Rao[31] which is based on Charlton and Polvani[65], extended to the period October 1940 to December 2022 using ERA5. We added two earlier events in 1940 based on ERA5, consistent with reported SSWs in Brönnimann et al.[66], and one later event. Events described as "downward propagating" in Lu and Rao[31] are marked in bold.

y	M	d	Y	m	D	Y	m	d
1940	1	11	**1968**	**11**	**28**	2000	3	20
1940	2	9	1969	3	13	**2001**	**2**	**11**
**1941**	**2**	**7**	**1970**	**1**	**2**	2001	12	30
1942	3	21	**1971**	**1**	**18**	**2002**	**2**	**17**
1945	3	19	**1971**	**3**	**20**	2003	1	18
1946	2	19	1973	1	31	**2004**	**1**	**5**
1946	3	19	**1977**	**1**	**9**	**2006**	**1**	**21**
1950	3	5	**1979**	**2**	**22**	2007	2	24
**1952**	**2**	**22**	**1980**	**2**	**29**	**2008**	**2**	**22**
1952	11	19	1981	3	4	**2009**	**1**	**24**
**1954**	**12**	**18**	1981	12	4	**2010**	**2**	**9**
**1955**	**1**	**26**	**1984**	**2**	**24**	**2010**	**3**	**24**
**1957**	**2**	**4**	**1985**	**1**	**1**	**2013**	**1**	**6**
**1958**	**2**	**1**	**1987**	**1**	**23**	**2018**	**2**	**12**
**1960**	**1**	**17**	1987	12	8	**2019**	**1**	**1**
1963	1	27	1988	3	14	**2021**	**1**	**5**
1965	12	16	1989	2	21	2023	2	16
**1966**	**2**	**22**	1998	12	15			
**1968**	**1**	**7**	**1999**	**2**	**26**			

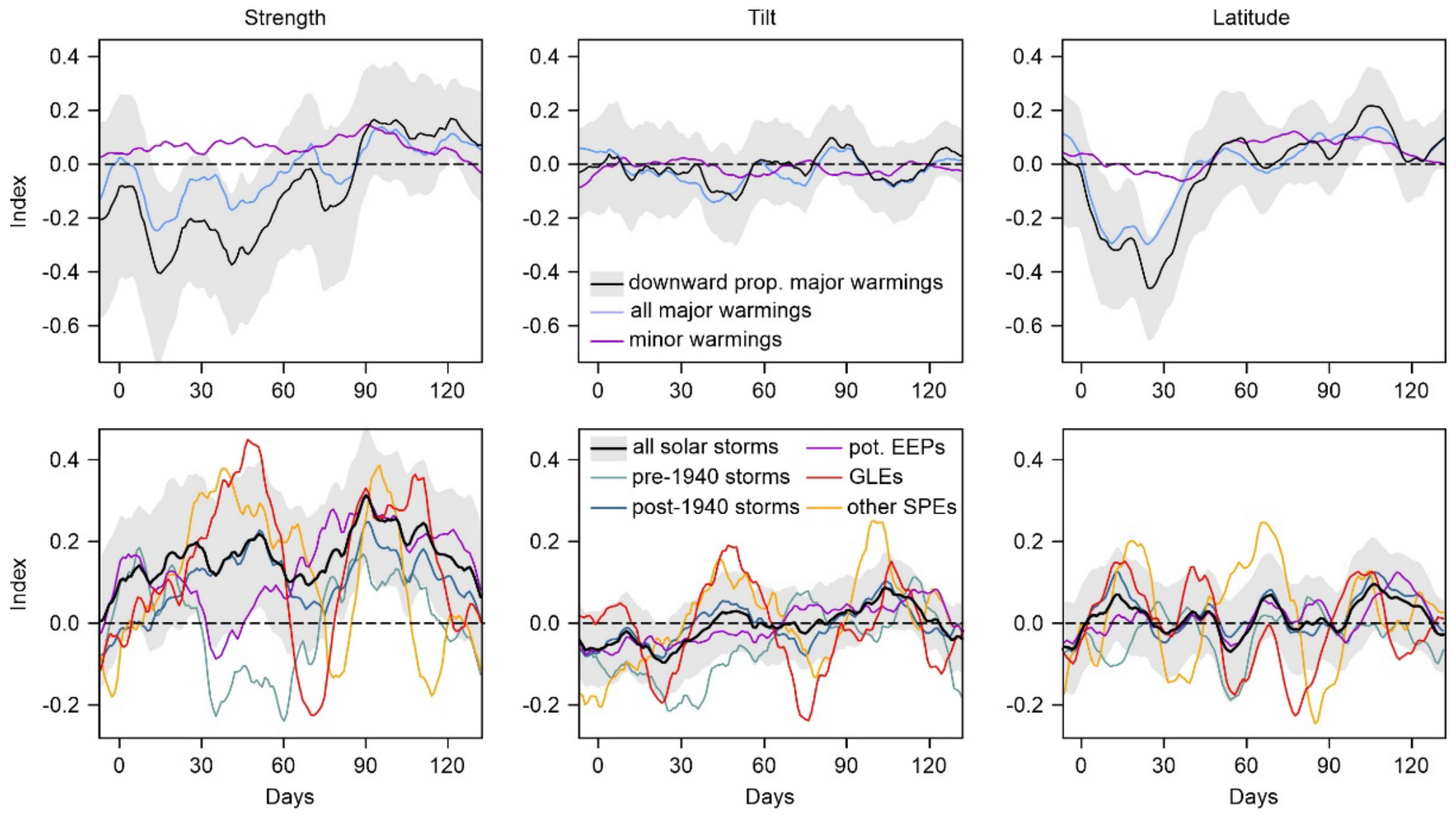

Fig. 3. Composites of 15-day averaged indices for SSWs or solar storms. Top: Composites for 55 major SSWs since 1940, of which 33 are characterised as downward propagating[31] (Table S4), and 176 minor warming events. Bottom: Composites for 107 solar or geomagnetic storms since 1730 that occurred in the September-March period (Table S5) as well as for five subgroups (pot. EEPs comprise low-latitude aurorae, geomagnetic storms, and fast transit events). Lines show the composite mean, shading indicates the 95% confidence interval of the mean.

SOLAR STORMS: For the solar storms analysis, the authors make a commendable effort in joining disparate data sets from different authors for the analysis. As the authors suggest, this does appear to be a 'first-of-its-kind' decadal-to-century scale investigation of solar storm effects on the coupled upper atmosphere-troposphere system.
The authors combine several categories of 'solar storms' for purposes of compositing. These include: low-latitude aurora sightings, geomagnetic storms, fast-transit events, solar particle storms and solar flares. (Note these roughly in order of how long humans have been able to make reliable measurements in these categories)

2. Major Sensitivity question: Does the transition to more sophisticated measurements (and lack of such measurements in say the 1700 and 1800's) influence your results? Doing the analysis in two parts: 1700's to 1940 and 1940 to present may reveal a shift
Thanks for this comment. We performed a number of sensitivity tests, and the results are now all included in the main figure (note that all sensitivity composites were centred to zero mean during the analysed subperiod, October to April, because most cover only subperiods and there might be a long-term trend in the index). The pre-1940 list contains only 28 events versus 79 in the post 1940 era. As a consequence the pre-1940 composite is more variable, but it also shows a stronger polar vortex after ca. 80-100 days. All analyses are consistent.

The revised figure is shown above.

3. Major concern and sensitivity recommendation: From looking at the sources of these, likely extreme events, I am assuming that the 'solar particle storms' listed are actually what are more commonly known as Ground Level Enhancements (GLEs). GLEs are a particularly intense form of

the more general category of solar proton events, sometimes called solar energetic particle events (SPEs, SEPs). The analysis apparently does not include SPEs as a distinct category. These events are known sources of upper atmospheric-tropospheric coupling and should be addressed as a separate phenomenon. See these recent papers and citations in bold below.
As a very quick way to address this shortcoming. I recommend that the authors perform a sensitivity analysis for the 'solar particle storms' (GLEs) to determine if these events contribute in a disproportionate manner compared to say geomagnetic storms in the same interval. GLEs tend to be short impulsive events that may have long duration effects.
Denton, M. H., Kivi, R., Ulich, T., Clilverd, M. A., Rodger, C. J., & von derGathen, P. (2018). Northern hemisphere stratospheric ozone depletion caused by solar proton events: The role of the polar vortex. Geophysical Research Letters, 45, 2115–2124.
https://doi.org/10.1002/2017GL075966
Li, H., Li, Y., Wang, Y., Sun, J., & Wang, C. (2025). Impact of solar proton events on the stratospheric polar vortex in the Northern Hemisphere: A quantitative analysis. Journal of Geophysical Research: Space Physics, 130, e2024JA033068.
https://doi.org/10.1029/2024JA033068
If GLEs do dominate, then the authors definitely need to include the more ubiquitous solar proton events in their analysis. Solar protons (distinct from GLEs) are an important source of ozone loss.
A list of solar proton events since the early 1980s (used in many studies) can be found at: https://www.ngdc.noaa.gov/stp/satellite/goes/dataaccess.html (solar proton flux data onboard GOES satellites)
Thanks for the comment. The mechanism is now better described (including the references indicated). We now also include an analysis showing only GLEs (this is a subset of our list, n=31). The signal in jet strength is stronger but also more variable. Finally, we also composited all other strong SPEs (>1000 pfu, n=16, ftp://ftp.swpc.noaa.gov/pub/indices/SPE.txt). They also show a strengthening of the jet. Of these 16, eight events were not already on our list and are now added.

We also composited potential EEP events (geomagnetic storms, low-latitude aurorae, and fast-transit events). They also show a strengthening of the polar vortex. Hence, we added five additional lines to the plots comprising pre- and post-1940 events, potential EEPs, GLEs, and other SPEs. All of them are consistent with a strengthening of the jet. Interestingly, GLEs and SPEs also show earlier peaks, whereas the potential EEPs and the pre-1940 cases (mostly EEPs) only show the peak at 80-100 days. This is consistent with the fact that GLEs and SPEs can penetrate further down, hence the downward transport and propagation is faster. However, different solar storms often overlap within the displayed 130-day period and a separation of mechanisms (beyond stating consistency) is difficult. Furthermore, the jet variability is very large.

Note also that we added a supplementary figure with case studies of the strongest event per category (asked for by Reviewer #1).

The revised figure is shown above. The altered text reads:

"Results show a significant strengthening of the jet after 30, 50, and 80-120 days (Fig. 3). The latter is robust when compositing subgroups (pre- and post-1940 events, potential EEPs, GLEs, or other SPEs, each centred to the subgroup period mean for Oct-Apr); GLEs and SPEs also show

earlier responses. Analyses of the strongest event per category (Fig. S9) are also consistent with the statistical results. The jet is shifted poleward on average, but not significantly."

4. Major Recommendation: Please report thresholds (and units if any) for the different measures. For example, what level of 'aa' index is associated with the listed geomagnetic storms? Similar information should be provided for other categories. The data should be available in the tables from which the events were taken and could be added to Table S5

Thanks for pointing to this omission, we added the thresholds used for all event categories to the caption:

Sources: A = Table 7 in Cliver et al. (2022) and B = Table 4 in Usoskin et al. (2023) for low-latitude aurorae, C = Table 2 in Lefèvre et al. (2016) for geomagnetic storms (peak aa > 300 nT), D = Table 5 in Gopalswamy et al. (2005) for fast transit events (<30 hours), E = Table 1 in Cliver et al. (2020) and F = Table 1 in Usoskin et al. (2020) for GLEs (statistically significant enhancements of count rates of at least two differently located neutron monitors), G = Table 7 in Hudson et al. (2024) for solar soft-X-ray flares (class X10, i.e., $>10^{-3}$ W m^{-2}), H = NOAA Space Weather Prediction Center for strong SPEs (>1000 pfu).

5. Major Recommendation 4: Please explain in general terms how the different solar storm categories produce ozone depletions or other phenomena relevant to the polar vortex and influence on the tropospheric jet. This could be added to the supplementary material if there is an issue with manuscript length. The effects of energetic particle (electron) precipitation from geomagnetic storms and the effects of solar proton events may have different time scale and paths ways from the upper atmosphere to the troposphere.
The mechanism is now better explained in the main text and we add several references (including a recent review paper). Common to all event types is the interaction with ozone chemistry via NOx (and at higher altitudes, HOx). The differences in time scale and pathways between EEP and SPE/GLE is also briefly explained. Since EEPs typically interact in the upper mesosphere (60-80 km), from where NOx needs to be transported downward to affect stratospheric ozone, while SPEs and GLEs can penetrate further down, we expect a longer delay for the former. There are in fact some indications for this in the composites, although the temporal overlap of events makes separation difficult (see comment above).

The altered text reads:

"Solar storms are often accompanied by increased solar energetic particle fluxes (including solar proton events, SPEs, and their extreme form: ground level enhancements GLEs) or by geomagnetic storms that trigger energetic electron precipitation (EEP) events. Both can generate HO_x or NO_x radicals in the polar mesosphere, typically at 60-80 km (energetic SPEs also further down), leading to ozone destruction both in the mesosphere and, through NO_x transport, the stratosphere.[33-35] Associated differential atmospheric heating strengthens the stratospheric polar vortex,[36] potentially leading to a strengthened and poleward shifted tropospheric jet.[37]. A more rapid, subtropical link could also shift the jet poleward.[33] Due to the higher altitude of the interaction, we expect a longer delay than for SSWs, presumably longer for EEP than SPEs."

With the suggested references plus the review:

Nesse, H. et al. Why is Energetic Particle Precipitation Important for Climate Research and Seasonal Forecasting? Surv. Geophys. (2026), https://doi.org/10.1007/s10712-026-09946-7

6. Minor Recommendation: In Table S5, the ‘fast transit events’ appear to describe fast eruptions from the Sun that intercept Earth. The table cited is attributed to Cliver, however I believe the table in this review article is directly copied from Gopalswamy (2005). Please check and attribute as appropriate. I believe this would be another name for what is currently called fast interplanetary shock events. Please elaborate and provide a threshold value for such events.
Thanks, this is correct. We now use the reference to Gopalswamy instead of Cliver. We add the threshold used by Gopalswamy, which is a transit time below 30 hours. Further we use the term interplanetary shock events along with fast transit events.

7. Major Recommendation: The authors cite Sukhodolov et al. as a single source of agreement/verification of the 70-80 day enhancement of jet strength. I recommend considering/including
Xu, X., Wang, Y., Wei, F. et al. The disrupted jet stream and its influence on flight time during solar proton events. Sci Rep 15, 22969 (2025). https://doi.org/10.1038/s41598-025-07137-9
I believe the results from the present study and the Xu et al study are consistent on shorter time frames, however Xu et al (2025) study a different longitude region and put more emphasis on latitudinal shift of the jet stream than enhancement of jet stream at higher latitudes. The Xu et al paper may contain important references for this study
Thanks, we added reference. Indeed, the studies are consistent although we find only small, non-significant poleward shift in our index. We add the reference to Xu as well as the following sentence:

“The jet is shifted poleward on average, but not significantly”

8. Question: Can the authors explain the significant time (and effect) differences between SSWs and solar storm effects?
SSWs start with a dynamical perturbation in the upper stratosphere. The time delay is the dynamical downward propagation mechanism through wave-mean flow interaction. In case of particles, the altitude of interaction is often higher, and there are chemical and radiative steps in between. For instance, NOx produced in the mesosphere must first be transported downward. We add a few more words on that. Further, we added a review be Nesse et al. (2026), published a month ago, which describes the mechanisms in more detail (see reply above).

We added the mechanism “wave-mean flow interaction” to the following sentence on SSWs:

“Major SSWs, defined as a collapse of the polar vortex in the upper stratosphere, can propagate downward by wave-mean flow interaction, eventually affecting the westerly circulation in the troposphere and extreme weather.” with a reference to Domeisen & Butler (see comment above).

And on solar storms:

“Both can generate HO_x or NO_x radicals in the polar mesosphere, typically at 60-80 km (energetic SPEs also further down), leading to ozone destruction both in the mesosphere and, through NO_x transport, the stratosphere.[33-35] Associated differential atmospheric heating strengthens the stratospheric polar vortex,[36] potentially leading to a strengthened and poleward shifted tropospheric jet.[37]”

9. Minor Recommendation for a possible extreme event that may support the analysis
The event from mid -January 1938 was deeply studied by Hayakawa et al. (2021). The intensity and

evolution of the extreme solar and geomagnetic storms in 1938 January. Astrophys J 909: 197.https://doi.org/10.3847/1538-4357/abc427. The event likely had multiply solar flares, and a sequence of magnetic storms that produced low latitude aurora for several nights, as well as a plausible 'solar particle storm'. Is there sufficient information from the compositing results to tell if this produced an intense tropospheric response?
Reviewer #1 asked for more case studies. We now add a supplementary figure showing four extreme events of each type, including the 1938 case. We also add the reference indicated. The results are consistent with the statistical analyses, although single events, even if strong, may not show the signal.

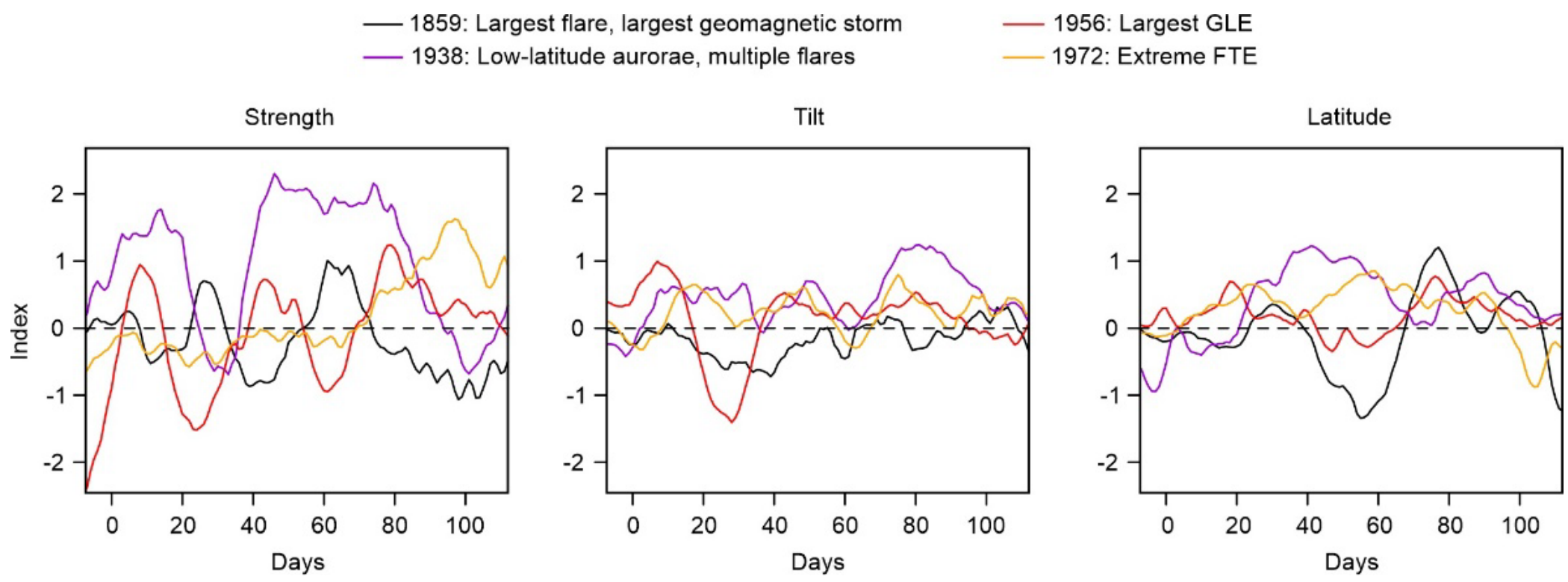

Fig. S9: 15-day averaged indices for the strongest solar storms. Shown are the Carrington event on 1 Sep 1859 (largest geomagnetic storm),[78-80] the low-latitude aurora 16 Jan 1938,84 GLE #5 on 23 Feb 1956,[85] and the fastest-transit event on 4 Aug 1972.[117]

Delores J Knipp

Reviewer #4 (Remarks on code availability):

I believe the request was for a review of the code written in R, which is not a software package/platform that I use. I do have a statistician colleague who works with R. I did not want to go outside of the review chain without checking with the editor.

Reviewer #5 (Remarks to the Author):

I think Brönnimann et al. present an interesting reconstruction of the Atlantic-European jet stream and possible influencing factors and effects, e.g. on extreme weather. The jet reconstruction and links to weather and extreme events is interesting and I have no major criticism there. I will focus my comments on the solar storm discussion which I find a little bit problematic. Nevertheless, after additional discussion of the uncertainties I think the manuscript can be recommended for publication.

I think the solar storm discussion is problematic as the lower panel in figure 3 (solar storm influence) is not very convincing to me. If there is a signal it might come after 80 days but why do the authors

cut the figure off at 90 days when the supposed strengthening is the strongest?

Thanks for the comment. We now extend the axis to 130 days, when the effect reaches zero again. The new figure is shown below in the reply to the next comment.

In my opinion an additional problem is the selection of solar storms. Energetic particle events are only known for the last about 80 years of neutron monitor data and these events are very different from geomagnetic storms. The authors write that both might affect ozone in the stratosphere but there are likely differences since energetic particle events involve mostly high-energy protons penetrating deeper into the atmosphere compared to electron precipitation events. To me it is not clear that both events should have the same effects (e.g altitude profile, effects on ozone etc are likely different). I think a more robust analysis would be to separate energetic particle events (the known ones) and the geomagnetic storm events (where we have data going further back & aurorae) to evaluate the possible effects and differences.

Thanks you for this suggestion. This comment is very much in line with the comments made by the other reviewers. It is true that the early events are presumably dominated by EEPs while later we also see SPEs, although also the earlier events might be associated to SPEs, as we have no observations. Also, the pathways are slightly different since the altitude of interaction is often higher (60-80 km) for EEPs than for SPEs, which can penetrate into the stratosphere. Hence, further mechanisms (downward transport of NOx) are required for EEPs which might lead to a longer delay. In the revised manuscript, we explain the differences better and give more references on this. Further, we perform sensitivity studies by separately analysing potential EEPs (low-latitude aurorae, geomagnetic storms, fast transit events), GLEs, and other SPEs. Also, we perform the analysis separately for pre- and post-1940s events. In fact, all sensitivity analyses show a strengthening of the jet, hence the analysis is robust. Indeed, potential EEPs show the main effect after 80-120 days, while GLEs and SPEs show also an earlier strengthening. However, due to the fact that storm events of different categories often overlap in time in the displayed 130-day window, a clear separation of mechanisms is not possible.

The new figure is shown here

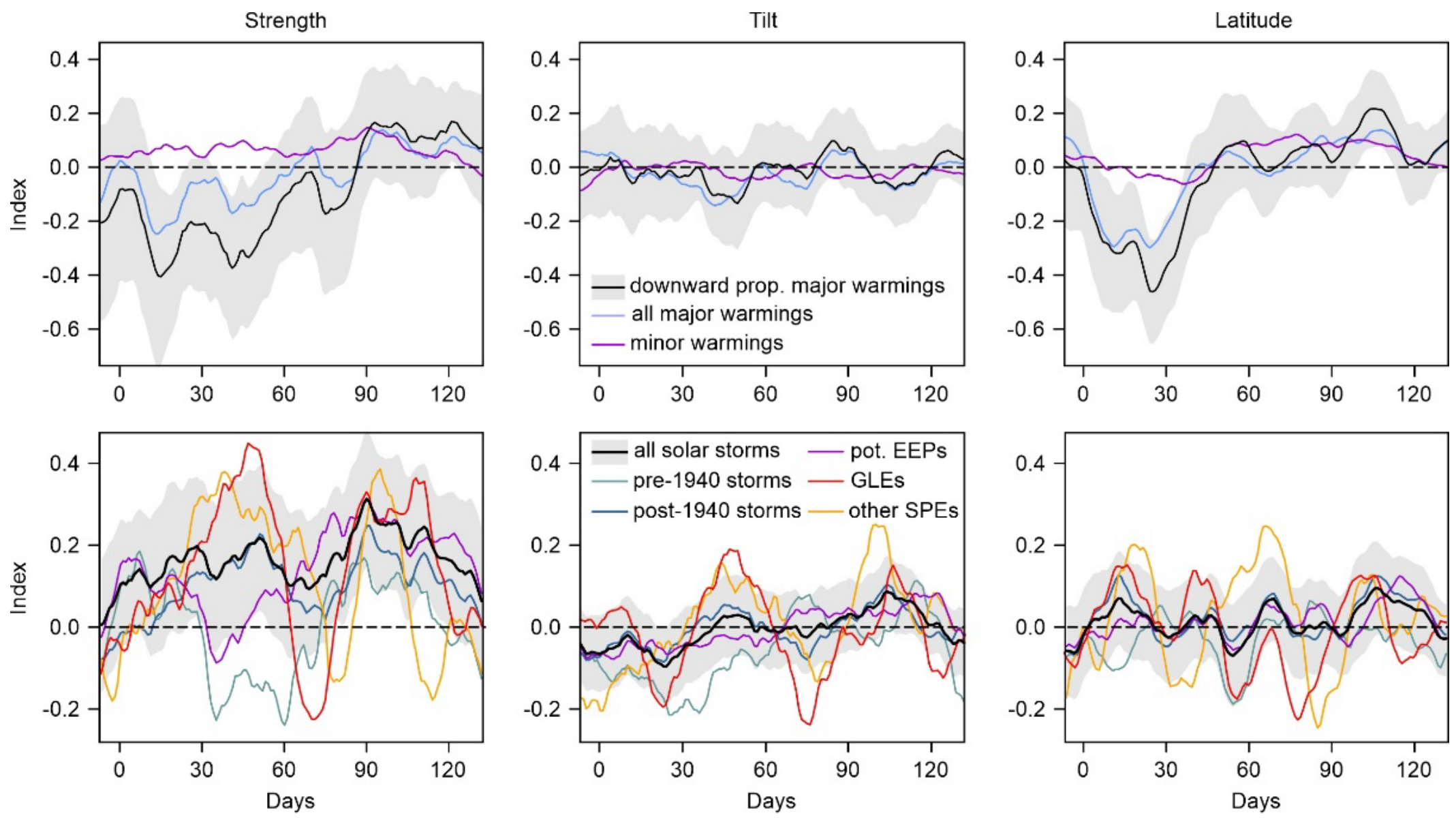

Fig. 3. Composites of 15-day averaged indices for SSWs or solar storms. Top: Composites for 55 major SSWs since 1940, of which 33 are characterised as downward propagating[31] (Table S4), and 176 minor warming events. Bottom: Composites for 107 solar or geomagnetic storms since 1730 that occurred in the September-March period (Table S5) as well as for five subgroups (pot. EEPs comprise low-latitude aurorae, geomagnetic storms, and fast transit events). Lines show the composite mean, shading indicates the 95% confidence interval of the mean.

And the text on the mechanisms is rewritten:

"Solar storms are often accompanied by increased solar energetic particle fluxes (including solar proton events, SPEs, and their extreme form: ground level enhancements GLEs) or by geomagnetic storms that trigger energetic electron precipitation (EEP) events. Both can generate HO_x or NO_x radicals in the polar mesosphere, typically at 60-80 km (energetic SPEs also further down), leading to ozone destruction both in the mesosphere and, through NO_x transport, the stratosphere.[33-35] Associated differential atmospheric heating strengthens the stratospheric polar vortex,[36] potentially leading to a strengthened and poleward shifted tropospheric jet.[37]. A more rapid, subtropical link could also shift the jet poleward.[33]"

With the new references:

35 Denton, M. H., Kivi, R., Ulich, T., Clilverd, M. A., Rodger, C. J., & von der Gathen, P. Northern hemisphere stratospheric ozone depletion caused by solar proton events: The role of the polar vortex. *Geophys. Res. Lett.* **45,** 2115–2124 (2018), https://doi.org/10.1002/2017GL075966

36 Li, H., Li, Y., Wang, Y., Sun, J., & Wang, C. Impact of solar proton events on the stratospheric polar vortex in the Northern Hemisphere: A quantitative analysis. *J. Geophys. Res.* **130,** e2024JA033068 (2025), https://doi.org/10.1029/2024JA033068.

37 Xu, X. et al. The disrupted jet stream and its influence on flight time during solar proton events. *Sci. Rep.* **15,** 22969 (2025). https://doi.org/10.1038/s41598-025-07137-9

Sukhodolov et al. might point to a possible mechanism but they model an event that is two orders of magnitude bigger than any know solar energetic particle event. So it is not at all clear that this can serve as an explanation for a solar influence on the strength of the jet.

Thanks, we now support the mechanism much better by citing additional literature. Four new references are given (see previous comment and below). Our results are consistent with the findings of these other studies, but we extend thee studies in time (and down to the surface).

To the sentence on Sukhodolov we added a sentence referring to review paper on the mechanisms:

Sukhodolov et al.[38] modelled the strongest known solar particle storm in 774/5 (assuming 1 Sep as date of the storm) and found strengthened westerlies at the surface after 70 days A review is given in Nesse et al.[39]

Reference:

Nesse, H. et al. Why is Energetic Particle Precipitation Important for Climate Research and Seasonal Forecasting? *Surv. Geophys.* (2026), https://doi.org/10.1007/s10712-026-09946-7

Reply to the Referee comments:

Reviewer #1 (Remarks to the Author):

This manuscript has been reasonably improved. I have only several comments for cosmetic improvements. I would expect no less from these authors.

Figure S9: Extreme FTE is misleading. Carrington event is also an EFT (transit time of ≈ 17.6 hours). The 1972 event should be the fastest transit event.
Thanks, the figure (now Extended Data Figure 9) is changed.

It is probably beneficial to put some more references that these reviews used to derive pertinent parameters, such as flare magnitude, Dst index, aa index, transit time, and auroral visibility extents.

1788 - DOI: 10.1093/pasj/psab079
1789 - DOI: 10.1016/j.asr.2021.05.001
1870 - DOI: 10.1029/2007JA012943
1872 - DOI: 10.3847/1538-4357/acc6cc
1882 - Replace Capron (1883) with DOI: 10.1002/2017SW001795
1940 - DOI: 10.1093/mnras/stab3615
1941 - DOI: 10.3847/1538-4357/abb772
1989 - DOI: 10.1029/2019SW002278
Thanks, we have added the references (though we feel a bit awkward having added, after all rounds of review, 10 references of the same first author). That said, we appreciate the references as they give more insight into the individual storms. Reading these papers and going through the list again, we realized that three of the cases added in response to the last review round do not fulfill the criterion of minimum 10 days distance. These are now combined. At the same time, since all analyses use a 15-day moving average, we decided to extend the minimum distance to 15 days, which affected two cases. Results did not change but are more consistent (n = 102 rather than n = 107).

Reviewer #4 (Remarks to the Author):

The authors have addressed all of my major concerns. In particular, they have separated Ground Level Enhancements (GELs) from the less energetic solar energetic particle events (SEP). They have performed sensitivity studies that include low latitude aurora, geomagnetic storms but higher flux,, and fast transit events. They find general consistency in the polar front jet stream response to these disturbance categories.
Thanks.

The authors have improved the supplementary table of solar event drivers (Table S5), and provided thresholds for each type of event.
They provide clear statements about the expected chain of events that connects earth-directted solar eruptions with polar vortex and jet stream dynamics.
Thanks.

Minor repair: Please note that references 68 and 69 are identical.
Changed.

I recommend publication of the present version of the article.

Delores Knipp

Reviewer #5 (Remarks to the Author):

I think this is an interesting study that provides useful insights into the past Atlantic-European jet stream and possible links to solar storms. The authors addressed all my comments to my satisfaction and I therefore recommend publication of the study.

I found a few smaller typos (resuls, minotors) and some missing or misplaced full stops (e.g. reference after full stop) that the authors should check again.

Thanks we went through the manuscript again and fixed some typos.
